# Oral Bacteriome and Mycobiome across Stages of Oral Carcinogenesis

**DOI:** 10.1128/spectrum.02737-22

**Published:** 2022-11-29

**Authors:** Weiwei Heng, Wenmei Wang, Tingting Dai, Ping Jiang, Yong Lu, Ruowei Li, Miaomiao Zhang, Ruiqi Xie, Yifan Zhou, Maomao Zhao, Ning Duan, Zhengqin Ye, Fuhua Yan, Xiang Wang

**Affiliations:** a Nanjing Stomatological Hospital, Medical School of Nanjing University, Nanjing, China; b Co-Innovation Center for the Sustainable Forestry in Southern China, Nanjing Forestry University, Nanjing, China; c Department of Endocrinology and Metabolism, Tongji Hospital, School of Medicine, Tongji University, Shanghai, China; Shenzhen Bay Laboratory

**Keywords:** bacteriome, mycobiome, carcinogenesis, oral cavity, plaque, saliva

## Abstract

Oral microbial dysbiosis contributes to the development of oral squamous cell carcinoma (OSCC). Numerous studies have focused on variations in the oral bacterial microbiota of patients with OSCC. However, similar studies on fungal microbiota, another integral component of the oral microbiota, are scarce. Moreover, there is an evidence gap regarding the role that microecosystems play in different niches of the oral cavity at different stages of oral carcinogenesis. Here, we catalogued the microbial communities in the human oral cavity by profiling saliva, gingival plaque, and mucosal samples at different stages of oral carcinogenesis. We analyzed the oral bacteriome and mycobiome along the health-premalignancy-carcinoma sequence. Some species, including Prevotella intermedia, Porphyromonas endodontalis, Acremonium exuviarum, and Aspergillus fumigatus, were enriched, whereas others, such as Streptococcus salivarius subsp. *salivarius*, Scapharca broughtonii, Mortierella echinula, and Morchella septimelata, were depleted in OSCC. These findings suggest that an array of signature species, including bacteria and fungi, are closely associated with oral carcinogenesis. OSCC-associated diversity differences, species distinction, and functional alterations were most remarkable in mucosal samples, not in gingival plaque or saliva samples, suggesting an urgent need to define oral carcinogenesis-associated microbial dysbiosis based on the spatial microbiome.

**IMPORTANCE** Abundant oral microorganisms constitute a complex microecosystem within the oral environment of the host, which plays a critical role in the adjustment of various physiological and pathological states of the oral cavity. In this study, we demonstrated that variations in the “core microbiome” may be used to predict carcinogenesis. In addition, sample data collected from multiple oral sites along the health-premalignancy-carcinoma sequence increase our understanding of the microecosystems of different oral niches and their specific changes during oral carcinogenesis. This work provides insight into the roles of bacteria and fungi in OSCC and may contribute to the development of early diagnostic assays and novel treatments.

## INTRODUCTION

Oral squamous cell carcinoma (OSCC), which arises from the mucosal lining of the oral cavity, is the most common form of head and neck malignancy (1, 2). Despite a high rate of relapse and poor prognosis (3, 4), the etiology of OSCC is incompletely understood. This highlights the importance of discovering additional pathogenic pathways for early detection and new treatment strategies of this deadly disease.

The oral mucosa not only is a vital physical barrier against pathogen invasion but also plays a key role in tumorigenesis (5, 6). Poor oral hygiene has been shown to affect the oral mucosa directly and to trigger the process of carcinogenesis (7, 8), suggesting that the role of the microbiome in oral carcinogenesis warrants investigation. The microbiome is increasingly recognized as a significant element of oral health. However, the host-microbiome interplay in the oral mucosa remains much less well understood than in other mucosal tissues such as the gastrointestinal and respiratory systems.

The bacterial community (bacteriome) and fungal community (mycobiome) constitute the microbiome, and most microbiome studies have focused on the bacteriome. In the 1990s, the discovery of Helicobacter pylori as a cause for gastric carcinoma showed that bacteria can be oncogenic (9). More recently, studies have shown that bacteria are associated with the pathogenesis of oral cancer (10, 11). However, little is known about another integral component of the oral microbiome, the commensal fungi in the oral cavity. Candida albicans, a common commensal fungus, thrives on a variety of human mucosa types and has been shown to be involved in oral carcinogenesis (12, 13). Moreover, interkingdom interactions between bacteria and fungi play an important role not only in healthy people but also in the initiation and progression of disease (14, 15). Thus, variations in the bacteriome and mycobiome across major stages of OSCC development require clarification.

Here, we performed DNA barcoding of two genetic markers, the 16S rRNA gene for the bacteriome and the internal transcribed spacer region (ITS) for the mycobiome, from multiple oral sites and saliva at different stages of oral carcinogenesis. Our approach focused on the identification of bacterial/fungal signature species that show distinct alterations across the stages of oral carcinogenesis.

## RESULTS

### Characteristics of the meta-analysis data sets.

In this study, we investigated 16S rRNA gene and ITS sequencing data to evaluate changes in the oral microbiome as OSCC progresses and to identify biomarkers specific to OSCC. In total, we collected 273 samples (90 from healthy controls [HC], 96 from patients with oral premalignant lesions [OPL subjects], and 87 from OSCC subjects). All samples were sequenced in sufficient depth. The total numbers of clean reads associated with the bacteriome and mycobiome were 26,865,824 and 27,513,625, respectively. There were 11,634 and 7,082 operational taxonomic unit (OTU) assignments for 16S and ITS sequencing, respectively. Consistent processing was performed for all raw sequencing data on the QIIME platform.

### Analyses of oral metacommunities.

**FIG 1:**
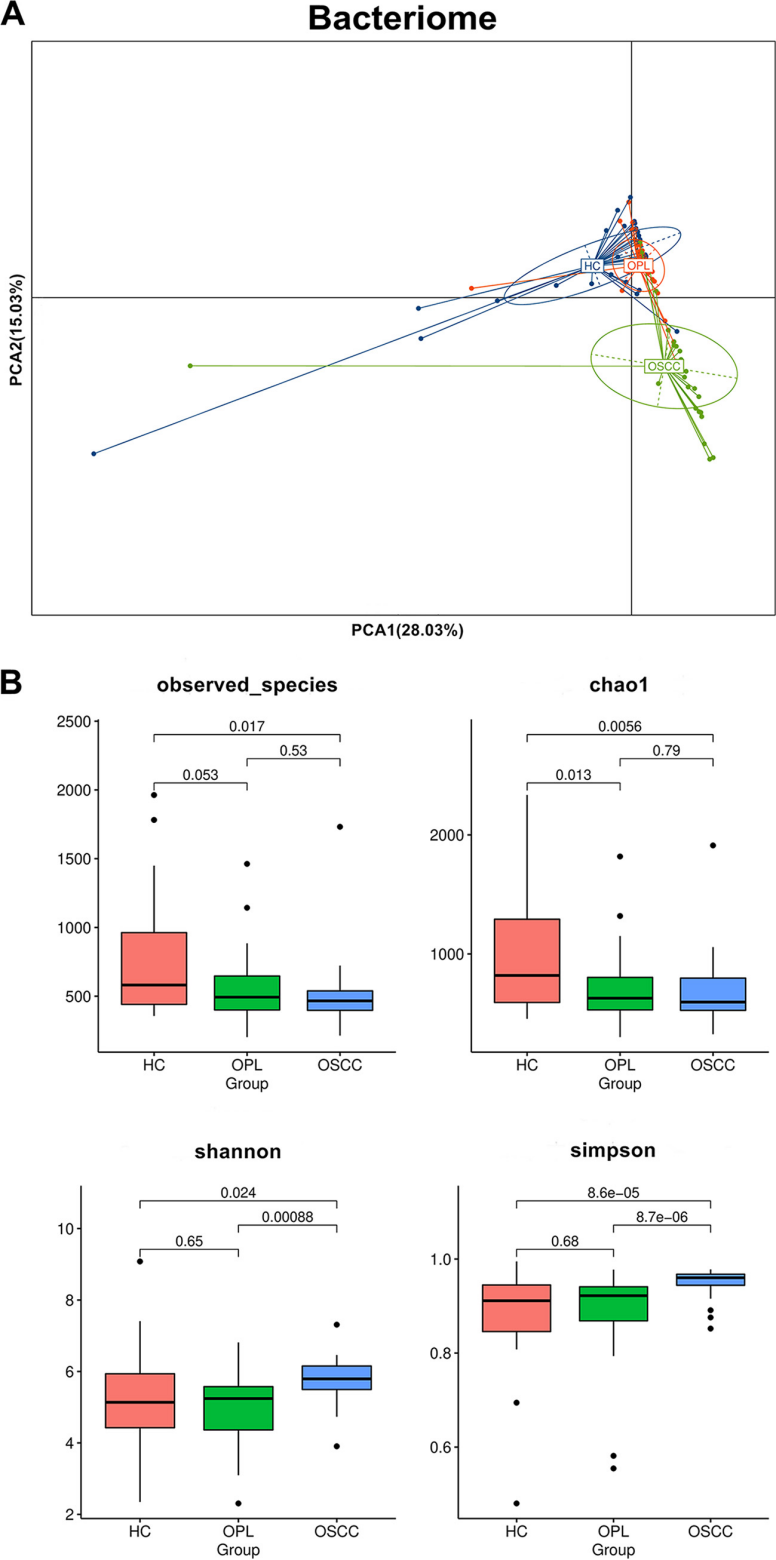
Principal-component analysis of the buccal mucosal bacteriomes from HC, OPL, and OSCC individuals. (A) The buccal mucosal bacteriome of individuals with OSCC was statistically significantly different (*P < *0.05) from that of HC and OPL individuals. (B) Box plots show the diversity and richness of the buccal mucosal bacteriomes from the HP, OPL, and OSCC groups at the OTU level.

Principal-component analysis (PCA) showed that for the bacteriome, OSCC samples were widely scattered, whereas HC and OPL samples were clustered together in buccal swabs (Fig. 1A), plaque swabs (see Fig. S1A in the supplemental material), and saliva (Fig. S2A). The bacterial richness in the OSCC and OPL groups was significantly lower than that in the HC group in buccal swabs (Fig. 1B) and saliva (Fig. S2B). The bacterial richness in the plaque swabs of the OPL group was significantly lower than that of the OSCC and HC groups (Fig. S1B). The diversity estimator Shannon index indicated that the relative diversity of bacterial genera in the buccal swabs was significantly increased in OSCC samples compared with that in the HC and OPL samples (Fig. 1B). The Shannon index indicated that the bacterial genera in the plaque swabs were more diverse in OSCC subjects than in OPL subjects (Fig. S1B). The Simpson index indicated that the relative diversity of the bacterial genera in the buccal (Fig. 1B) and plaque (Fig. S1B) swabs was significantly increased in OSCC subjects compared with that in HC and OPL subjects. Interestingly, the Shannon index showed a significant decrease in diversity estimator of in saliva samples from the OSCC and OPL patients compared with that in HC subjects (Fig. S2B). A similar trend of depletion in relative diversity of bacterial genera in the saliva of OSCC patients compared with HC subjects also was observed by employing the Simpson index (Fig. S2B).

**FIG 2:**
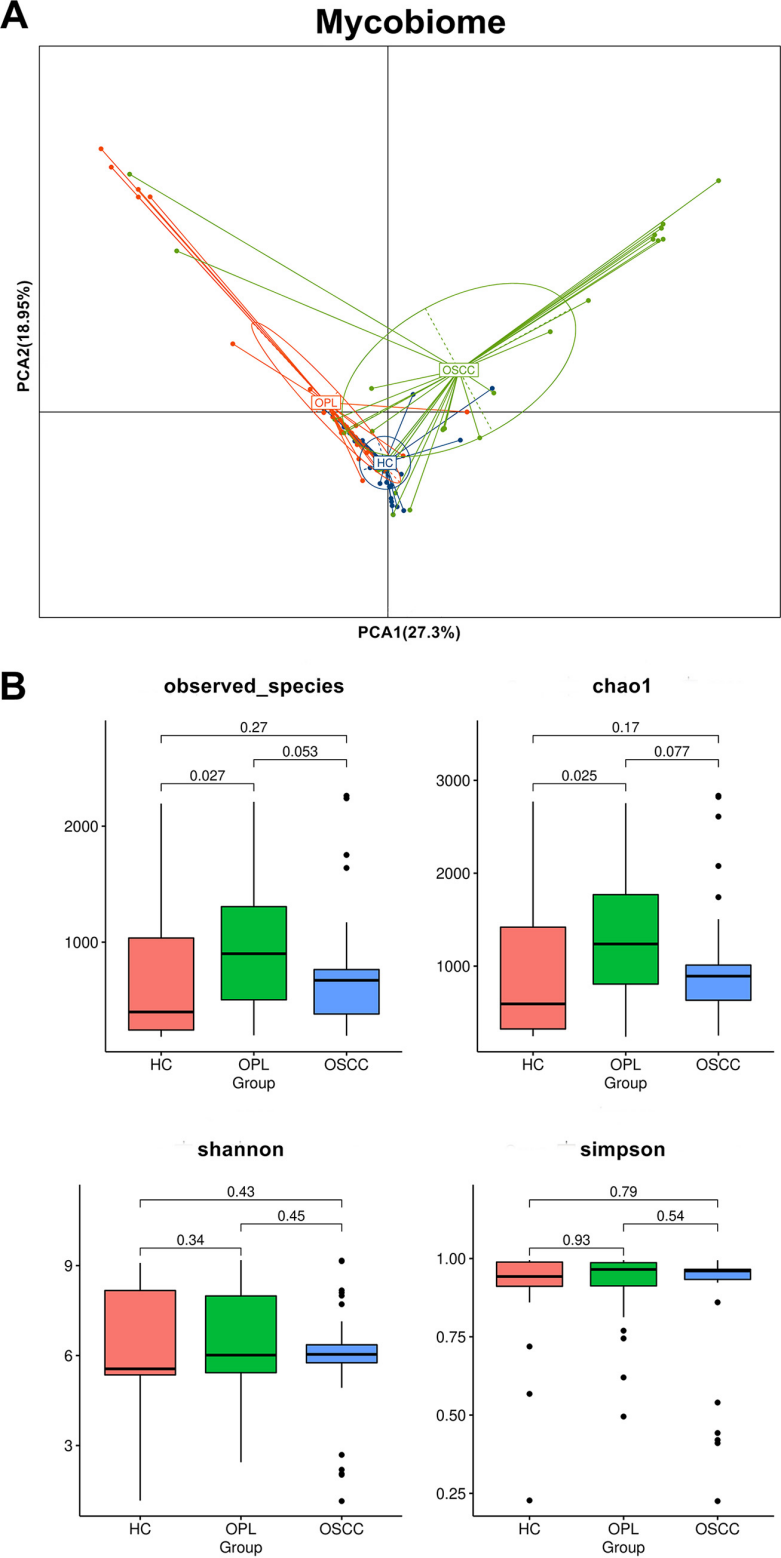
Principal-component analysis of the buccal mucosal mycobiomes from HC, OPL, and OSCC individuals. (A) The buccal mucosal mycobiome of individuals with OSCC was statistically significantly different (*P < *0.05) from that of HC and OPL individuals. (B) Box plots show the diversity and richness of the buccal mucosal mycobiomes from the HP, OPL, and OSCC groups at the OTU level.

PCA showed that for the mycobiome, OSCC samples were widely scattered, whereas HC and OPL samples were clustered together in the buccal (Fig. 2A) and plaque (Fig. S3A) swabs. In contrast, OSCC and healthy samples were clustered together, whereas the OPL samples were scattered in the saliva (Fig. S4A). The fungal richness in the buccal swabs of the OPL group was significantly higher than that in the HC group (Fig. 2B), and the richness in the plaque swabs of the OPL group was significantly higher than that in the OSCC group (Fig. S3B). Intriguingly, the richness in the saliva of the OPL group was significantly higher than that in the HC and OSCC groups (Fig. S4B). No significant difference in diversity estimator Shannon and Simpson indices was observed in buccal swabs (Fig. 2B), plaque swabs (Fig. S3B), or saliva samples (Fig. S4B) among all groups.

### Differentially abundant phyla and genera among the HC, OPL, and OSCC groups.

Of the bacterial phyla, *Firmicutes* was the most abundant. It was significantly decreased in the buccal and plaque swabs and saliva samples of the OSCC group compared with the HC group (25% versus 46%, *P < *0.001, false discovery rate [FDR] = 0.008; 25% versus 42%, *P < *0.001, FDR = 0.019; 19% versus 25%, *P = *0.013, FDR = 0.042, respectively) (Tables S2 to S4). It was also significantly decreased in the buccal and plaque swab samples of the OSCC group compared with the OPL group (25% versus 32%, *P = *0.035, FDR = 0.140; 25% versus 32%, *P = *0.011, FDR = 0.089, respectively) (Tables S2 and S3) and in the buccal and plaque swab samples of the OPL group compared with the HC group (32% versus 46%, *P = *0.002, FDR = 0.025; 32% versus 42%, *P* = 0.007, FDR = 0.053, respectively) (Tables S2 and S3). In addition, in the saliva samples, *Actinobacteria* was significantly decreased in the OSCC group compared with the HC group (3% versus 5%, *P = *0.003, FDR = 0.015) and in the OSCC group compared with the OPL group (3% versus 6%, *P < *0.001, FDR = 0.033) (Table S4). In the plaque swab samples, *Actinobacteria* was significantly decreased in the OSCC group compared with the OPL group (4% versus 7%, *P = *0.028, FDR = 0.089) (Table S3). In contrast, *Bacteroidetes* was significantly increased in buccal and plaque swab samples of the OSCC group compared with the HC group (30% versus 15%, *P < *0.001, FDR = 0.008; 24% versus 14%, *P < *0.001, FDR = 0.019, respectively) and in the buccal and plaque swab samples of the OSCC group compared with the OPL group (30% versus 15%, *P < *0.001, FDR = 0.007; 24% versus 14%, *P = *0.002, FDR = 0.072, respectively) (Tables S2 and S3). Moreover, *Fusobacteria* was significantly increased in the buccal swab samples of the OSCC group compared with the HC group (9% versus 3%, *P < *0.001, FDR = 0.008) (Table S2). Additionally, *Proteobacteria* was significantly increased in the buccal and plaque swab samples of the OPL group compared with the HC group (38% versus 27%, *P = *0.011, FDR = 0.102; 36% versus 29%, *P = *0.042, FDR = 0.103, respectively) (Tables S2 and S3). The fungal phylum Ascomycota was significantly decreased in the buccal swab and saliva samples of the OSCC group compared with the HC group (56% versus 65%, *P = *0.030, FDR = 0.195; 55% versus 66%, *P = *0.025, FDR = 0.133, respectively) but not in the plaque swab samples (Tables S5 to S7).

At the genus level, Streptococcus was the most abundant among the 161 significantly different bacterial genera in the buccal and plaque swab samples (Tables S8 and S9). Streptococcus was significantly decreased in the buccal and plaque swabs and the saliva samples of the OSCC group compared with the HC group (8% versus 29%, *P < *0.001, FDR = 0.017; 12% versus 24%, *P < *0.001, FDR = 0.014; 7% versus 9%, *P = *0.048, FDR = 0.163, respectively) (Tables S8 to S10). In the buccal and plaque swab samples, Streptococcus was also significantly decreased in the OSCC group compared with the OPL group (8% versus 18%, *P < *0.001, FDR = 0.014; 12% versus 19%, *P = *0.002, FDR = 0.023, respectively) (Tables S8 and S9). In the buccal swab samples, Streptococcus was also significantly decreased in the OPL group compared with the HC group (18% versus 29%, *P = *0.006, FDR = 0.080) (Table S8). *Veillonella* was significantly decreased in all three sample types (buccal swab, plaque swab, and saliva) of the OSCC group compared with the HC group (4% versus 7%, *P = *0.036, FDR = 0.164; 5% versus 9%, *P < *0.001, FDR = 0.014; 5% versus 9%, *P < *0.001, FDR = 0.021, respectively) (Tables S8 to S10). In the plaque swab samples, *Veillonella* was also significantly decreased in the OPL group compared with the HC group (5% versus 9%, *P < *0.001, FDR = 0.014) (Table S9). In contrast, in the buccal swab samples, five bacterial genera (*Alloprevotella*, *Fusobacterium*, *Prevotella*, *Aggregatibacter*, and *Capnocytophaga*) were significantly increased in the OSCC group compared with the HC group (9% versus 3%, *P < *0.001, FDR = 0.017; 7% versus 2%, *P < *0.001, FDR = 0.017; 7% versus 2%, *P < *0.001, FDR = 0.017; 4% versus 1%, *P < *0.001, FDR = 0.017; and 4% versus 1%, *P < *0.001, FDR = 0.017, respectively) and the OPL group (9% versus 4%, *P = *0.002, FDR = 0.022; 7% versus 3%, *P < *0.001, FDR = 0.014; 7% versus 3%, *P = *0.004, FDR = 0.035; 4% versus 1%, *P = *0.002, FDR = 0.022; and 4% versus 1%, *P < *0.001, FDR = 0.014, respectively) (Table S8). Furthermore, in the plaque swab samples, three bacterial genera (*Alloprevotella*, *Capnocytophaga*, and *Prevotella*) were significantly increased in the OSCC group compared with the HC group (8% versus 3%, *P = *0.005, FDR = 0.0340; 6% versus 2%, *P = *0.003, FDR = 0.029; 3% versus 1%, *P = *0.004, FDR = 0.035, respectively) and two bacterial genera (*Alloprevotella* and *Capnocytophaga*) were significantly increased in the OSCC group compared with the OPL group (8% versus 3%, *P = *0.006, FDR = 0.044; 6% versus 2%, *P = *0.029, FDR = 0.142, respectively) (Table S9). In addition, in the saliva samples, three bacterial genera (*Neisseria*, *Aggregatibacter*, and *Capnocytophaga*) were significantly increased in the OSCC group compared with the HC group (20% versus 12%, *P = *0.024, FDR = 0.110; 3% versus 1%, *P < *0.001, FDR = 0.069; 2% versus 1%, *P = *0.003, FDR = 0.036, respectively) and *Capnocytophaga* was significantly increased in the OSCC group compared with the OPL group (2% versus 1%, *P = *0.014, FDR = 0.173) (Table S10). Moreover, *Neisseria* was significantly increased in the buccal swab and saliva samples of the OPL group compared with the HC group (15% versus 9%, *P = *0.030, FDR = 0.195; 18% versus 12%, *P = *0.017, FDR = 0.085, respectively) (Tables S8 and S10).

*Morchella* was the most abundant fungal genus in the buccal and plaque swabs and the saliva samples of the HC group (15%, 15%, and 17%, respectively), while *Clitopilus* had the highest abundance in the OSCC group (11%, 11%, and 11%, respectively) (Tables S11 to S13). *Morchella* was significantly decreased in the plaque swab and saliva samples of the OSCC group compared with the HC group (6% versus 15%, *P = *0.039, FDR = 0.222; 5% versus 17%, *P = *0.018, FDR = 0.118, respectively) (Tables S12 and S13). However, *Candida* was significantly increased in the buccal and plaque swabs and the saliva samples of the OSCC group compared with the HC group (5% versus 1%, *P = *0.002, FDR = 0.025; 5% versus 1%, *P = *0.007, FDR = 0.074; 5% versus 1%, *P = *0.012, FDR = 0.099, respectively) and significantly increased in the plaque swab samples of the OSCC group compared with the OPL group (5% versus 1%, *P = *0.008, FDR = 0.092) (Tables S11 to S13). *Acremonium* was also significantly increased in the buccal and plaque swab samples of the OSCC group compared with the HC group (3% versus 1%, *P = *0.002, FDR = 0.025; 3% versus 1%, *P = *0.004, FDR = 0.049, respectively), as well as in the buccal and plaque swab samples of the OSCC group compared with the OPL group (3% versus 1%, *P < *0.001, FDR = 0.016; 3% versus 0.4%, *P < *0.001, FDR = 0.044, respectively) (Tables S11 and S12). In addition, Aspergillus was significantly increased in the plaque swab and saliva samples of the OSCC group compared with the HC group (2% versus 1%, *P = *0.010, FDR = 0.084; 3% versus 1%, *P* = 0.003, FDR = 0.036, respectively) (Tables S12 and S13), as well as in the buccal and plaque swabs and the saliva samples of the OSCC group compared with the OPL group (3% versus 2%, *P = *0.007, FDR = 0.063; 2% versus 1%, *P* = 0.031, FDR = 0.203; 3% versus 2%, *P = *0.008, FDR = 0.080, respectively) (Tables S11 to S13).

### Ternary plot and bubble plot results.

**FIG 3:**
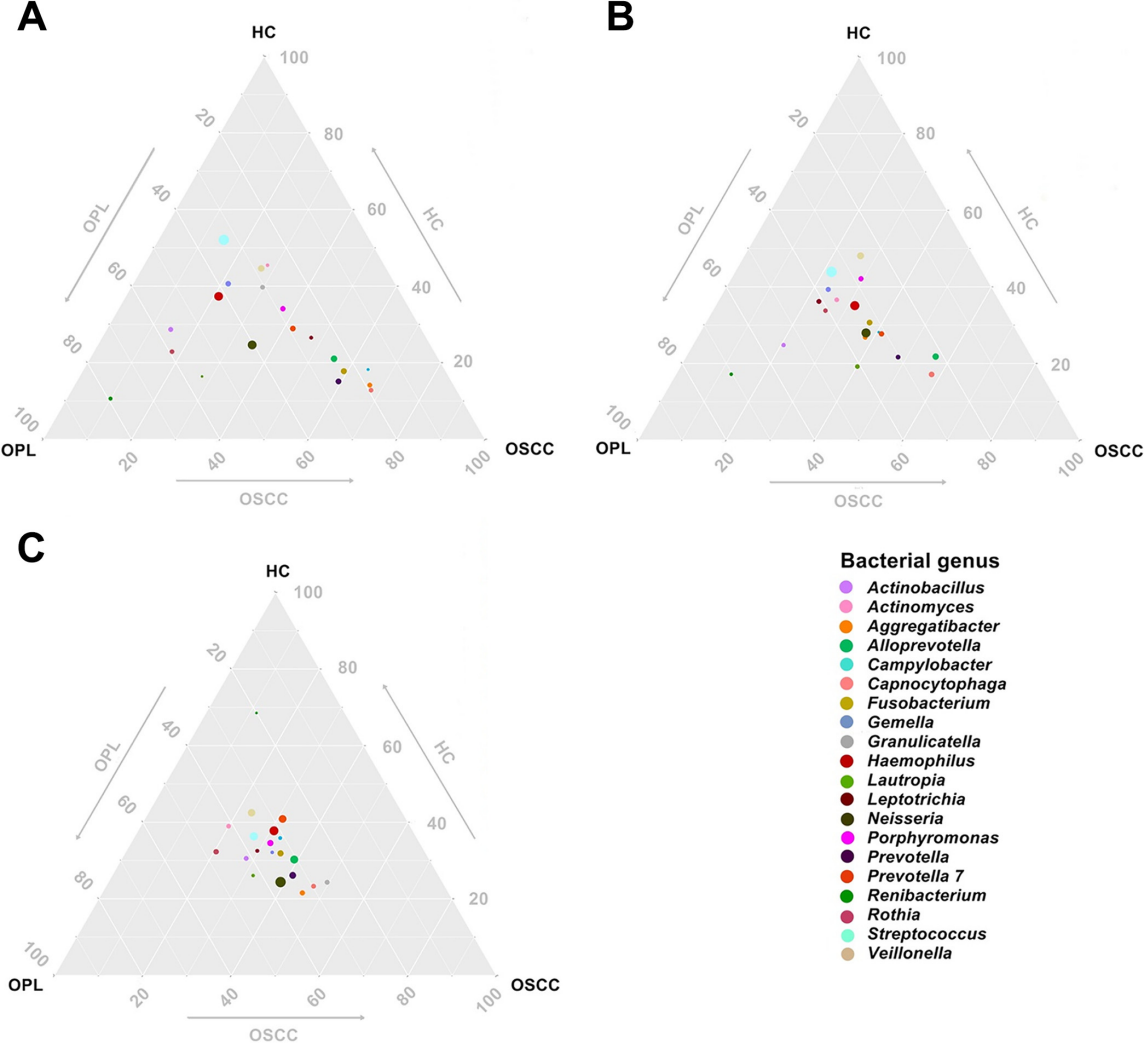
Ternary plot of the oral bacteriome derived from all studied subjects in the HC, OPL, and OSCC groups. The plots represent different samples from the buccal mucosa (A), plaque (B), and saliva (C). The position of the bubble denotes a close correlation of the genera within each group. The arrows indicate the direction of oral carcinogenesis.

**FIG 4:**
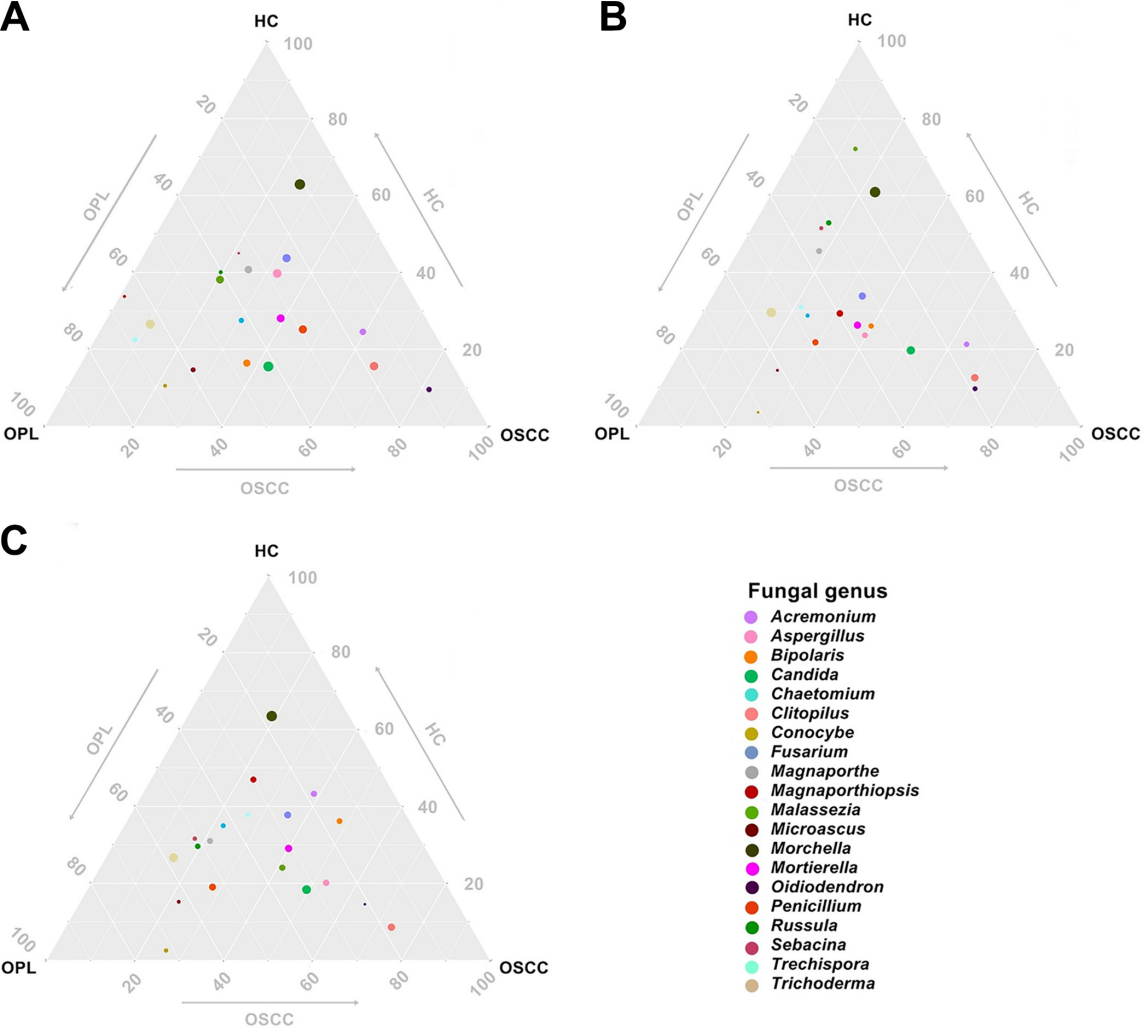
Ternary plot of the oral mycobiome derived from all studied subjects in the HC, OPL, and OSCC groups. The plots represent different samples from the buccal mucosa (A), plaque (B), and saliva (C). The position of the bubble denotes a close correlation of the genera within each group. The arrows indicate the direction of oral carcinogenesis.

A ternary plot was generated to analyze the differential and same species based on the top 20 bacterial and fungal genus annotation data. In the buccal swab samples, the bacterial genera *Capnocytophaga*, *Aggregatibacter*, Campylobacter, *Prevotella*, *Fusobacterium*, and *Alloprevotella* were close to OSCC but far from HC and OPL. Streptococcus, *Veillonella*, and *Actinomyces* were close to HC but far from OSCC and OPL (Fig. 3A). In the plaque samples, the bacterial genera *Capnocytophaga*, *Alloprevotella*, and *Prevotella* were close to OSCC but far from HC and OPL. *Veillonella* was close to HC but far from OSCC and OPL (Fig. 3B). In the saliva samples, all bacterial genera were located at the middle position, indicating that they had a similar abundance and composition across the three groups (Fig. 3C). In the buccal and plaque swabs and the saliva samples, the fungal genera *Oidiodendron*, *Clitopilus*, and *Acremonium* were close to OSCC but far from HC and OPL. *Morchella* was close to HC but far from OSCC and OPL (Fig. 4).

**FIG 5:**
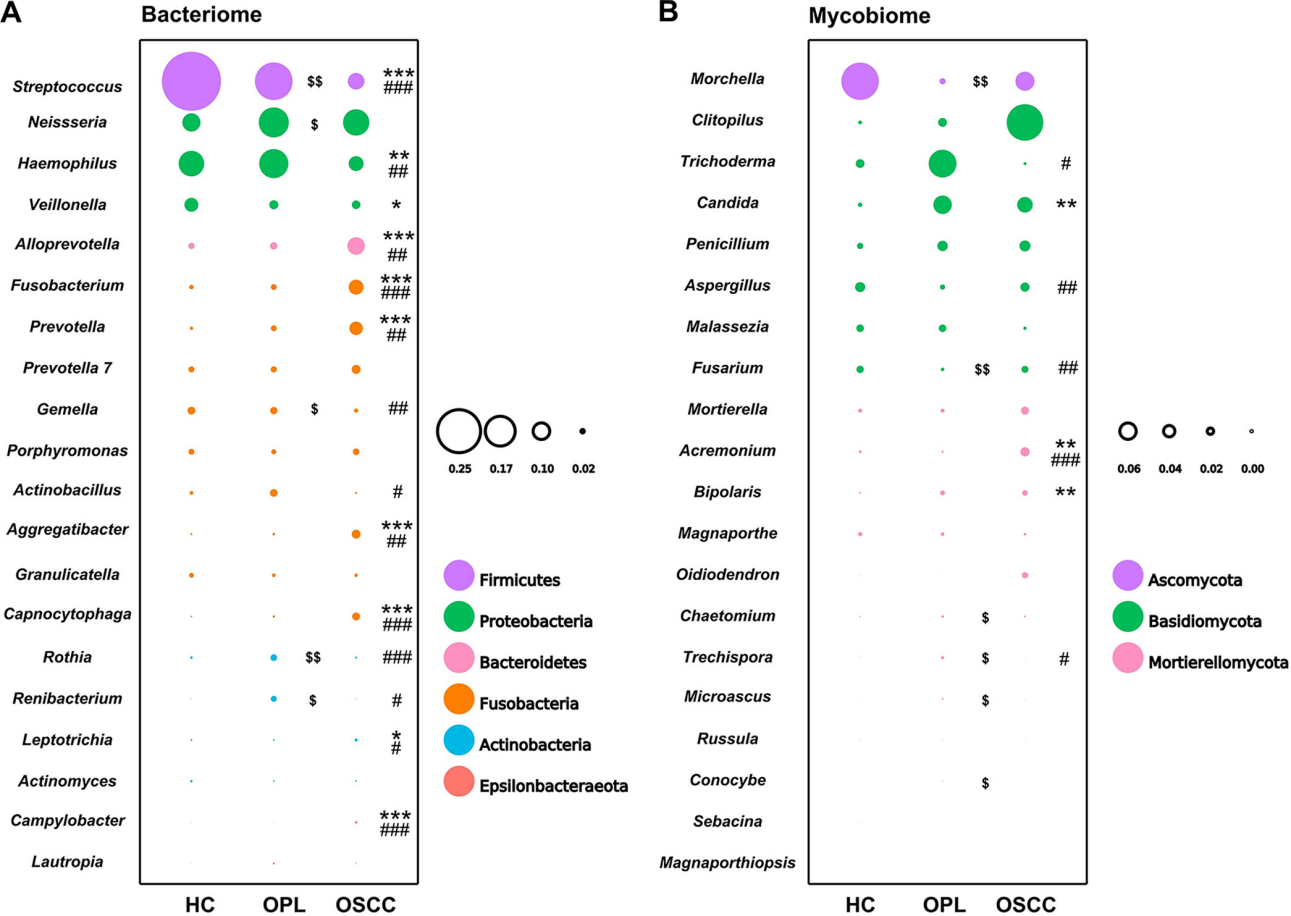
Bubble plot of the buccal mucosal bacteriome (A) and mycobiome (B) from HC, OPL, and OSCC individuals. Each bubble represents one genus, and the bubble size represents the relative abundance of each genus. For the OSCC group, *, **, and *** denote *P *values of <0.05, <0.01, and <0.001 versus the HC group, respectively. For the OSCC group, #, ##, and ### denote *P *values of <0.05, <0.01, and <0.001 versus the OPL group, respectively. For the OPL group, $ and $$ denote *P *values of <0.05 and <0.01 versus the HC group, respectively.

According to the bubble plot, the bacterial genus *Capnocytophaga* and the fungal genus *Candida* were enriched in the buccal and plaque swabs and the saliva samples of OSCC patients, while the bacterial genera Streptococcus and *Veillonella* and the fungal genus *Trichoderma* were depleted in all three types of samples of OSCC patients (Fig. 5; Fig. S5 and S6). The bacterial genera *Prevotella* and *Alloprevotella* and the fungal genus *Acremonium* were enriched in the buccal and plaque swab samples of OSCC patients, while the bacterial genus *Gemella* was depleted in both types of samples of OSCC patients (Fig. 5; Fig. S5). The bacterial genus *Aggregatibacter* and the fungal genus *Bipolaris* were enriched in the buccal swab and saliva samples of OSCC patients, whereas the bacterial genus *Rothia* was depleted in both types of samples of OSCC patients (Fig. 5; Fig. S6). The bacterial genera *Fusobacterium* and *Leptotrichia* were enriched in the buccal swab samples of OSCC patients, whereas the bacterial genus Haemophilus was depleted in these samples (Fig. 5A). The bacterial genera *Porphyromonas* and *Leptotrichia* were depleted in the plaque swab samples of OSCC patients (Fig. S5A). The bacterial genus *Neisseria* was enriched in the saliva samples of OSCC patients, whereas *Actinomyces* was depleted in these samples (Fig. S6A). The fungal genus *Morchella* was depleted in the plaque swab and saliva samples of OSCC patients (Fig. S5B and S6B).

### Taxonomic biomarkers in the oral microbiota during oral carcinogenesis.

**FIG 6:**
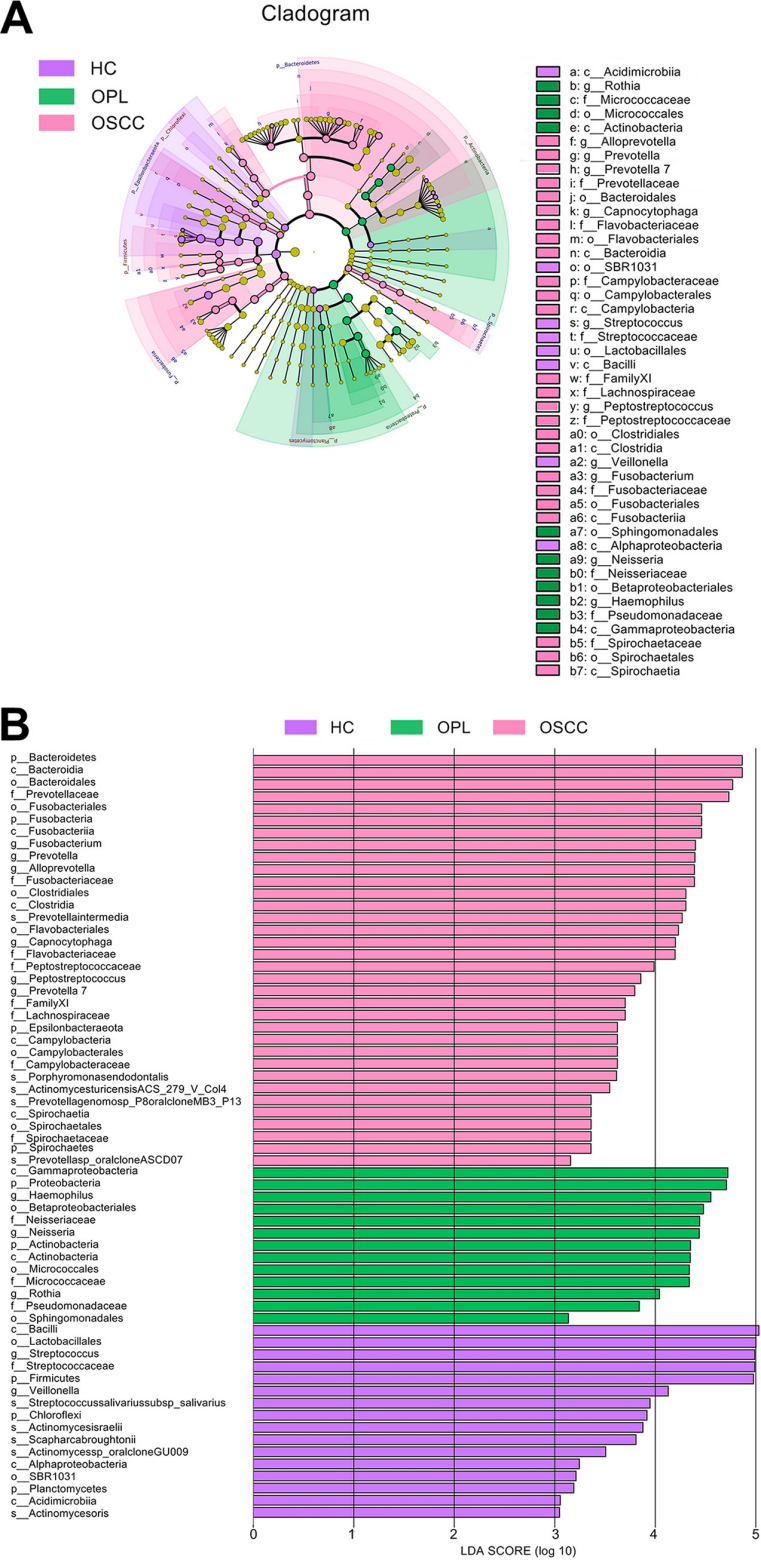
Oral carcinogenesis-associated alterations in the abundance of the buccal mucosal bacteria examined using LEfSe. (A) Cladogram indicating the phylogenetic distribution of active bacteria that were remarkably enriched. (B) Bar plots at the species level with significant differences in abundance based on LEfSe. The colored bars show the LDA scores of species that were enriched in the indicated condition: purple bar, HC; green bar, OPL; pink bar, OSCC.

The linear discriminant analysis (LDA) effect size (LEfSe) method was used to identify differentially enriched bacterial species within groups. In the buccal swab samples, Prevotella intermedia, Porphyromonas endodontalis, Actinomyces turicensis ACS-279-V-Col4, *Prevotella genomo* sp. P8 oral clone MB3 P13, and *Prevotella* sp. oral clone ASCD07 were the species of highest abundance in the OSCC patients, where Streptococcus salivarius subsp. *salivarius*, Actinomyces israelii, *Scapharca broughtonii*, *Actinomyces* sp. oral clone GU009, and Actinomyces oris were abundantly associated with the HC subjects (Fig. 6). In the plaque swab samples, Prevotella intermedia, Porphyromonas endodontalis, and *Prevotella* sp. oral clone FW035 were the most significantly abundant species in the OSCC patients, and Aggregatibacter aphrophilus ATCC 33389 and *Leptotrichia* sp. oral taxon 847 were most abundant in the OPL patients. In addition, Streptococcus salivarius subsp. *salivarius*, Actinomyces weissii, *Scapharca broughtonii*, *Actinomyces* sp. oral clone GU009, and *Porphyromonas* sp. oral clone HF001 were the most abundant in the HC subjects (Fig. S7). In saliva samples, Prevotella intermedia was the most abundant in the OSCC patients and *Leptotrichia* sp. oral taxon 847 was the most abundant in the OPL patients, whereas Actinomyces oris was mostly associated with the HC subjects (Fig. S8).

**FIG 7:**
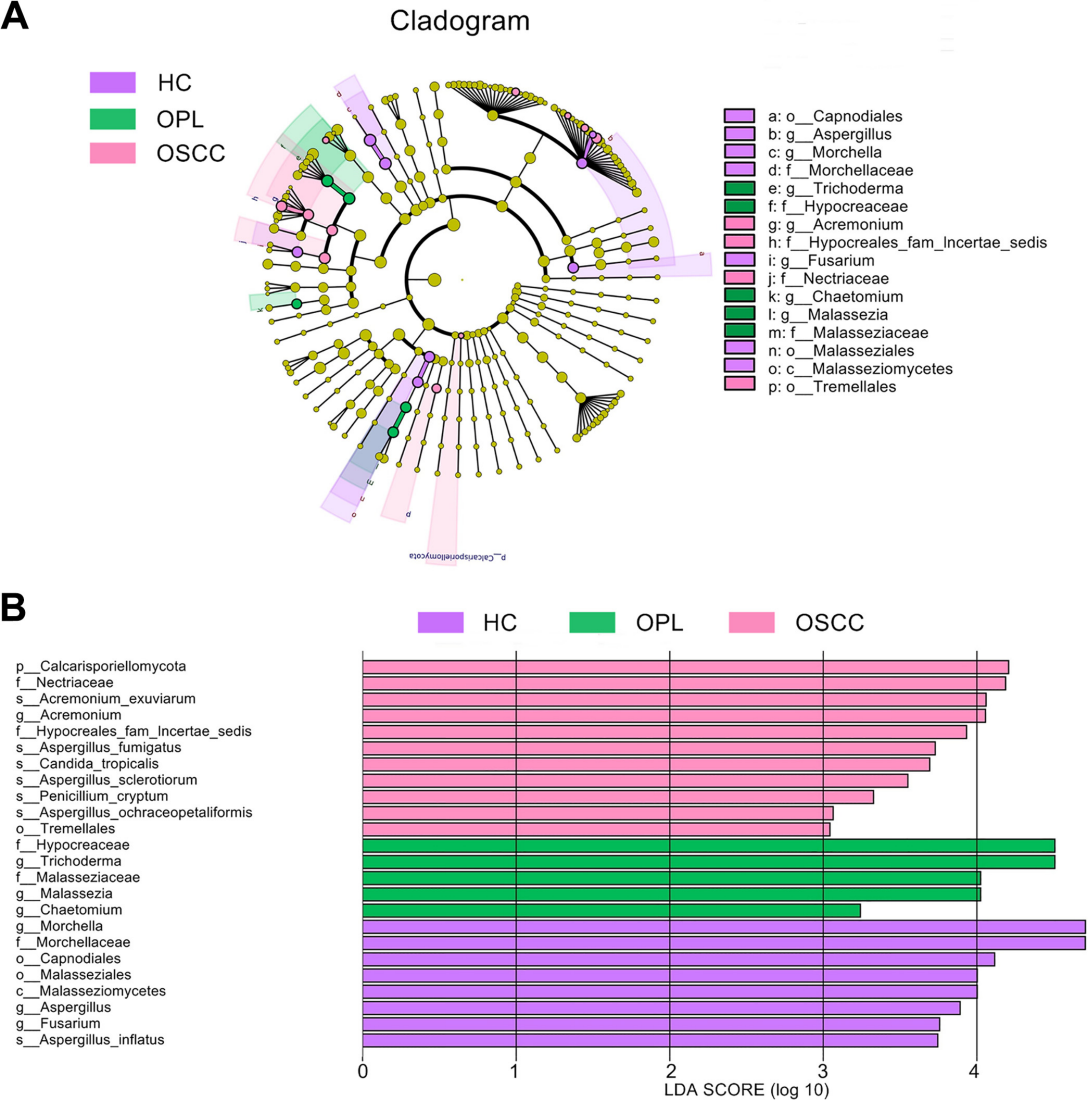
Oral carcinogenesis-associated alterations in the abundance of the buccal mucosal fungi examined using LEfSe. (A) Cladogram indicating the phylogenetic distribution of active fungi that were remarkably enriched. (B) Bar plots at the species level with significant differences in abundance based on LEfSe. The colored bars show the LDA scores of species that were enriched in the indicated condition: purple bar, HC; green bar, OPL; pink bar, OSCC.

For differentially enriched fungal species within groups, the LEfSe results indicated that *Acremonium exuviarum*, Aspergillus fumigatus, Candida tropicalis, Aspergillus sclerotiorum, Penicillium cryptum, and Aspergillus ochraceopetaliformis were the most significantly abundant in the buccal swab samples of the OSCC group, while Aspergillus inflatus was associated mainly with the HC group (Fig. 7). In the plaque swab samples, *Acremonium exuviarum*, Aspergillus *ochraceopetaliformis*, Aspergillus fumigatus, and *Penicillium cryptum* were the most significantly abundant species in the OSCC patients, Trichoderma asperellum, Trichoderma koningiopsis, and Penicillium simplicissimum were most abundant in the OPL patients, and Penicillium bialowiezense, Aspergillus aculeatus, Chaetomium atrobrunneum, Acremonium persicinum, Oidiodendron rhodogenum, Aspergillus tamarii, Mortierella echinula, Aspergillus flavus, and Trichoderma longibrachiatum were mostly associated with the HC subjects (Fig. S9). In the saliva samples, *Acremonium exuviarum*, Aspergillus fumigatus, and Cephalotrichum purpureofuscum were the most significantly abundant in the OSCC patients, Acremonium curvulum was the most abundant in the OPL patients, and Morchella septimelata was mostly associated with the HC subjects (Fig. S10).

### Intra- and interkingdom correlation analyses in oral carcinogenesis.

Next, we conducted intra- and interkingdom correlation analyses with bacterial and fungal taxa in the microbiota of healthy, premalignant, and malignant samples. At the bacterial genus level, Streptococcus exhibited a negative intragenus correlation with Haemophilus and *Veillonella* in the buccal and plaque samples of the HC group (Fig. S11A and S12A). Alternatively, the intragenus correlation was positive in the buccal and plaque samples of the OSCC group (Fig. S11C and S12C). We also found that the robust positive correlation between *Aggregatibacter* and *Capnocytophaga* was depleted in all three sample types during OSCC development (Fig. S11A to C, S12A to C, and S13A to C). At the fungal genus level, the negative intragenus correlation between *Candida* and *Acremonium* became positive in the buccal swabs and the saliva samples during oral carcinogenesis (Fig. S11D to F and S13D to F). In contrast, the positive intragenus correlation between *Candida* and *Mortierella* became negative in all sample types during oral carcinogenesis (Fig. S11D to F, S12D to F, and S13D to F). We also found that the positive correlation between *Morchella* and *Clitopilus* became negative in all sample types during OSCC development (Fig. S11D to F, S12D to F, and S13D to F).

At the fungal genus level, Streptococcus was positively correlated with *Morchella* and *Mortierella* in the buccal swab samples of the HC group (Fig. S14A). However, these correlations were negative during OSCC carcinogenesis (Fig. S14B and C). *Aggregatibacter* was positively correlated with *Morchella* in the buccal swab samples of the HC group, whereas the correlation was negative in the OPL and OSCC groups (Fig. S14). Streptococcus was negatively correlated with *Candida* in the buccal swab samples of the HC and OPL groups, whereas the correlation was positive in the OSCC group (Fig. S14). *Gemella* correlated negatively with *Candida* in the buccal swab samples of the HC group, whereas the correlation was positive in the OPL and OSCC groups (Fig. S14). In the plaque samples, the positive correlation between Streptococcus and *Mortierella* was increasingly robust during OSCC development (Fig. S15). In plaque samples, the positive correlations of *Alloprevotella* with *Morchella* and *Clitopilus* became negative during OSCC development (Fig. S15). Alternatively, the negative correlation between *Capnocytophaga* and *Morchella* and *Clitopilus* was positive during OSCC development (Fig. S15). Additionally, the positive correlation between *Capnocytophaga* and *Candida* was negative during OSCC development (Fig. S15). In all sample types, *Veillonella* correlated positively with *Morchella* and *Clitopilus* in the HC group, whereas the correlation was negative in the OPL and OSCC groups (Fig. S14 to S16). Moreover, the robust negative correlation between *Veillonella* and *Candida* decreased in saliva samples during OSCC tumorigenesis (Fig. S14 to S16). Furthermore, the negative correlation between *Veillonella* and *Mortierella* and *Trichoderma* became increasingly positive in saliva samples during OSCC development (Fig. S16).

### ROC curve analyses in OSCC.

**FIG 8:**
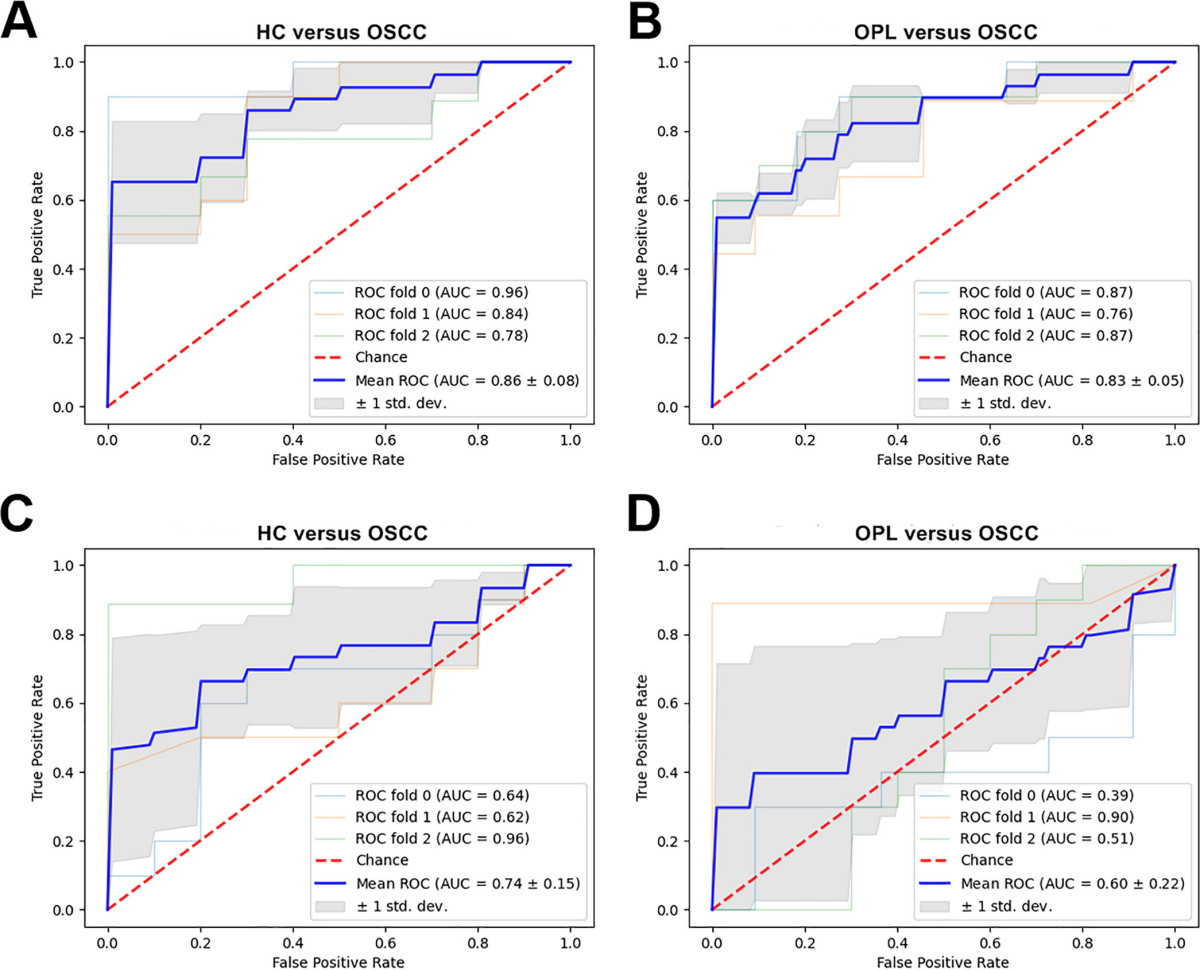
AUC of the optimized models constructed with bacterial (A and B) and fungal (C and D) biomarkers and patient metadata of HC versus OPL and OPL versus OSCC. Mean AUCs and standard deviations of stratified 3-fold cross-validation are shown.

The efficacy of these differentially expressed bacteria and fungi in discriminating across OSCC stages was calculated using the receiver operating characteristic (ROC) curve. Among the top 20 bacterial genera, based on the LEfSe results, we selected a bacterial signature panel with 10 species (Streptococcus salivarius subsp. *salivarius*, Actinomyces israelii, *Scapharca broughtonii*, *Actinomyces* sp. oral clone GU009, Actinomyces oris, Prevotella intermedia, Porphyromonas endodontalis, Actinomyces turicensis ACS-279-V-Col4, *Prevotella genomo* sp. P8 oral clone MB3 P13, and *Prevotella* sp. oral clone ASCD07) that had an AUC of 0.86 in discriminating OSCC from HC (Fig. 8A) and an AUC of 0.83 in discriminating OSCC from OPL (Fig. 8B). Furthermore, the fungal species *Acremonium exuviarum* had an AUC of 0.74 in discriminating OSCC from HC (Fig. 8C) and an AUC of 0.60 in discriminating OSCC from OPL (Fig. 8D).

### Microbial functional changes in oral carcinogenesis.

**FIG 9:**
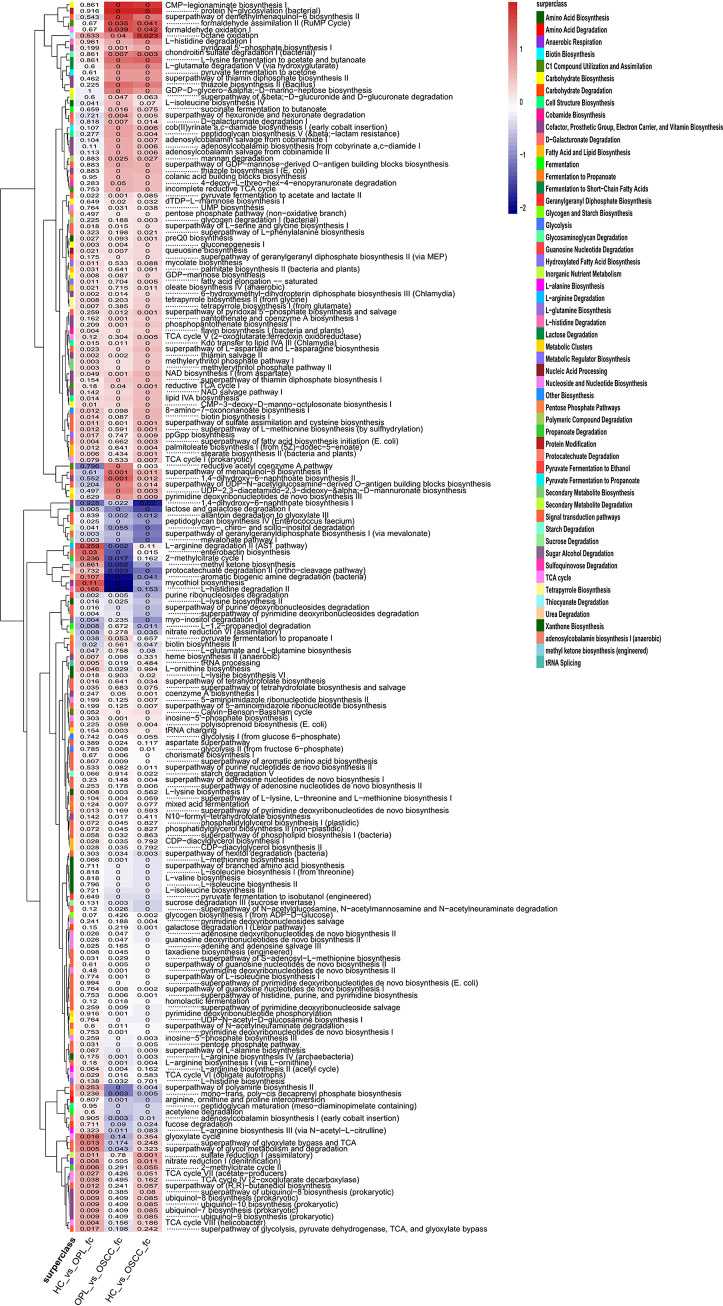
Functional alterations in the buccal mucosal bacteriome. The relative abundances of functional pathways were compared among HC, OPL, and OSCC individuals. Differentially abundant pathways were plotted, and the exact *P* values are presented in the heat map. The generalized fold change is indicated by color gradients. A generalized fold change of >0 means enriched in the latter; a generalized fold change of <0 means enriched in the former.

**FIG 10:**
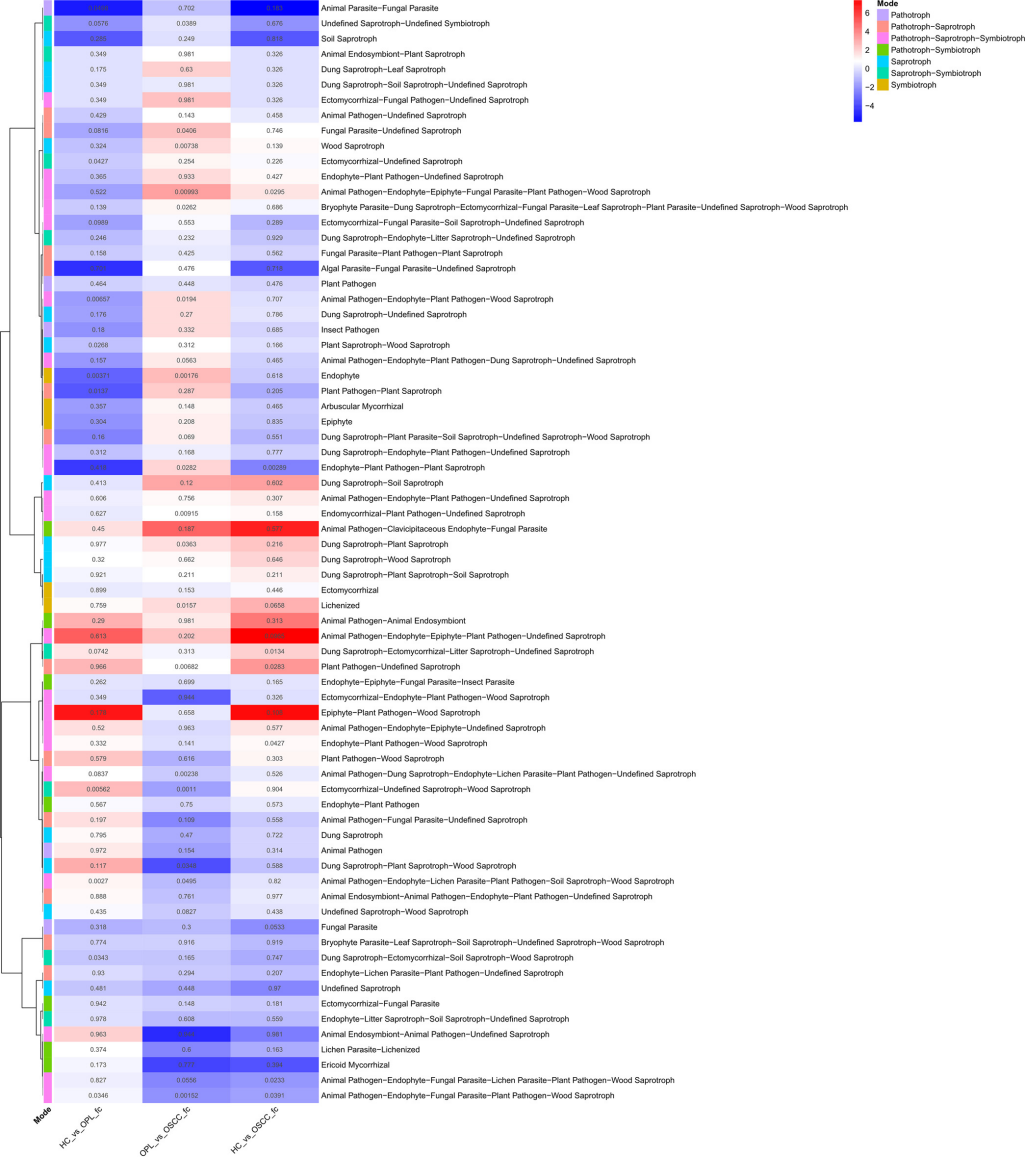
Functional alterations in the buccal mucosal mycobiome. The relative abundances of functional pathways were compared among HC, OPL, and OSCC individuals. Differentially abundant pathways were plotted, and the exact *P* values are presented in the heat map. A generalized fold change of >0 means enriched in the latter; a generalized fold change of <0 means enriched in the former.

Functional prediction analyses indicated functional alterations in bacteria and fungi in multiple different disease states. Analysis of the bacteriome in buccal samples showed that during the development of oral cancer, the pathways associated with carbohydrate biosynthesis, gluconeogenesis, cofactor, prosthetic group, electron carrier, and vitamin biosynthesis were enriched, whereas the pathways associated with carbohydrate degradation, amino acid degradation, amino acids biosynthesis (including branched-chain amino acids), nucleoside, and nucleotide biosynthesis were decreased (Fig. 9). Because of the lack of fungal genomic data, a classification prediction called “FUNGuild” was performed based on data from published articles to predict the function of fungi. The mycobiome associated with the pathways of pathotroph-saprotroph, pathotroph-saprotroph-symbiotroph, and saprotroph was enriched, whereas that associated with the symbiotroph pathway was decreased (Fig. 10). Similar results were found in the plaque swab and saliva samples (Fig. S17 to S20).

## DISCUSSION

The present study is the first to reveal that as OSCC progresses along the health-premalignancy-carcinoma sequence, oral microbial communities can establish dynamic microecosystems, giving rise to specific bacterial and fungal populations and functional features that potentially promote tumorigenesis. A unique feature of our experimental design was the simultaneous sampling of the buccal and gingival plaque swabs and saliva from individuals at different stages of oral carcinogenesis. Our results revealed changes in the oral niches across the stages of oral carcinogenesis, which manifested as distinct microbial dysbiosis. Long-term dysbiosis can lead to alterations in bacterial and fungal genes and their metabolic pathways, which can induce a variety of human diseases, including cancer (16, 17).

It has been reported that a distinct microbiome, called the “core microbiome,” may be used as a biomarker to predict tumorigenesis (14, 18–20). For example, previous research indicated an important role of *Fusobacterium* and *Capnocytophaga*, two of the oral bacterial OTUs also found in this study, in the development of OSCC (21, 22). In addition, this study showed that Prevotella intermedia was significantly enriched and distinct in all three OSCC sample types, which is consistent with the results of previous studies (23). P. intermedia, which carries and expresses interpain A, is a main cause of periodontitis (24). Previously, several models of periodontal bacterial signatures linked to the pathology of several systemic diseases, including cancer, were established (25, 26). Here, significant bacterial alterations in the oral microbiota were shown to be closely related to periodontitis-associated genera. *Prevotella*, *Capnocytophaga*, *Aggregatibacter*, *Fusobacterium*, and *Alloprevotella* were found to be persistently enriched across the entire process of OSCC development. These results suggest that periodontitis-associated pathogens play a critical role in OSCC progression. In contrast, results showed a sharp decrease in the common health-associated commensal bacteriome genera Streptococcus and *Veillonella*, which have been shown to work against the development of oral mucosal lesions (27). The increase in harmful bacteria and the depletion of beneficial bacteria lead to oral microbial dysbiosis and may promote tumor development.

To reveal full-scale associations between microbes and oral tumor status, we also analyzed the variation in fungi. As with bacteria, results showed that harmful fungi were increased during OSCC progression, during which beneficial fungi were depleted. *Candida*, a well-known oral pathogen, was consistently enriched in OPL and OSCC patients, similar to reports from previous studies (28, 29). Moreover, we found that *Acremonium* and Aspergillus were significantly increased during OSCC progression. *Acremonium* and Aspergillus also have been shown to be harmful to health (30, 31). In contrast, *Morchella*, which has been proven to exert a strong inhibitory activity against harmful bacteria (32, 33), was greatly decreased in premalignant and malignant individuals. Polysaccharide FMP-1 from *Morchella esculenta* can exert significant antioxidant activity, endowing it with prebiotic effects and antitumor activity (34). The sharp decline in *Morchella* may indicate the formation of oral harmful niches and the increased possibility of oral disease. This finding provides a potential novel approach for the prevention and treatment of OSCC. As with the “oncogenic bacteria,” variations were found in the abundance of certain bacterial species that promote tumorigenesis (35, 36), as well as significant alterations in fungal diversity and relative abundance at the species level of *Acremonium exuviarum*, Aspergillus fumigatus, and Candida tropicalis, with these identified as “oncogenic fungi.” Acrebol, a mycotoxin produced by an *A*. *exuviarum* indoor isolate, has a unique mitochondrial toxicity and may be hazardous to health (37). A. fumigatus, an opportunistic fungus that causes potentially lethal invasive infections in immunocompromised individuals (38), was significantly enriched in OSCC compared with HC. It was associated with the activation of the nuclear factor-κB (NF-κB) and strongly induced reactive oxygen species (ROS) production (39), which promotes oral carcinogenesis (40, 41). As a sister species of C. albicans, C. tropicalis shares similar pathogenic traits and can cause superficial infections in locations such as the oral mucosa and genital tract (42, 43). Moreover, *in vitro* studies have demonstrated that C. tropicalis and bacteria such as Escherichia coli and Serratia marcescens cooperate to form robust biofilms (44). Biofilms render the organisms resistant to antimicrobial agents and protect them from immune cells (45). Further study has shown that the presence of a bacterial biofilm may contribute to development of cancer (46).

Our findings suggest that the bacteriome or mycobiome may not play a role in OSCC tumorigenesis alone. Pioneering studies have performed analyses focusing on the role of the interplay between the bacteriome and mycobiome in the occurrence and development of human diseases (14, 28, 44, 47). Fungal and bacterial populations coexist in the oral cavity, frequently forming mixed-species biofilms, which is associated with increased antimicrobial resistance. Importantly, dual-species biofilms have shown increased resistance to drug treatment compared to that of single-species biofilms (48). Moreover, Montelongo-Jauregui et al. (49) identified a high degree of complexity in the interactions between Candida albicans and Streptococcus gordonii in mixed-species biofilms, which may impact homeostasis in the oral cavity. In our study, we also found that Streptococcus was positively correlated with *Candida* in the buccal swab samples of the OSCC group. These findings unraveled that specific interkingdom microbial interactions may be important determinants in OSCC. However, fungus-bacterium interactions were found to be complex and substantially important, communicating through signaling molecules and producing metabolites and toxins that can modulate the immune response or alter the efficacy of treatment (50, 51). Our results on intra- and interkingdom interactions underscore microbial dysbiosis during oral tumorigenesis and provide potential insights into the novel pathogenic mechanisms of OSCC.

Functional prediction analyses are helpful in revealing complex potential mechanisms and improve our understanding and interpretation of OSCC carcinogenesis. In bacteria, d-mannose metabolism was significantly enhanced and branched-chain amino acid (valine and isoleucine) and l-arginine metabolisms were markedly decreased during OSCC oncogenesis. A higher level of d-mannose has been shown to increase the risk of tumorigenesis (52). In human and mouse cells, d-mannose is a potent trigger of the activation of transforming grown factor beta (TGF-β) signaling, which has been shown to promote tumorigenesis (53) and may be critical for stimulating Treg cell differentiation (54). The increased abundance pattern of the d-mannose biosynthesis pathway from health to OPL to OSCC suggests that the elevated activity of this pathway may be an important factor in inducing the sustained aggravation of TGF-β signaling during the development of OSCC. More recently, alterations in branched-chain amino acid metabolism have been reported to contribute to cancer (55). Notably, a decrease in branched-chain amino acid metabolism was also found in patients with colorectal cancer (56), suggesting that inhibition of branched-chain amino acids may be critical to oncogenic activities. In fungi, our results showed that they also play a role in metabolism during OSCC progression; however, more studies are needed to reveal the specific mechanism.

Our research has confirmed that the oral microbiome differs between individual OSCC patients. Some studies have utilized saliva samples (26, 28, 57, 58), whereas others analyzed mucosal samples (18, 20). Oral mucosal surfaces have neutral pH, whereas gingival plaques have alkaline pH (59). Mucus containing glycans from saliva favors the growth of bacteria. The human oral cavity harbors complex and diverse biofilms of different niches and the second-most-abundant microbiota after the gastrointestinal tract (60). The fecal microbiome is not representative of the mucosal microbiome (61). Similarly, the salivary microbiome is not representative of the oral mucosal microbiome. Microbiome comparisons are often made between normal and tumor tissue. Few studies, however, have specifically investigated the oral microbiome during the premalignancy-carcinoma sequence. This distinction can be made by specifying each sample’s genetic and epigenetic characteristics or location in the mouth as a proxy. In fact, previous studies (62, 63) showed that the oral microecosystem is highly heterogeneous, containing distinct niches with significantly different microbial communities. For these reasons, we simultaneously sampled a buccal swab, the gingival plaque, and the saliva of individuals at different stages of oral carcinogenesis and found significant differences among these samples, stages, and individuals. Our findings indicate the urgent need to define the core oral microbiome based on oral niches.

This study provides a repository of data on bacterial and fungal species that warrant functional validation to assess their diagnostic value in oral tumorigenesis. Detection of a lower number of fungal reads in our samples also indicates the need to develop a comprehensive reference sequence database of fungi associated with oral carcinogenesis. Although a pioneering study has investigated the spatial microbiome based on oral, gut, cutaneous, vaginal, and other samples (64), it is still in its infancy. Although we studied the bacteriome and mycobiome of buccal mucosa, supragingival plaque, and saliva, it is a limitation that we did not sample from other sites, including the tongue mucosa and subgingival plaque. Therefore, further studies involving more oral niches are needed to develop health- and disease-related spatial microbiology.

### Conclusions.

Taken together, these data revealed dynamic shifts in the oral microbial composition and defined the core microbiome with distinct niches during OSCC progression. A combination of the bacteriome and mycobiome may play a role in OSCC carcinogenesis. Furthermore, it will be essential to identify the potential role of the oral microbiome in human health and disease by investigating the cross talk between the microbial community and immunocyte phenotypes in future prospective studies. An array of signature species that are enriched or depleted in OSCC may be used as biomarkers to predict oral carcinogenesis, contributing to the development of early diagnostic assays and novel treatments using probiotics or prebiotics.

## MATERIALS AND METHODS

### Study group.

Healthy controls (HC, *n* = 30), patients affected with oral premalignant lesions (OPL, *n* = 32), and OSCC patients (*n* =29) were enrolled in this study (see Table S1 in the supplemental material). Buccal swabs, plaque swabs, and saliva samples were collected from HC and patients with OPL and OSCC at the Nanjing Stomatological Hospital, Medical School of Nanjing University, with informed consent. This study protocol was in agreement with the World Medical Association Declaration of Helsinki (2008) and the Belmont Report. Patients with dentition loss, edentulous jaw, and severe periodontal disease with a history of colorectal cancer or other tumors were excluded from the study. Demographic information was obtained (Table S1), and an oral examination was performed. All subjects were classified into three groups: (i) healthy subjects (HC), i.e., individuals without any diagnosed diseases in the oral cavity; (ii) patients with OPL, i.e., individuals with OPL; and (iii) patients with OSCC, i.e., individuals with newly diagnosed OSCC.

### Sample collection and site selection.

After morning awakening, all subjects were asked to avoid rinsing their mouth, tooth brushing, eating, and drinking before sample collection. We first collected buccal samples with a swab by rubbing the buccal mucosa of controls and buccal lesions of the OPL and OSCC patients. Then, we collected supragingival plaque samples from the maxillary and mandibular anterior teeth. Sites selected for sampling were gently dried with sterile cotton rolls, and sterile curettes were used to collect supragingival plaque by scraping for visual confirmation of plaque collection (65, 66). Whole unstimulated saliva (3 mL) was harvested into sterile tubes using Periopaper bands (Orflow, Plainview, NY, USA). A total of 273 samples from 91 subjects (including 29 OSCC patients, 32 OPL patients, and 30 HC subjects) were initially collected. All subjects had sufficient samples for both bacteriome and mycobiome analyses. All samples were stored at −80°C before DNA extraction and sequencing. The methods were performed in accordance with approved guidelines.

### DNA extraction and PCR amplification.

DNA was extracted from swab and saliva samples using a HiPure tissue and blood DNA kit (Magen Biotechnology Co. Ltd., Guangzhou, China). The V3-V4 region of the 16S rRNA gene was amplified using primers 341F (CCTACGGGNGGCWGCAG) and 806R (GGACTACHVGGGTWTCTAAT), and the ITS1 region was amplified using primers F (CTTGGTCATTTAGAGGAAGTAA) and R (GCTGCGTTCTTCATCGATGC). The barcode was a six-base sequence unique to each sample. PCRs were performed in a 30-μL mixture containing 15 μL of 2× Phanta master mix, 1 μL of each primer (10 μM), and 20 ng of DNA template. Amplicons were extracted from 2% agarose gel slices with the desired DNA bands and purified using an AxyPrep DNA gel extraction kit (Axygen Biosciences, Union City, CA, USA). The PCR amplifications for 16S rRNA were performed as follows: 95°C for 5 min, followed by 27 cycles of 95°C for 30 s, 55°C for 30 s, and 72°C for 45 s, and a final extension of 72°C for 10 min, and then 10°C until halted by user. The PCR amplifications for ITS1 were performed as follows: 95°C for 5 min, followed by 29 cycles of 95°C for 30 s, 55°C for 30 s, and 72°C for 45 s, and a final extension of 72°C for 10 min, and then 10°C until halted by user.

### Library construction and sequencing.

Purified PCR amplicons were quantified using a Qubit 3.0 fluorometer (Invitrogen, Carlsbad, CA, USA), and every 20 amplicons whose barcodes were different were mixed equally. Each pooled DNA product was used to construct an Illumina paired-end library in accordance with Illumina’s genomic DNA library preparation procedure (67). Then, the amplicon library was paired-end sequenced (2 × 250) on an Illumina NovaSeq 6000 platform (GenePioneer Co. Ltd, Nanjing, China) according to standard protocols.

### Sequence-based microbial analyses.

Paired-end Illumina reads were assembled using PANDAseq (68). The low-quality raw sequences with an average quality score of <20 bp and truncated reads with an *N* length of 5% of the total sequence length were discarded by PRINSEQ (69). *De novo* OTUs were clustered with 97% sequence similarity, and chimeras were detected with the reference database by VSEARCH (version 2.15.1). To classify each OTU species with a confidence interval of 90%, UCLUST (70) in QIIME (version 1.9.1) (71) was used to compare the OTU sequence representing the known species with the sequence in the SILVA database of 16S or the UNITE database of ITS.

### Bioinformatics analyses.

The rarefaction analysis based on QIIME was conducted to reveal the Shannon and Simpson diversity indices. Principal-component analysis (PCA) was conducted according to genus abundance in microbial samples. The relative abundance of the top 20 genera among the three groups was shown using ternary phase diagrams for the three different sample sources (72). The bubble plots show the top 20 genera in relative abundance among the three groups. In addition, heat maps were generated to reveal the relationship between the bacteriome and mycobiome. The linear discriminant analysis (LDA) effect size (LEfSe) method (73) was used to analyze the differences in bacterial communities between the groups. In this group, species with an LDA score of >3 were the species with a statistical difference between groups. The accuracy of microbiome characterization in predicting the status of oral disease was analyzed using receiver operating characteristic (ROC) curves using the SKLearn package in Python (74). The relative abundances of functional groups (guilds) were compared between HC and OPL or OSCC (75). *P* values presented in the heat map were computed using a two-sided blocked Wilcoxon rank sum test. The color gradients of the heat map indicate log_2_ fold change. For functional analysis, PICRUSt2 (version 2.1.2) was performed to predict the functional characteristics of the bacteriome (76, 77) and FUNGuild (version 1.1) was used to taxonomically parse fungal OTUs into functional groups and trophic modes (78).

### Statistical analyses.

Statistical analysis was performed using R software (version 3.6.2) (R Foundation for Statistical Computing, Vienna, Austria). The differential abundance of bacterial taxa at different levels (phylum, class, order, family, and genus) between the groups was calculated using Metastats software (version 2.0). Metastats analysis was used to detect species with significant differences in abundance between different groups. The differences in alpha diversity indices were determined using the Wilcoxon rank sum test (*P* < 0.05).

### Ethical approval and consent to participate.

This study was approved by the Ethics Committee of Nanjing Stomatological Hospital, Medical School of Nanjing University (Institutional Review Board [IRB] protocol number 2018NL-008 [KS]).

### Data availability.

The data sets presented in this study can be found in online repositories. The name of the repository and the accession numbers are as follows: NCBI SRA accession no. PRJNA788378 for the oral bacteriome and NCBI SRA accession no. PRJNA777754 for the oral mycobiome. The supplemental figures can be downloaded at https://doi.org/10.6084/m9.figshare.21316488.v1, and the supplemental tables can be downloaded at https://doi.org/10.6084/m9.figshare.21316572.v1. All relevant data are available from the corresponding authors upon any reasonable request.
