# Supplementary material for: Oral Bacteriome and Mycobiome across Stages of Oral Carcinogenesis
Source: Microbiol Spectr. 2022 Nov 29;10(6):e02737-22. doi: 10.1128/spectrum.02737-22 (PMC9769585; doi:10.1128/spectrum.02737-22)
Supplement: Supplemental file 1 — Fig. S1 to S20 and Table S1. Download spectrum.02737-22-s0001.pdf, PDF file, 1.7 MB [file spectrum.02737-22-s0001.pdf]

Supplementary Information

The oral bacteriome and mycobiome across stages of oral carcinogenesis

Weiwei Heng†, Wenmei Wang†, Tingting Dai†, Ping Jiang, Yong Lu, Ruowei Li, Miaomiao Zhang, Ruiqi Xie, Yifan Zhou, Maomao Zhao, Ning Duan, Zhengqin Ye*, Fuhua Yan*, Xiang Wang*.

Table of Contents

Supplementary Figure 1. Principal component analysis of the plaque bacteriome from HC, OPL, and OSCC individuals.
Supplementary Figure 2. Principal component analysis of the saliva bacteriome from HC, OPL, and OSCC individuals.
Supplementary Figure 3. Principal component analysis of the plaque mycobiome from HC, OPL, and OSCC individuals.
Supplementary Figure 4. Principal component analysis of the saliva mycobiome from HC, OPL, and OSCC individuals.
Supplementary Figure 5. Bubble plot of the plaque bacteriome and mycobiome from the HC, OPL, and OSCC individuals.
Supplementary Figure 6. Bubble plot of the saliva bacteriome and mycobiome from the HC, OPL, and OSCC individuals.
Supplementary Figure 7. Oral carcinogenesis-associated alterations in the abundance of the plaque bacteria examined using LEfSe.
Supplementary Figure 8. Oral carcinogenesis-associated alterations in the abundance of the saliva bacteria examined using LEfSe.
Supplementary Figure 9. Oral carcinogenesis-associated alterations in the abundance of the plaque fungi examined using LEfSe.
Supplementary Figure 10. Oral carcinogenesis-associated alterations in the abundance of the saliva fungi examined using LEfSe.
Supplementary Figure 11. Intra-kingdom correlation at the genera level in buccal mucosal samples.
Supplementary Figure 12. Intra-kingdom correlation at the genera level in plaque samples.
Supplementary Figure 13. Intra-kingdom correlation at the genera level in saliva samples.
Supplementary Figure 14. Inter-kingdom correlation at the genera level in buccal mucosal samples.
Supplementary Figure 15. Inter-kingdom correlation at the genera level in plaque samples.
Supplementary Figure 16. Inter-kingdom correlation at the genera level in saliva samples.
Supplementary Figure 17. Functional alterations in the plaque bacteriome.
Supplementary Figure 18. Functional alterations in the saliva bacteriome.
Supplementary Figure 19. Functional alterations in the plaque mycobiome.
Supplementary Figure 20. Functional alterations in the saliva mycobiome.

Supplementary Table 1. Baseline information of the HC, OPL and OSCC groups.
Supplementary Table 2. Differentially abundant bacterial phyla of buccal mucosal samples among three groups.
Supplementary Table 3. Differentially abundant bacterial phyla of plaque samples among three groups.
Supplementary Table 4. Differentially abundant bacterial phyla of saliva samples among three groups.
Supplementary Table 5. Differentially abundant fungal phyla of buccal mucosal samples among three groups.
Supplementary Table 6. Differentially abundant fungal phyla of plaque samples among three groups.
Supplementary Table 7. Differentially abundant fungal phyla of saliva samples among three groups.
Supplementary Table 8. Differentially abundant bacterial genera of buccal mucosal samples among three groups.
Supplementary Table 9. Differentially abundant bacterial genera of plaque samples among three groups.
Supplementary Table 10. Differentially abundant bacterial genera of saliva samples among three groups.
Supplementary Table 11. Differentially abundant fungal genera of buccal mucosal samples among three groups.
Supplementary Table 12. Differentially abundant fungal genera of plaque samples among three groups.
Supplementary Table 13. Differentially abundant fungal genera of saliva samples among three groups.

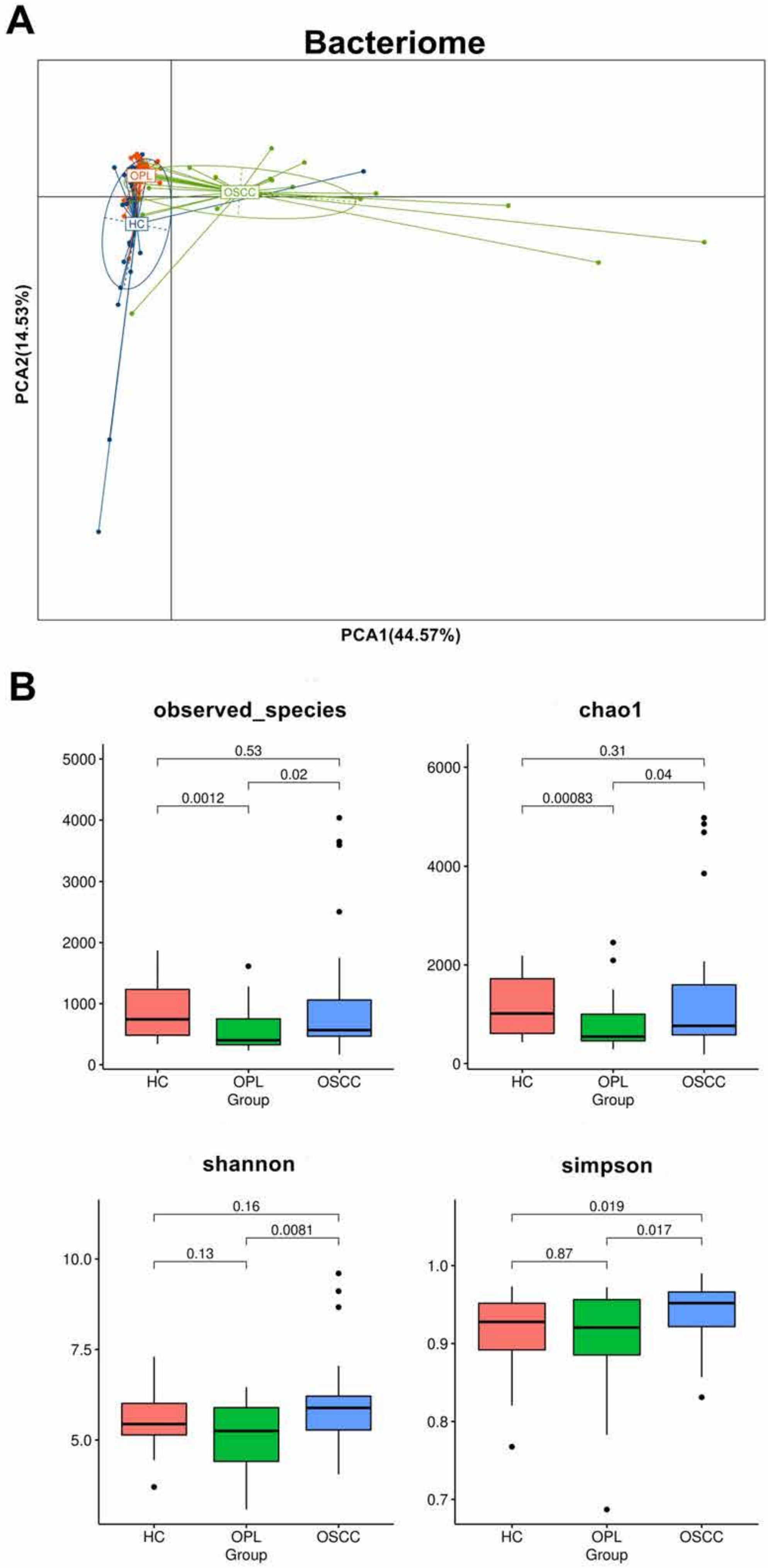
A
Bacteriome
PCA2(14.53%)
PCA1(44.57%)
OPL
OSCC
HC
B
observed_species
chao1
shannon
simpson
0.53
0.02
0.0012
0.31
0.04
0.00083
0.16
0.0081
0.13
0.019
0.017
0.87
HC
OPL
OSCC
Group

Supplementary Figure 1. Principal component analysis of the plaque bacteriome from HC, OPL, and OSCC individuals. (A) The plaque bacteriome of individuals with OSCC was statistically significantly different (P < 0.05) from HC and OPL individuals. (B) Box plots show the diversity and richness of the plaque bacteriome from the HP, OPL, and OSCC groups at the OTU level.

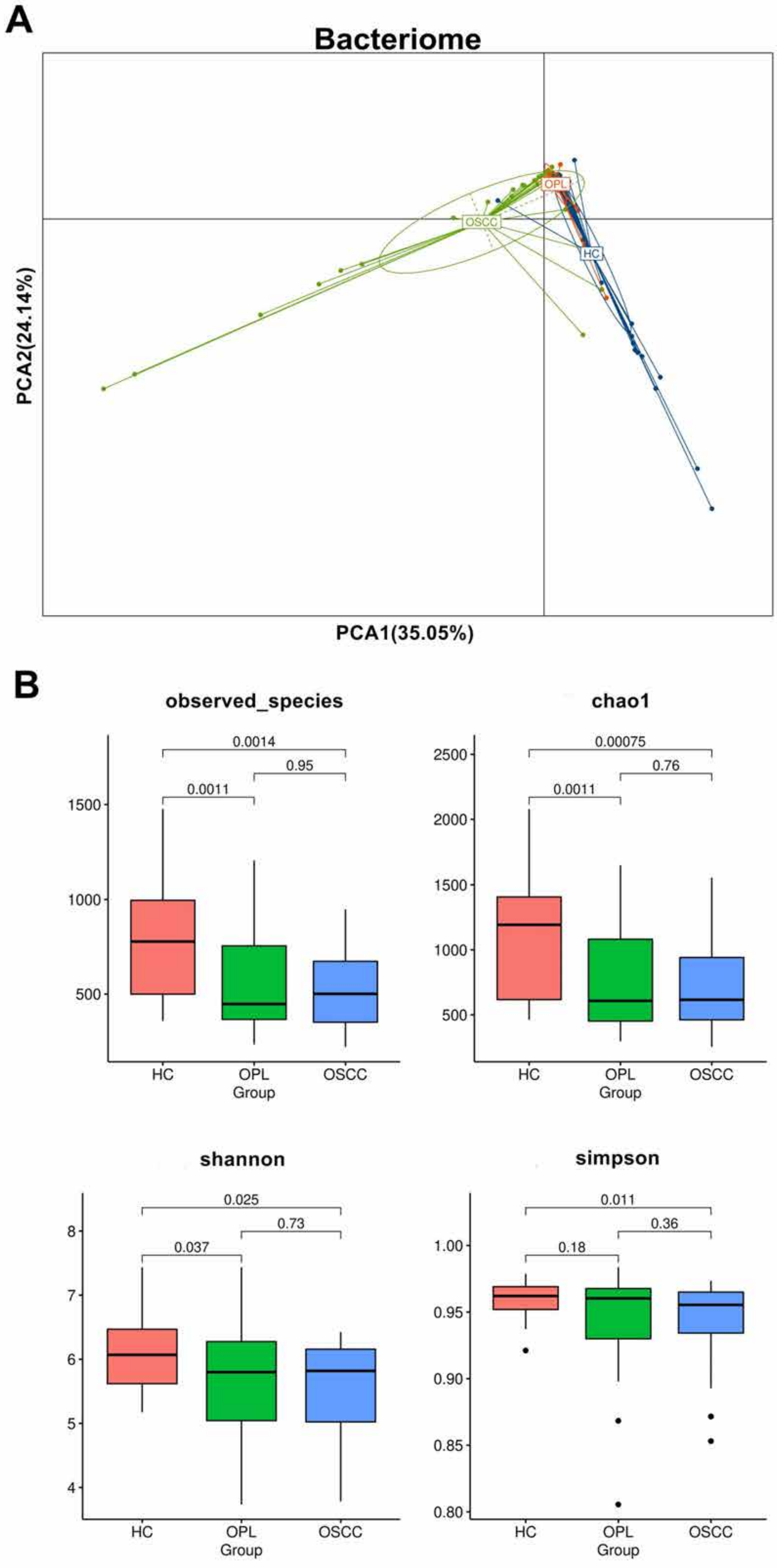
A
Bacteriome
PCA2(24.14%)
PCA1(35.05%)
OPL
OSCC
HC
B
observed_species
chao1
shannon
simpson
0.0014
0.95
0.0011
0.00075
0.76
0.0011
0.025
0.73
0.037
0.011
0.36
0.18
HC
OPL
OSCC
Group

Supplementary Figure 2. Principal component analysis of the saliva bacteriome from HC, OPL, and OSCC individuals. (A) The plaque mycobiome of individuals with OSCC was statistically significantly different ($P < 0.05$) from HC and OPL individuals. (B) Box plots show the diversity and richness of the saliva bacteriome from the HP, OPL, and OSCC groups at the OTU level.

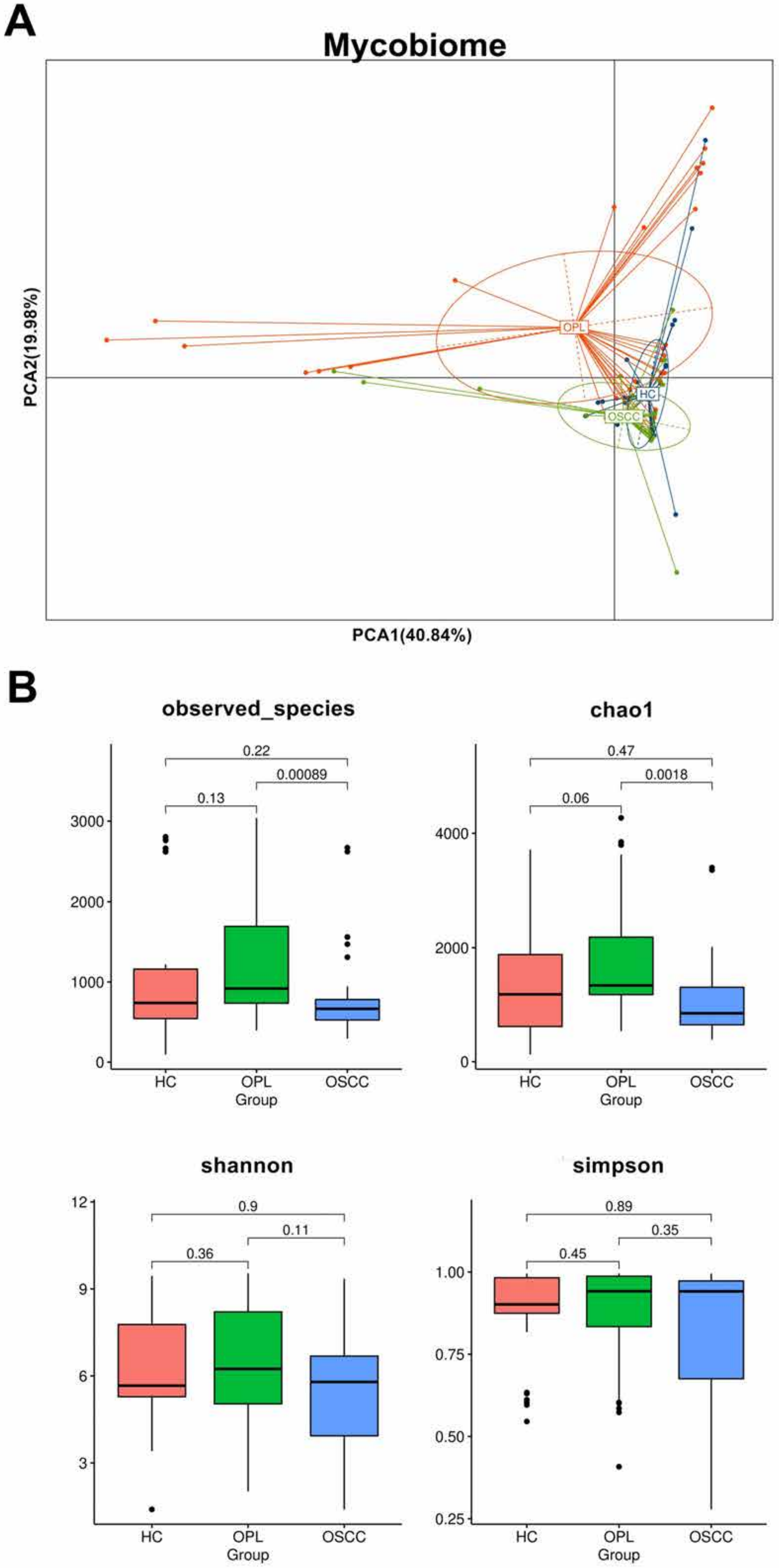

A
Mycobiome
PCA2(19.98%)
PCA1(40.84%)
OPL
HC
OSCC
B
observed_species
0.22
0.00089
0.13
3000
2000
1000
0
HC
OPL
OSCC
Group
chao1
0.47
0.0018
0.06
4000
2000
0
HC
OPL
OSCC
Group
shannon
0.9
0.11
0.36
12
9
6
3
HC
OPL
OSCC
Group
simpson
0.89
0.35
0.45
1.00
0.75
0.50
0.25
HC
OPL
OSCC
Group

Supplementary Figure 3. Principal component analysis of the plaque mycobiome from HC, OPL, and OSCC individuals. (A) The saliva bacteriome of individuals with OSCC was statistically significantly different (P < 0.05) from HC and OPL individuals. (B) Box plots show the diversity and richness of the plaque mycobiome from the HP, OPL, and OSCC groups at the OTU level.

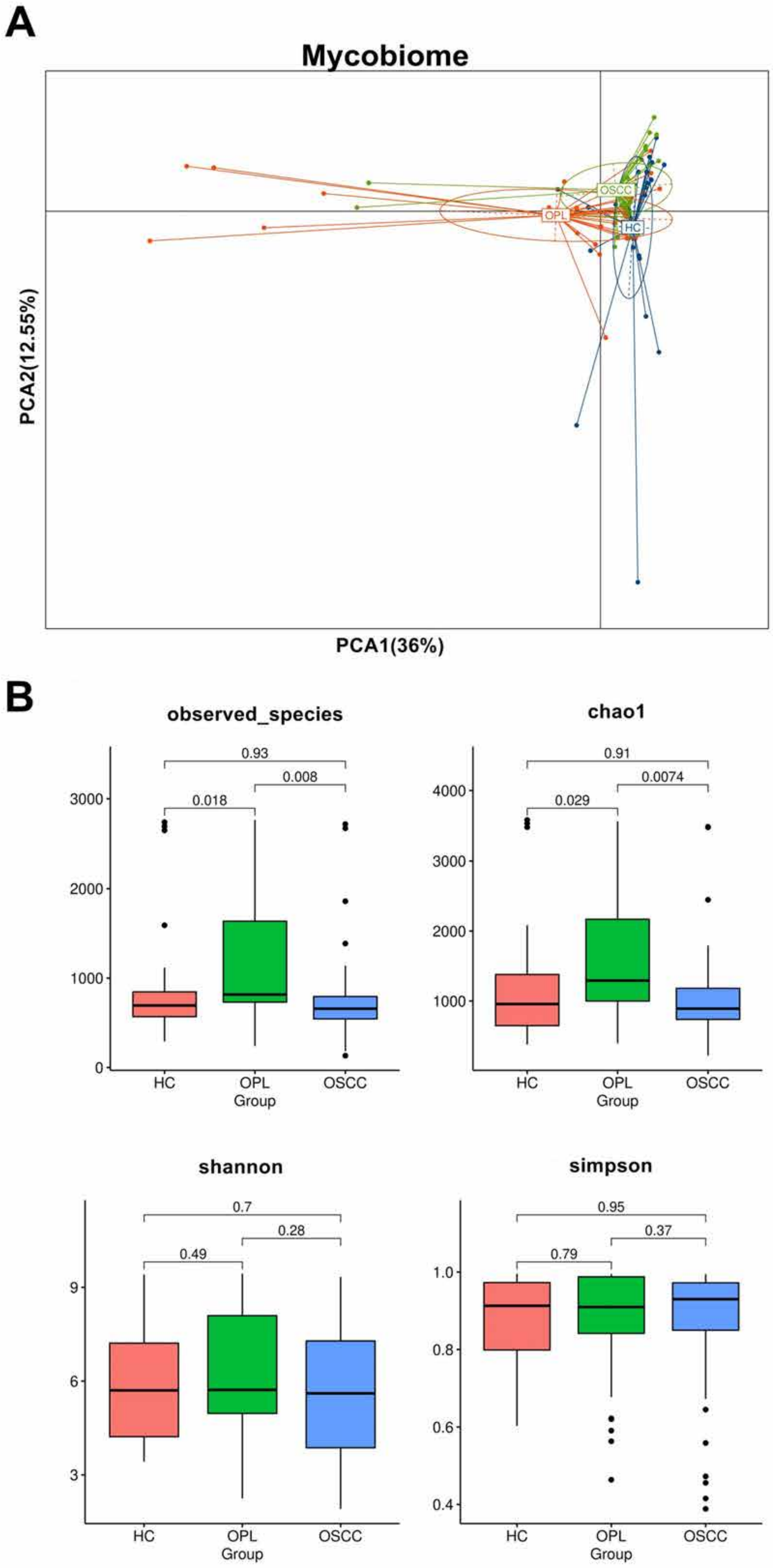

A
Mycobiome
OSCC
OPL
HC
PCA2(12.55%)
PCA1(36%)
B
observed_species
0.93
0.008
0.018
3000
2000
1000
0
HC
OPL
OSCC
Group
chao1
0.91
0.0074
0.029
4000
3000
2000
1000
HC
OPL
OSCC
Group
shannon
0.7
0.28
0.49
9
6
3
HC
OPL
OSCC
Group
simpson
0.95
0.37
0.79
1.0
0.8
0.6
0.4
HC
OPL
OSCC
Group

Supplementary Figure 4. Principal component analysis of the saliva mycobiome from HC, OPL, and OSCC individuals. (A) The saliva mycobiome of individuals with OSCC was statistically significantly different (P < 0.05) from HC and OPL individuals. (B) Box plots show the diversity and richness of the saliva mycobiome from the HP, OPL, and OSCC groups at the OTU level.

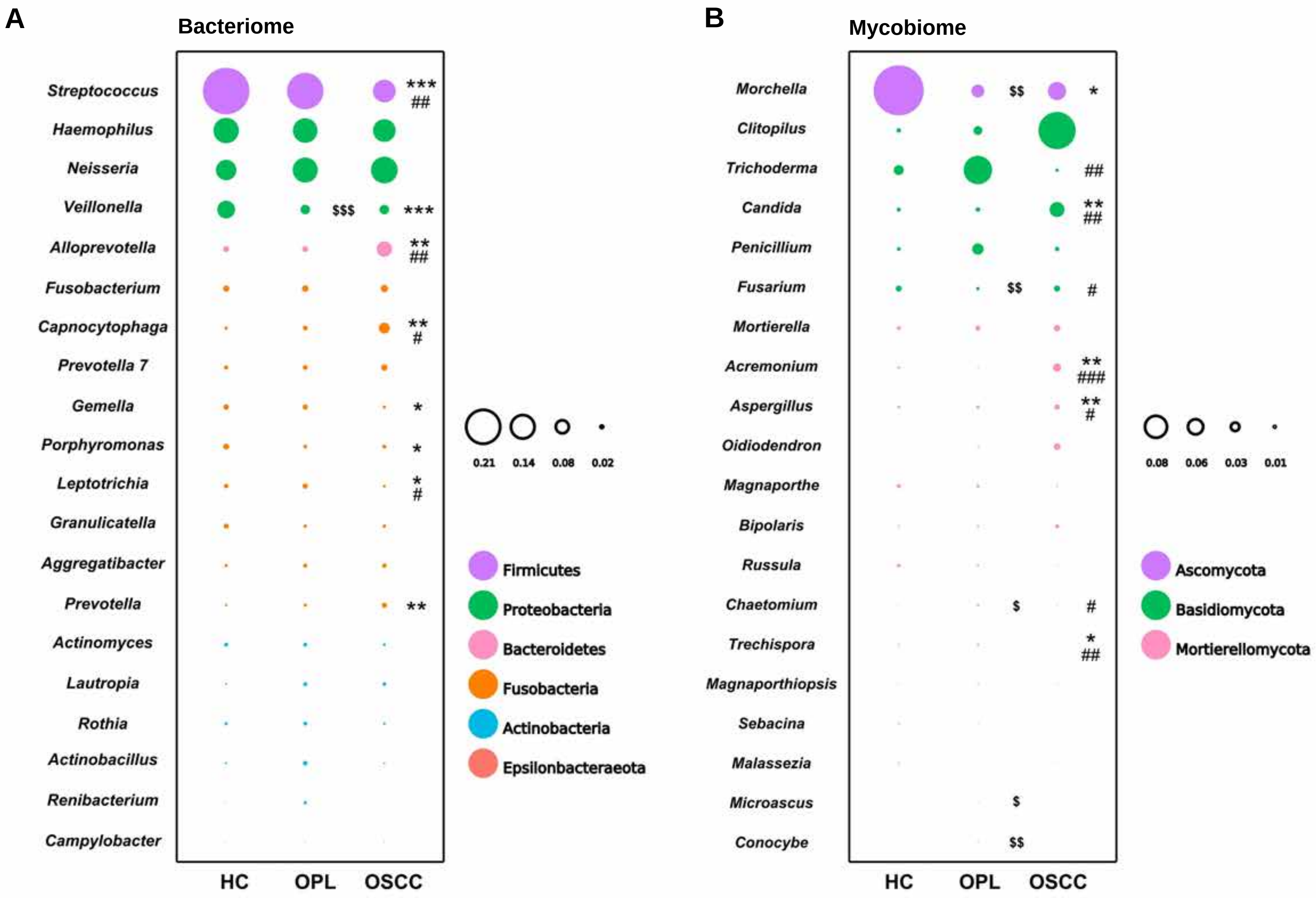
A
Bacteriome
Streptococcus
Haemophilus
Neisseria
Veillonella
Alloprevotella
Fusobacterium
Capnocytophaga
Prevotella 7
Gemella
Porphyromonas
Leptotrichia
Granulicatella
Aggregatibacter
Prevotella
Actinomyces
Lautropia
Rothia
Actinobacillus
Renibacterium
Campylobacter
HC
OPL
OSCC
0.21
0.14
0.08
0.02
Firmicutes
Proteobacteria
Bacteroidetes
Fusobacteria
Actinobacteria
Epsilonbacteraeota
B
Mycobiome
Morchella
Clitopilus
Trichoderma
Candida
Penicillium
Fusarium
Mortierella
Acremonium
Aspergillus
Oidiodendron
Magnaporthe
Bipolaris
Russula
Chaetomium
Trechispora
Magnaporthiopsis
Sebacina
Malassezia
Microascus
Conocybe
HC
OPL
OSCC
0.08
0.06
0.03
0.01
Ascomycota
Basidiomycota
Mortierellomycota

Supplementary Figure 5. Bubble plot of the plaque (A) bacteriome and (B) mycobiome from HC, OPL, and OSCC individuals. Each bubble represents one genus and the bubble size represents the relative abundance of each genus. For the OSCC group, *, ** and *** denote $P < 0.05$, $P < 0.01$ and $P < 0.001$ versus the HC group, respectively. For the OSCC group, #, ## and ### denote $P < 0.05$, $P < 0.01$ and $P < 0.001$ versus the OPL group, respectively. For the OPL group, \$, \$\$ and \$\$\$ denote $P < 0.05$, $P < 0.01$ and $P < 0.001$ versus the HC group, respectively.

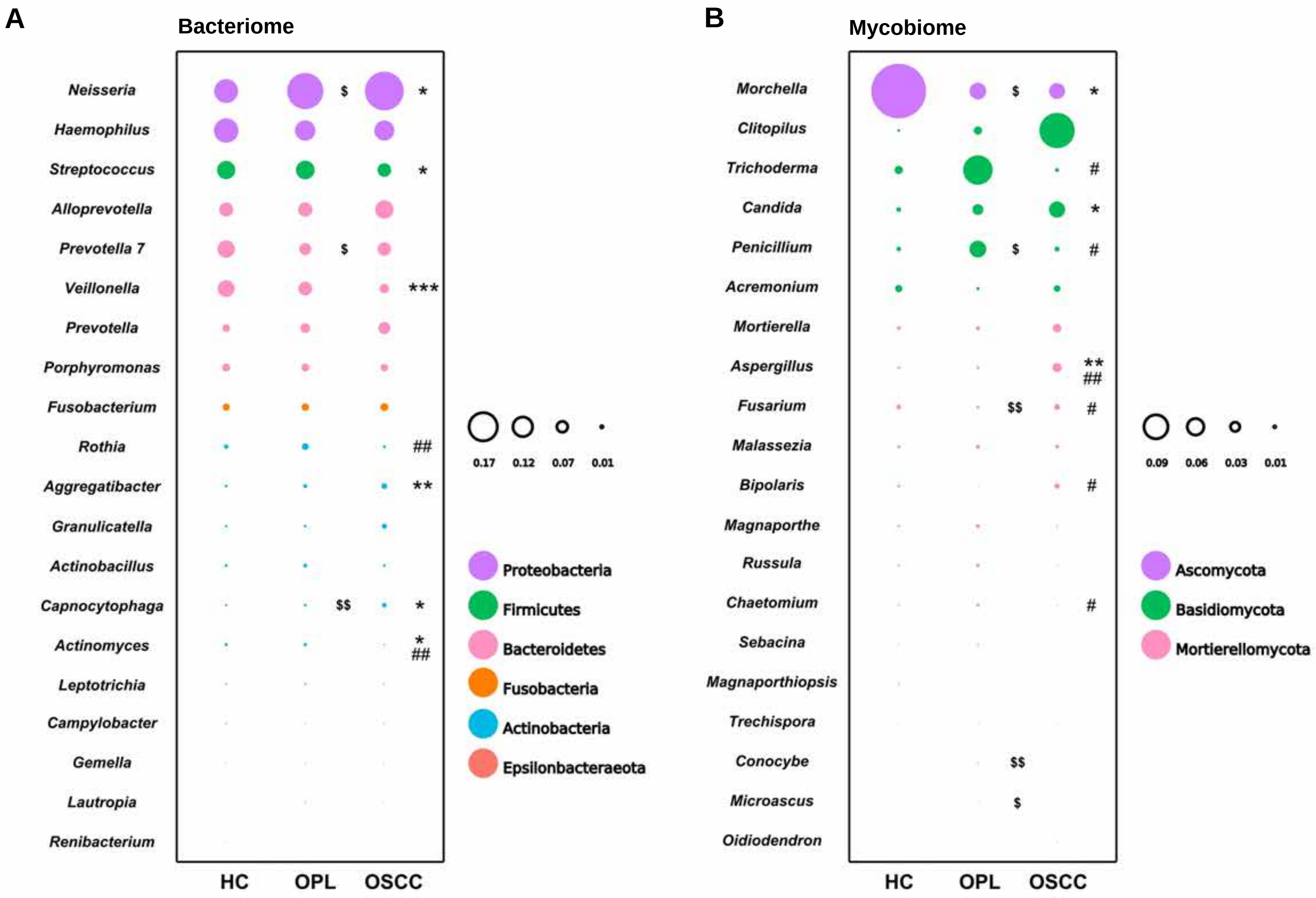
A
Bacteriome
Neisseria
Haemophilus
Streptococcus
Alloprevotella
Prevotella 7
Veillonella
Prevotella
Porphyromonas
Fusobacterium
Rothia
Aggregatibacter
Granulicatella
Actinobacillus
Capnocytophaga
Actinomyces
Leptotrichia
Campylobacter
Gemella
Lautropia
Renibacterium
$
*
*
$
***
##
**
$$
*
*
##
HC
OPL
OSCC
0.17
0.12
0.07
0.01
Proteobacteria
Firmicutes
Bacteroidetes
Fusobacteria
Actinobacteria
Epsilonbacteraeota
B
Mycobiome
Morchella
Clitopilus
Trichoderma
Candida
Penicillium
Acremonium
Mortierella
Aspergillus
Fusarium
Malassezia
Bipolaris
Magnaporthe
Russula
Chaetomium
Sebacina
Magnaporthiopsis
Trechispora
Conocybe
Microascus
Oidiodendron
$
*
#
*
$
#
**
##
$$
#
#
#
$$
$
HC
OPL
OSCC
0.09
0.06
0.03
0.01
Ascomycota
Basidiomycota
Mortierellomycota

Supplementary Figure 6. Bubble plot of the saliva (A) bacteriome and (B) mycobiome from the HC, OPL, and OSCC individuals. Each bubble represents one genus and the bubble size represents the relative abundance of each genus. For the OSCC group, *, ** and *** denote $P < 0.05$, $P < 0.01$ and $P < 0.001$ versus the HC group, respectively. For the OSCC group, # and ## denote $P < 0.05$ and $P < 0.01$ versus the OPL group, respectively. For the OPL group, $ and $$ denote $P < 0.05$ and $P < 0.01$ versus the HC group, respectively.

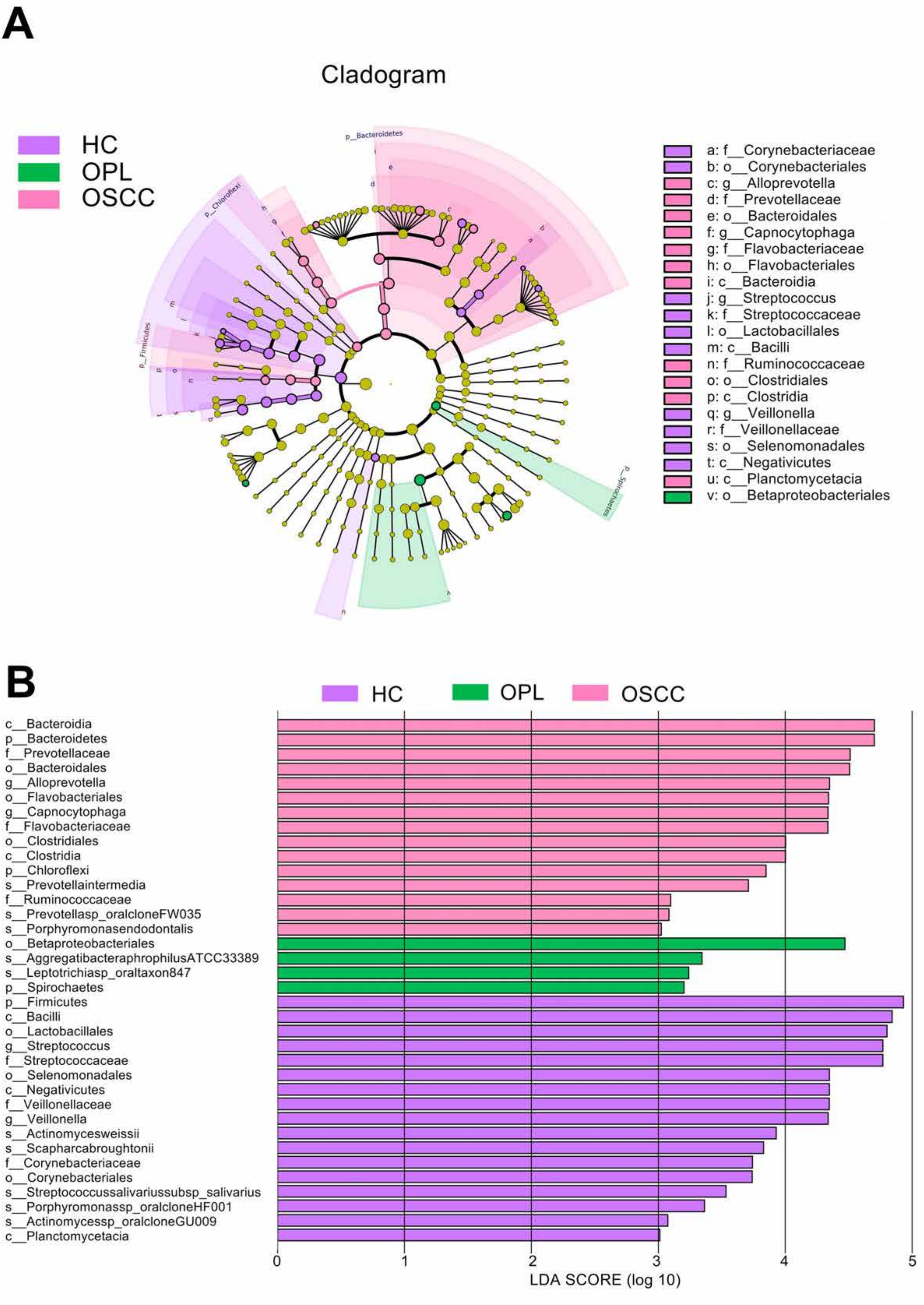
A
Cladogram
HC
OPL
OSCC
p__Bacteroidetes
p__Chloroflexi
p__Firmicutes
p__Spirochaetes
a: f__Corynebacteriaceae
b: o__Corynebacteriales
c: g__Alloprevotella
d: f__Prevotellaceae
e: o__Bacteroidales
f: g__Capnocytophaga
g: f__Flavobacteriaceae
h: o__Flavobacteriales
i: c__Bacteroidia
j: g__Streptococcus
k: f__Streptococcaceae
l: o__Lactobacillales
m: c__Bacilli
n: f__Ruminococcaceae
o: o__Clostridiales
p: c__Clostridia
q: g__Veillonella
r: f__Veillonellaceae
s: o__Selenomonadales
t: c__Negativicutes
u: c__Planctomycetacia
v: o__Betaproteobacteriales
B
HC
OPL
OSCC
c__Bacteroidia
p__Bacteroidetes
f__Prevotellaceae
o__Bacteroidales
g__Alloprevotella
o__Flavobacteriales
g__Capnocytophaga
f__Flavobacteriaceae
o__Clostridiales
c__Clostridia
p__Chloroflexi
s__Prevotellaintermedia
f__Ruminococcaceae
s__Prevotellasp_oralcloneFW035
s__Porphyromonasendodontalis
o__Betaproteobacteriales
s__AggregatibacteraphrophilusATCC33389
s__Leptotrichiasp_oraltaxon847
p__Spirochaetes
p__Firmicutes
c__Bacilli
o__Lactobacillales
g__Streptococcus
f__Streptococcaceae
o__Selenomonadales
c__Negativicutes
f__Veillonellaceae
g__Veillonella
s__Actinomycesweissii
s__Scapharcabroughtonii
f__Corynebacteriaceae
o__Corynebacteriales
s__Streptococcussalivariussubsp_salivarius
s__Porphyromonassp_oralcloneHF001
s__Actinomycessp_oralcloneGU009
c__Planctomycetacia
0
1
2
3
4
5
LDA SCORE (log 10)

Supplementary Figure 7. Oral carcinogenesis-associated alterations in the abundance of the plaque bacteria examined using LEfSe. (A) Cladogram indicates the phylogenetic distribution of active bacteria that were remarkably enriched. (B) Bar plots at the species level with significant differences in abundance based on LEfSe. The color bars show the LDA scores of species that were enriched in the indicated condition: purple bar (HC), green bar (OPL), and pink bar (OSCC).

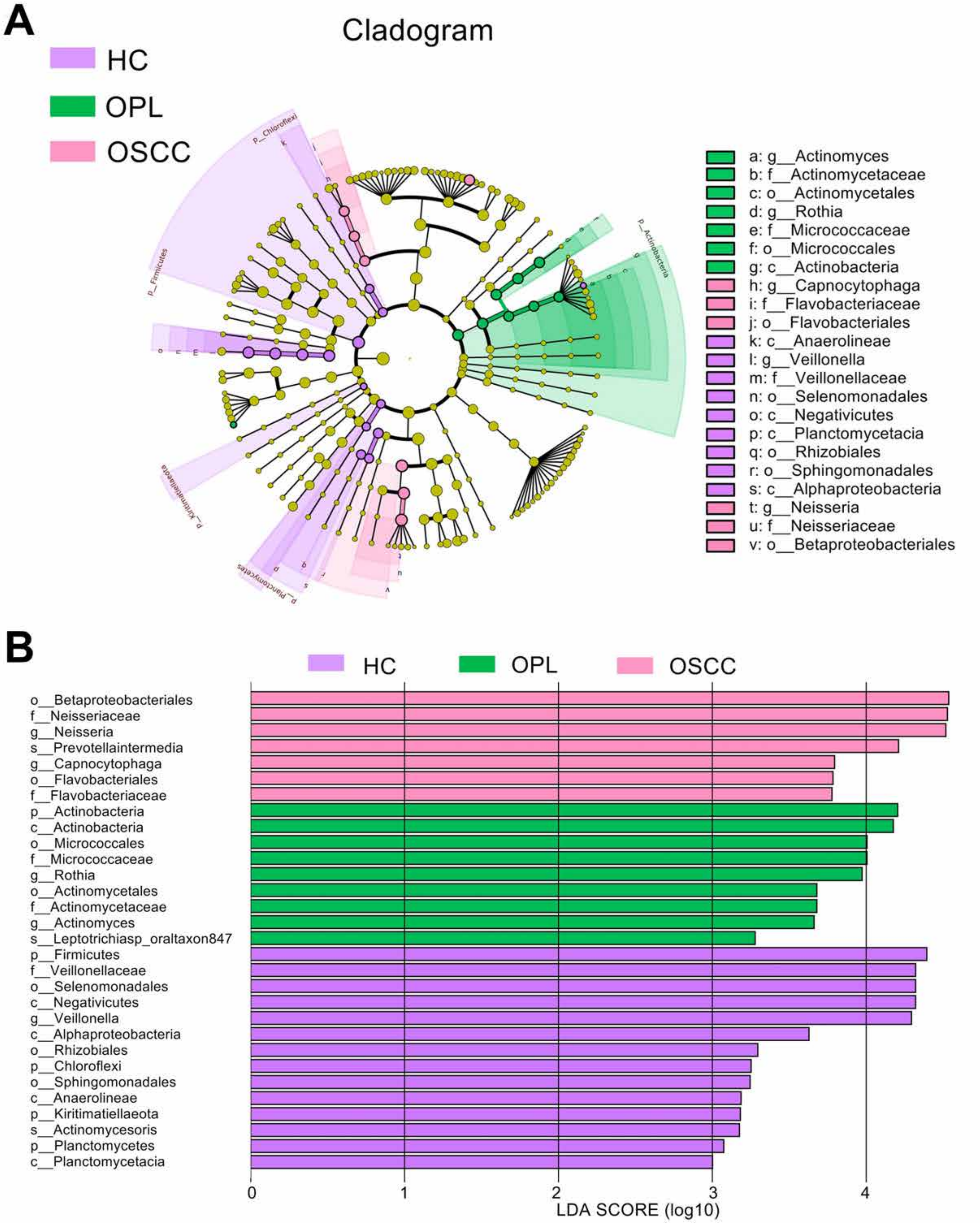

A
Cladogram
HC
OPL
OSCC
p__Chloroflexi
p__Actinobacteria
p__Firmicutes
p__Kiritimatiellaeota
p__Planctomycetes
a: g__Actinomyces
b: f__Actinomycetaceae
c: o__Actinomycetales
d: g__Rothia
e: f__Micrococcaceae
f: o__Micrococcales
g: c__Actinobacteria
h: g__Capnocytophaga
i: f__Flavobacteriaceae
j: o__Flavobacteriales
k: c__Anaerolineae
l: g__Veillonella
m: f__Veillonellaceae
n: o__Selenomonadales
o: c__Negativicutes
p: c__Planctomycetacia
q: o__Rhizobiales
r: o__Sphingomonadales
s: c__Alphaproteobacteria
t: g__Neisseria
u: f__Neisseriaceae
v: o__Betaproteobacteriales
B
HC
OPL
OSCC
o__Betaproteobacteriales
f__Neisseriaceae
g__Neisseria
s__Prevotellaintermedia
g__Capnocytophaga
o__Flavobacteriales
f__Flavobacteriaceae
p__Actinobacteria
c__Actinobacteria
o__Micrococcales
f__Micrococcaceae
g__Rothia
o__Actinomycetales
f__Actinomycetaceae
g__Actinomyces
s__Leptotrichiasp_oraltaxon847
p__Firmicutes
f__Veillonellaceae
o__Selenomonadales
c__Negativicutes
g__Veillonella
c__Alphaproteobacteria
o__Rhizobiales
p__Chloroflexi
o__Sphingomonadales
c__Anaerolineae
p__Kiritimatiellaeota
s__Actinomycesoris
p__Planctomycetes
c__Planctomycetacia
0
1
2
3
4
LDA SCORE (log10)

Supplementary Figure 8. Oral carcinogenesis-associated alterations in the abundance of the saliva bacteria examined using LEfSe. (A) Cladogram indicates the phylogenetic distribution of active bacteria that were remarkably enriched. (B) Bar plots at the species level with significant differences in abundance based on LEfSe. The color bars show the LDA scores of species that were enriched in the indicated condition: purple bar (HC), green bar (OPL), and pink bar (OSCC).

A

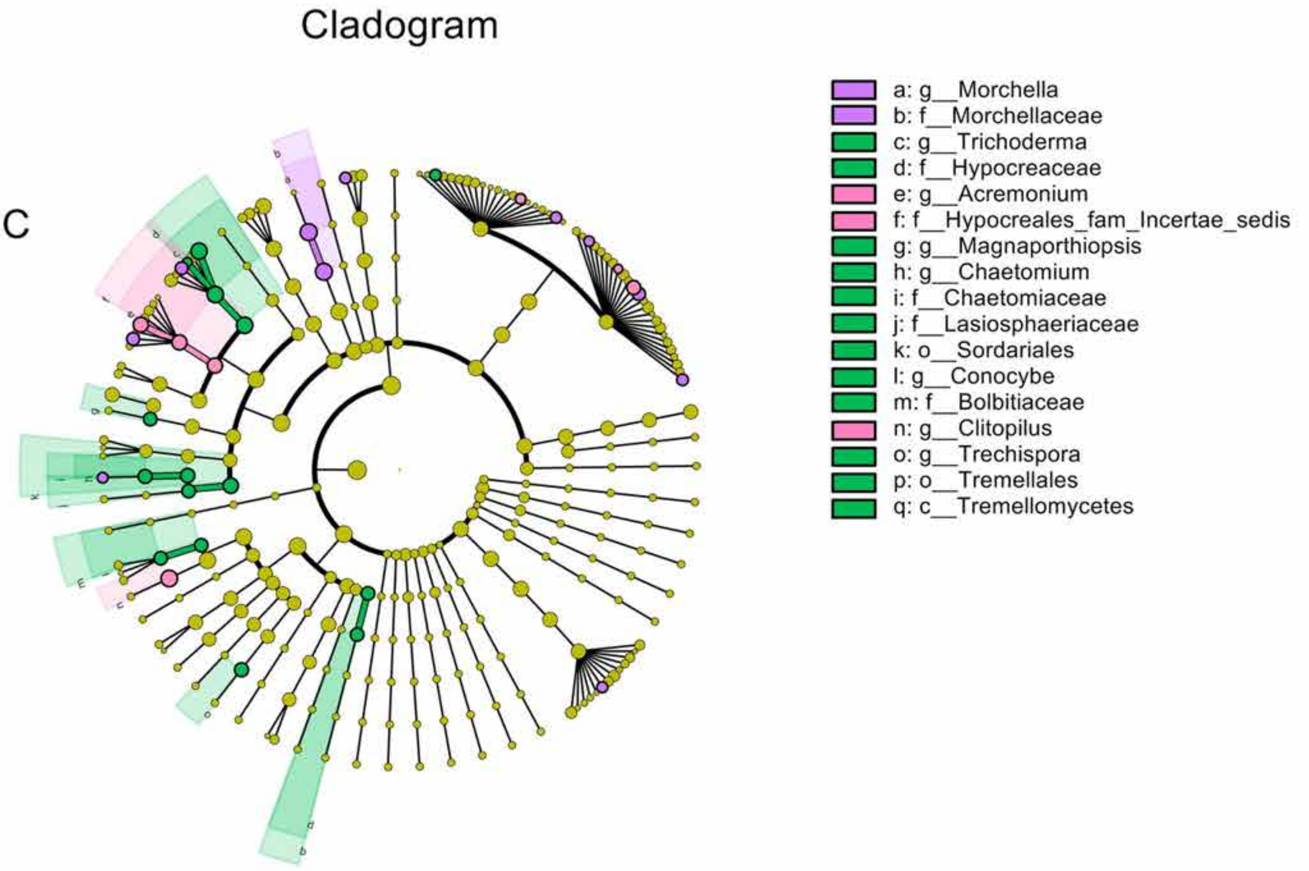
Cladogram
HC
OPL
OSCC
a: g__Morchella
b: f__Morchellaceae
c: g__Trichoderma
d: f__Hypocreaceae
e: g__Acremonium
f: f__Hypocreales_fam_Incertae_sedis
g: g__Magnaporthiopsis
h: g__Chaetomium
i: f__Chaetomiaceae
j: f__Lasiosphaeriaceae
k: o__Sordariales
l: g__Conocybe
m: f__Bolbitiaceae
n: g__Clitopilus
o: g__Trechispora
p: o__Tremellales
q: c__Tremellomycetes

B

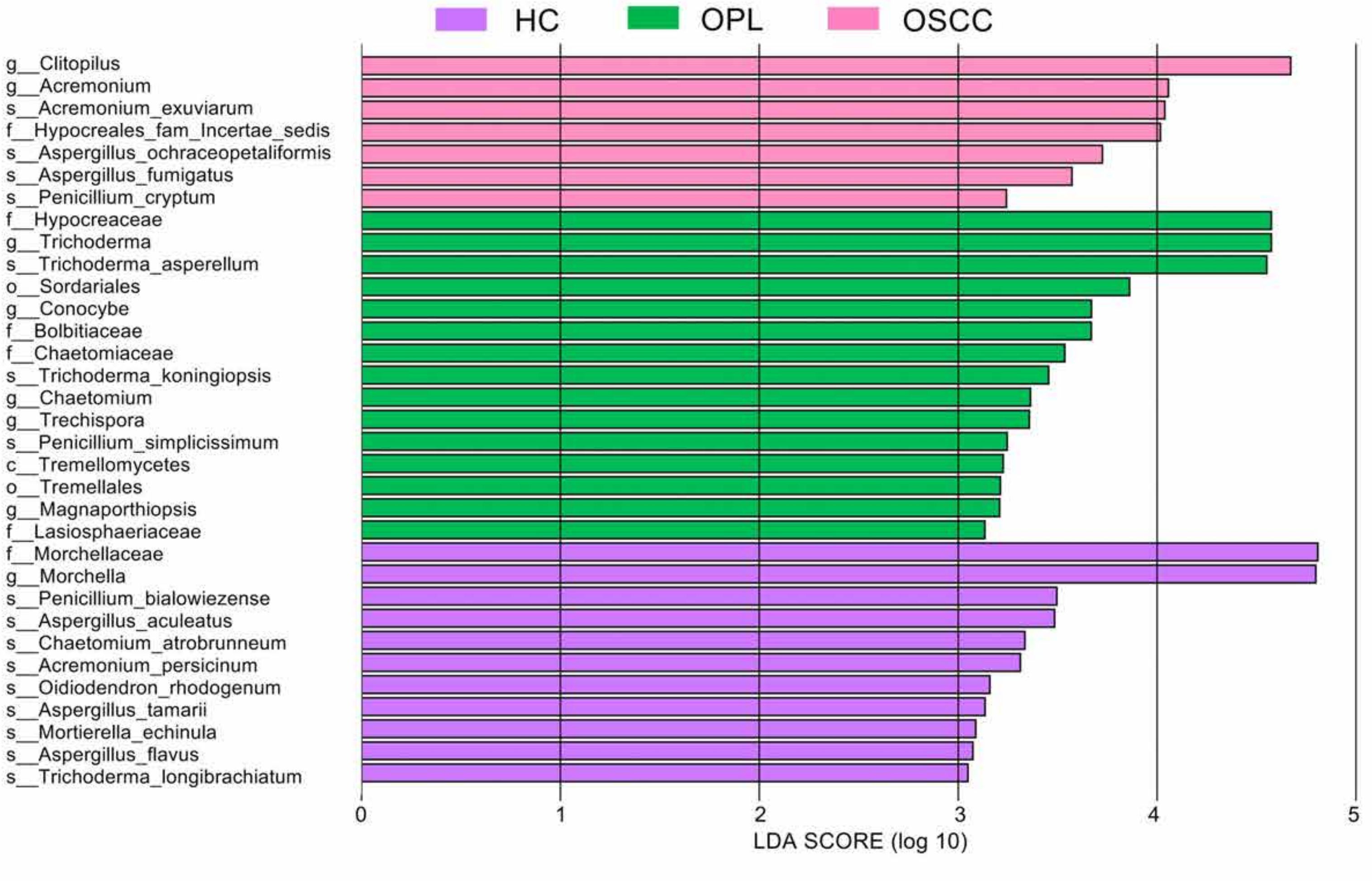
HC
OPL
OSCC
g__Clitopilus
g__Acremonium
s__Acremonium_exuviarum
f__Hypocreales_fam_Incertae_sedis
s__Aspergillus_ochraceopetaliformis
s__Aspergillus_fumigatus
s__Penicillium_cryptum
f__Hypocreaceae
g__Trichoderma
s__Trichoderma_asperellum
o__Sordariales
g__Conocybe
f__Bolbitiaceae
f__Chaetomiaceae
s__Trichoderma_koningiopsis
g__Chaetomium
g__Trechispora
s__Penicillium_simplicissimum
c__Tremellomycetes
o__Tremellales
g__Magnaporthiopsis
f__Lasiosphaeriaceae
f__Morchellaceae
g__Morchella
s__Penicillium_bialowiezense
s__Aspergillus_aculeatus
s__Chaetomium_atrobrunneum
s__Acremonium_persicinum
s__Oidiodendron_rhodogenum
s__Aspergillus_tamarii
s__Mortierella_echinula
s__Aspergillus_flavus
s__Trichoderma_longibrachiatum
0
1
2
3
4
5
LDA SCORE (log 10)

Supplementary Figure 9. Oral carcinogenesis-associated alterations in the abundance of the plaque fungi examined using LEfSe. (A) Cladogram indicates the phylogenetic distribution of active fungi that were remarkably enriched. (B) Bar plots at the species level with significant differences in abundance based on LEfSe. The color bars show the LDA scores of species that were enriched in the indicated condition: purple bar (HC), green bar (OPL), and pink bar (OSCC).

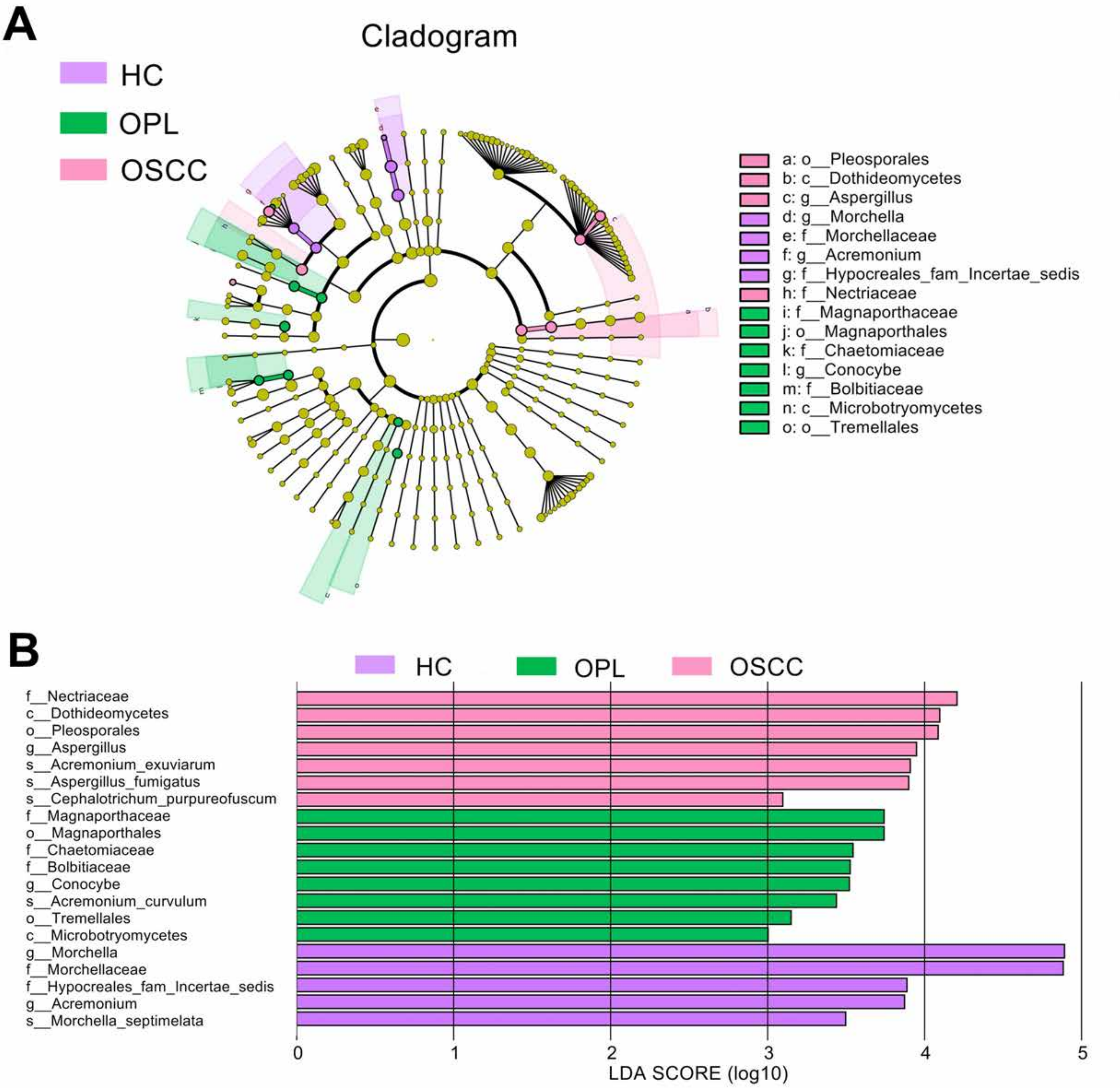
A
Cladogram
HC
OPL
OSCC
a: o__Pleosporales
b: c__Dothideomycetes
c: g__Aspergillus
d: g__Morchella
e: f__Morchellaceae
f: g__Acremonium
g: f__Hypocreales_fam_Incertae_sedis
h: f__Nectriaceae
i: f__Magnaporthaceae
j: o__Magnaporthales
k: f__Chaetomiaceae
l: g__Conocybe
m: f__Bolbitiaceae
n: c__Microbotryomycetes
o: o__Tremellales
B
HC
OPL
OSCC
f__Nectriaceae
c__Dothideomycetes
o__Pleosporales
g__Aspergillus
s__Acremonium_exuviarum
s__Aspergillus_fumigatus
s__Cephalotrichum_purpureofuscum
f__Magnaporthaceae
o__Magnaporthales
f__Chaetomiaceae
f__Bolbitiaceae
g__Conocybe
s__Acremonium_curvulum
o__Tremellales
c__Microbotryomycetes
g__Morchella
f__Morchellaceae
f__Hypocreales_fam_Incertae_sedis
g__Acremonium
s__Morchella_septimelata
0
1
2
3
4
5
LDA SCORE (log10)

Supplementary Figure 10. Oral carcinogenesis-associated alterations in the abundance of the saliva fungi examined using LEfSe. (A) Cladogram indicates the phylogenetic distribution of active fungi that were remarkably enriched. (B) Bar plots at the species level with significant differences in abundance based on LEfSe. The color bars show the LDA scores of species that were enriched in the indicated condition: purple bar (HC), green bar (OPL), and pink bar (OSCC).

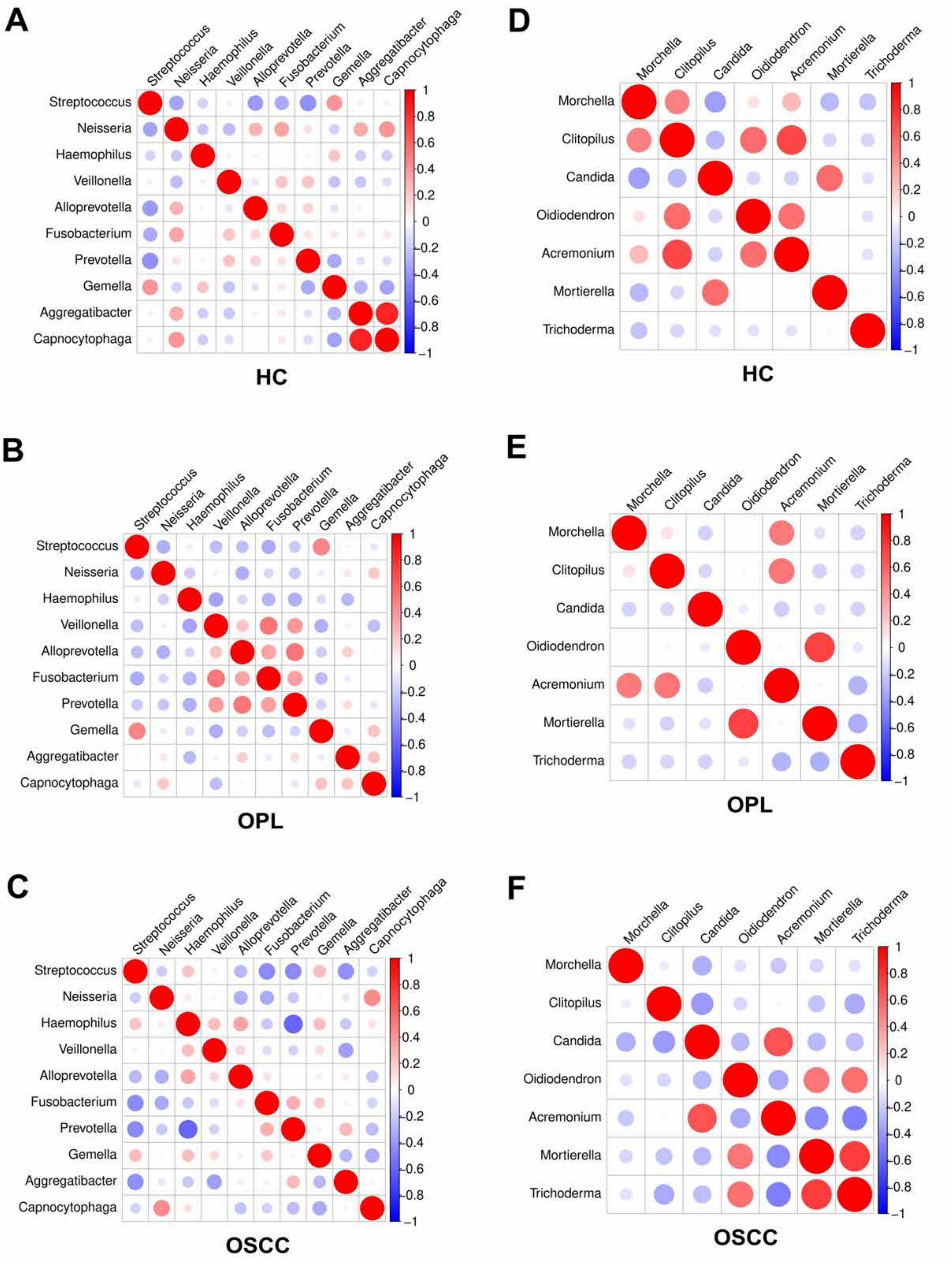
A
B
C
D
E
F
Streptococcus
Neisseria
Haemophilus
Veillonella
Alloprevotella
Fusobacterium
Prevotella
Gemella
Aggregatibacter
Capnocytophaga
Morchella
Clitopilus
Candida
Oidiodendron
Acremonium
Mortierella
Trichoderma
1
0.8
0.6
0.4
0.2
0
−0.2
−0.4
−0.6
−0.8
−1
HC
OPL
OSCC

Supplementary Figure 11. Intra-kingdom correlation at the genera level in buccal mucosal samples. Intra-kingdom correlations are shown between (A–C) bacterial and (D–F) fungal genera in the buccal mucosal samples from the HC, OPL, or OSCC groups. Red: positive correlation, blue: negative correlation. Circle size and color shading indicate the value of the correlation coefficient, with bigger circles with darker coloring representing higher coefficient values (maximum = 1) and smaller circles with lighter coloring representing lower coefficient values (minimum = 0).

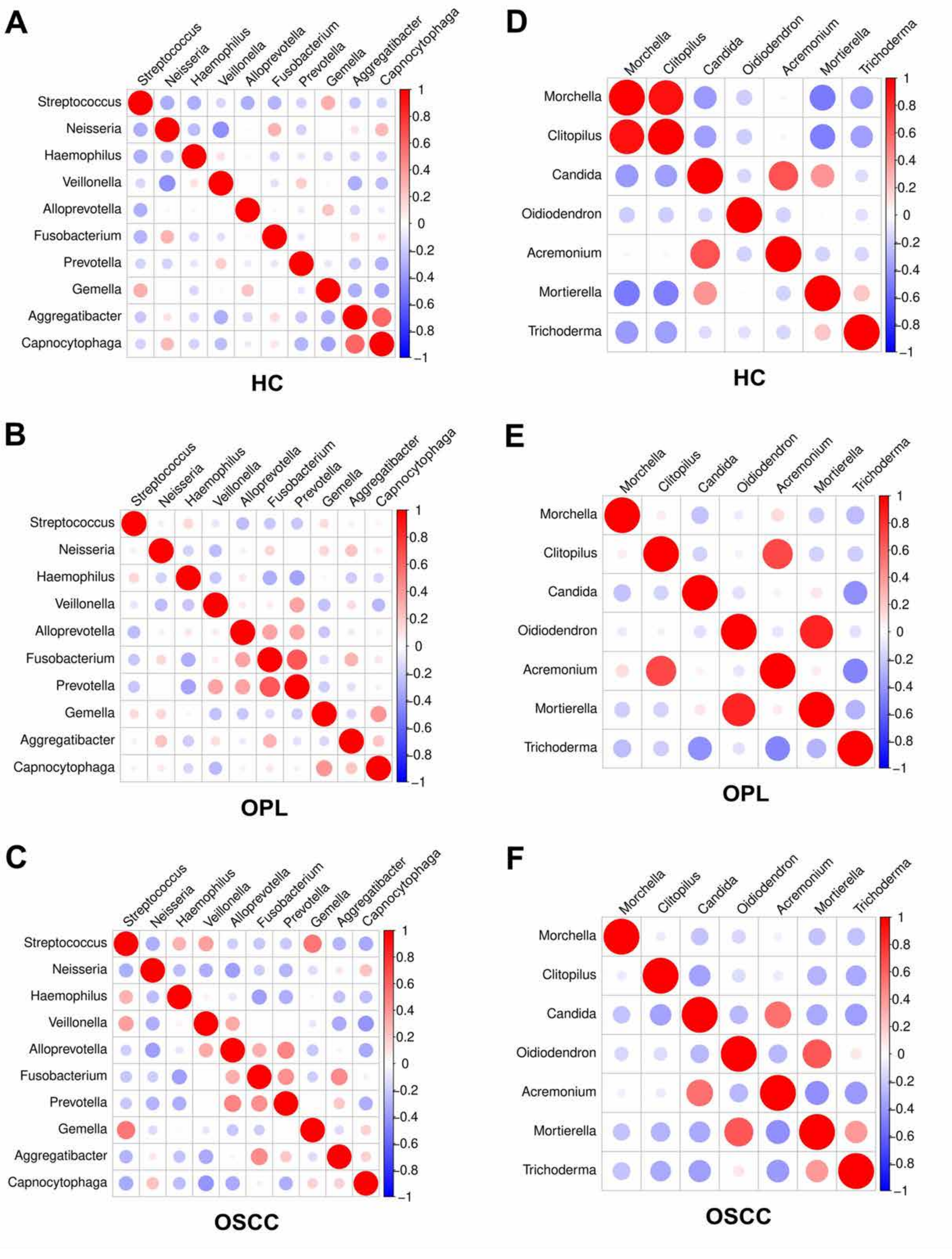
A
Streptococcus
Neisseria
Haemophilus
Veillonella
Alloprevotella
Fusobacterium
Prevotella
Gemella
Aggregatibacter
Capnocytophaga
1
0.8
0.6
0.4
0.2
0
−0.2
−0.4
−0.6
−0.8
−1
HC
D
Morchella
Clitopilus
Candida
Oidiodendron
Acremonium
Mortierella
Trichoderma
1
0.8
0.6
0.4
0.2
0
−0.2
−0.4
−0.6
−0.8
−1
HC
B
Streptococcus
Neisseria
Haemophilus
Veillonella
Alloprevotella
Fusobacterium
Prevotella
Gemella
Aggregatibacter
Capnocytophaga
1
0.8
0.6
0.4
0.2
0
−0.2
−0.4
−0.6
−0.8
−1
OPL
E
Morchella
Clitopilus
Candida
Oidiodendron
Acremonium
Mortierella
Trichoderma
1
0.8
0.6
0.4
0.2
0
−0.2
−0.4
−0.6
−0.8
−1
OPL
C
Streptococcus
Neisseria
Haemophilus
Veillonella
Alloprevotella
Fusobacterium
Prevotella
Gemella
Aggregatibacter
Capnocytophaga
1
0.8
0.6
0.4
0.2
0
−0.2
−0.4
−0.6
−0.8
−1
OSCC
F
Morchella
Clitopilus
Candida
Oidiodendron
Acremonium
Mortierella
Trichoderma
1
0.8
0.6
0.4
0.2
0
−0.2
−0.4
−0.6
−0.8
−1
OSCC

Supplementary Figure 12. Intra-kingdom correlation at the genera level in plaque samples. Intra-kingdom correlations are shown between (A–C) bacterial and (D–F) fungal genera in the plaque samples from the HC, OPL, or OSCC groups. Red: positive correlation, blue: negative correlation. Circle size and color shading indicate the value of the correlation coefficient, with bigger circles with darker coloring representing higher coefficient values (maximum = 1) and smaller circles with lighter coloring representing lower coefficient values (minimum = 0).

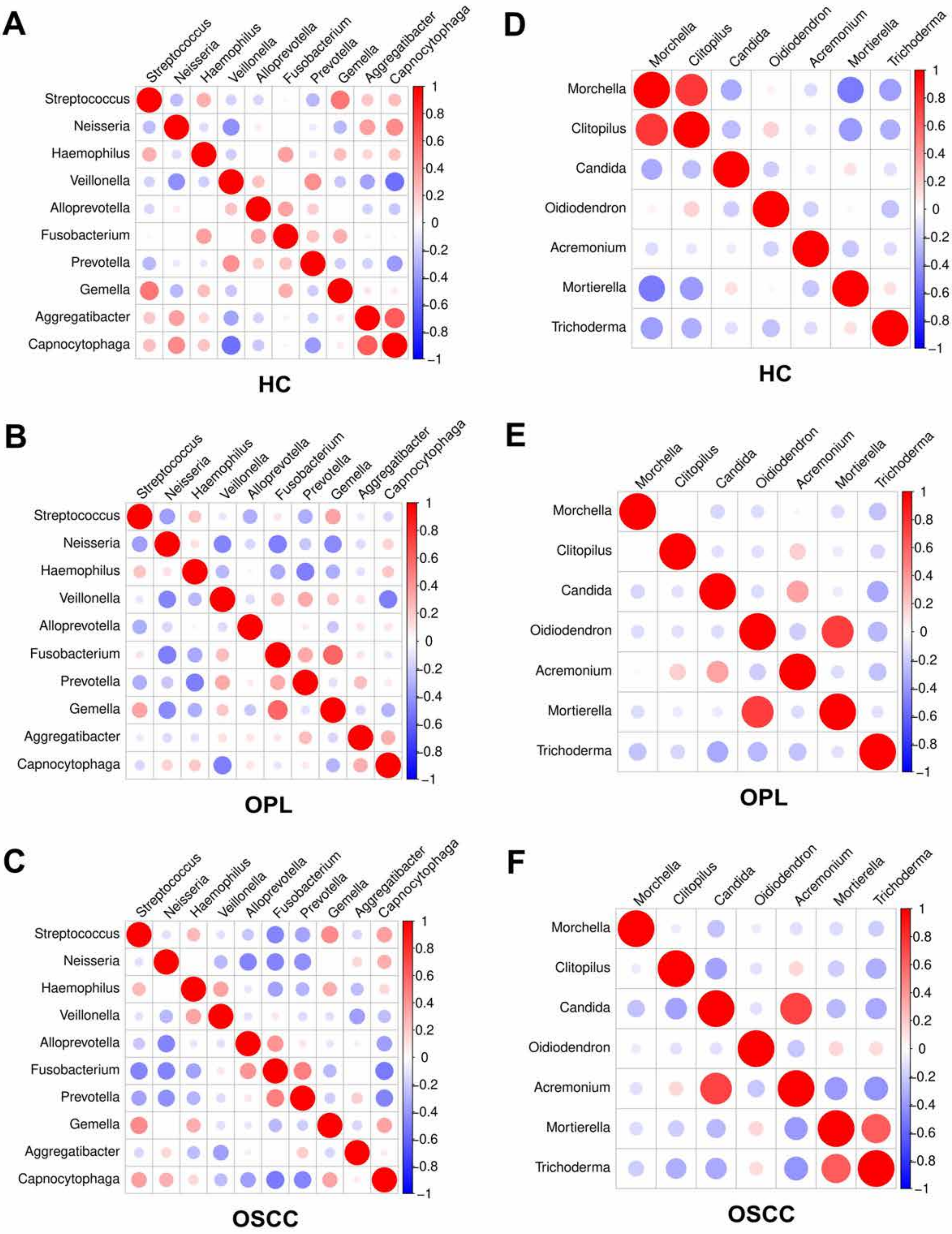
A
Streptococcus
Neisseria
Haemophilus
Veillonella
Alloprevotella
Fusobacterium
Prevotella
Gemella
Aggregatibacter
Capnocytophaga
1
0.8
0.6
0.4
0.2
0
−0.2
−0.4
−0.6
−0.8
−1
HC
D
Morchella
Clitopilus
Candida
Oidiodendron
Acremonium
Mortierella
Trichoderma
HC
B
OPL
E
OPL
C
OSCC
F
OSCC

Supplementary Figure 13. Intra-kingdom correlation at the genera level in saliva samples. Intra-kingdom correlations are shown between (A–C) bacterial and (D–F) fungal genera in the saliva samples from the HC, OPL, or OSCC groups. Red: positive correlation, blue: negative correlation. Circle size and color shading indicate the value of the correlation coefficient, with bigger circles with darker coloring representing higher coefficient values (maximum = 1) and smaller circles with lighter coloring representing lower coefficient values (minimum = 0).

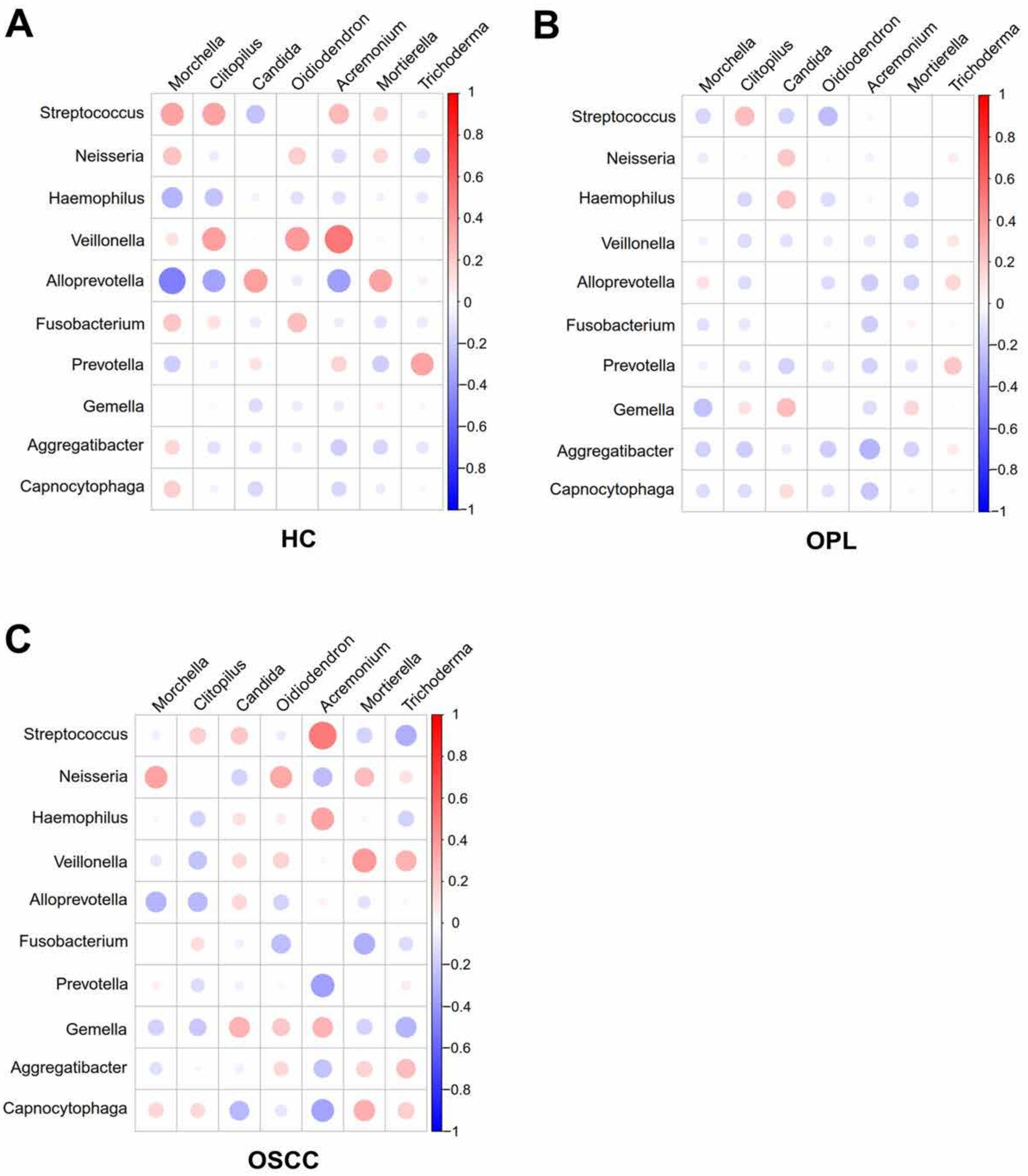
A
Morchella
Clitopilus
Candida
Oidiodendron
Acremonium
Mortierella
Trichoderma
Streptococcus
Neisseria
Haemophilus
Veillonella
Alloprevotella
Fusobacterium
Prevotella
Gemella
Aggregatibacter
Capnocytophaga
1
0.8
0.6
0.4
0.2
0
−0.2
−0.4
−0.6
−0.8
−1
HC
B
OPL
C
OSCC

Supplementary Figure 14. Inter-kingdom correlation at the genera level in buccal mucosal samples. Inter-kingdom correlations were observed between bacterial and fungal genera in the buccal mucosal samples from the (A) HC, (B) OPL, or (C) OSCC groups. Red: positive correlation, blue: negative correlation. Circle size and color shading indicate the value of the correlation coefficient, with bigger circles with darker coloring representing higher coefficient values (maximum = 1) and smaller circles with lighter coloring representing lower coefficient values (minimum = 0).

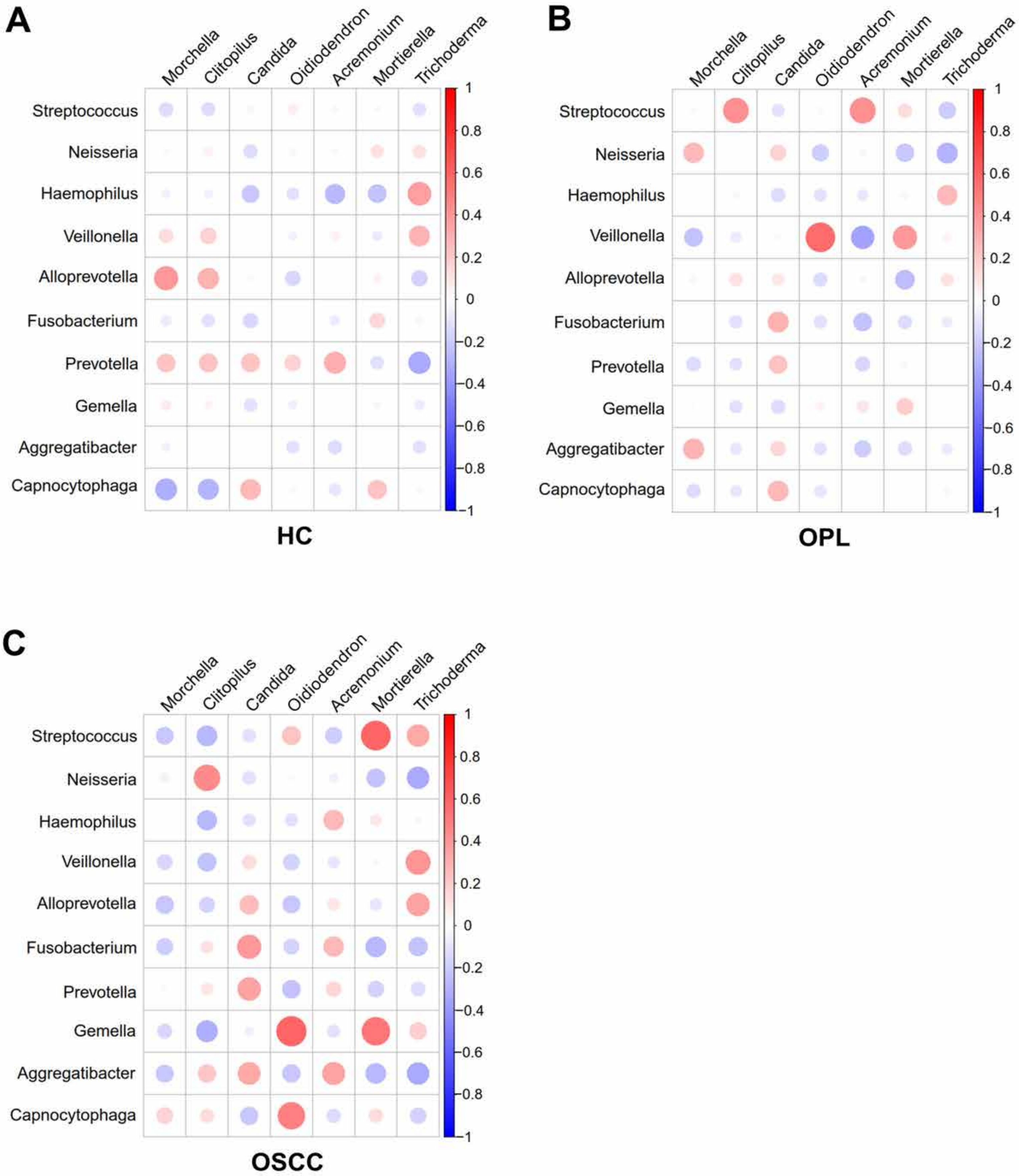
A
B
C
Morchella
Clitopilus
Candida
Oidiodendron
Acremonium
Mortierella
Trichoderma
Streptococcus
Neisseria
Haemophilus
Veillonella
Alloprevotella
Fusobacterium
Prevotella
Gemella
Aggregatibacter
Capnocytophaga
1
0.8
0.6
0.4
0.2
0
−0.2
−0.4
−0.6
−0.8
−1
HC
OPL
OSCC

Supplementary Figure 15. Inter-kingdom correlation at the genera level in plaque samples. Inter-kingdom correlations were observed between bacterial and fungal genera in the plaque samples from the (A) HC, (B) OPL, or (C) OSCC groups. Red: positive correlation, blue: negative correlation. Circle size and color shading indicate the value of the correlation coefficient, with bigger circles with darker coloring representing higher coefficient values (maximum = 1) and smaller circles with lighter coloring representing lower coefficient values (minimum = 0).

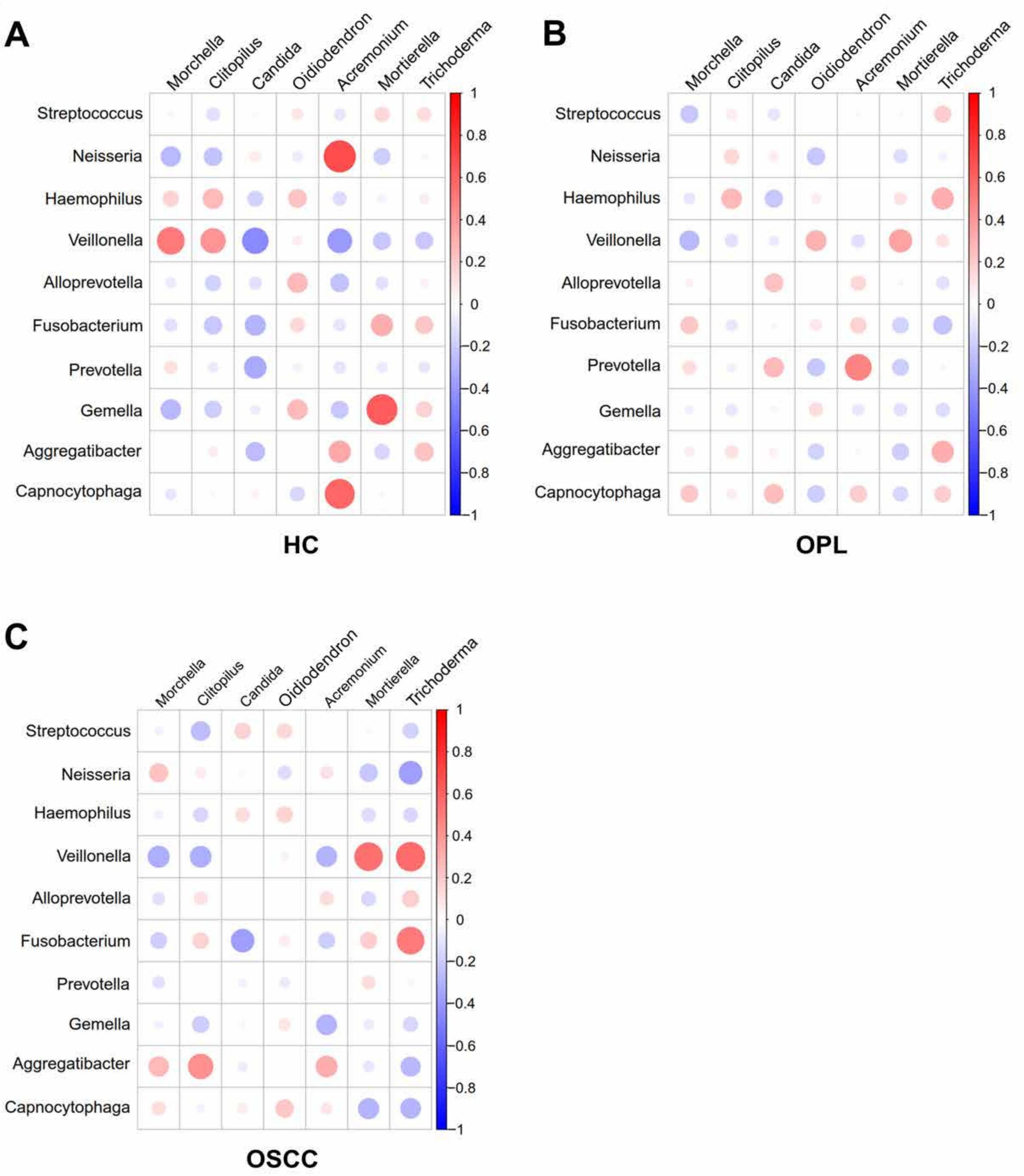

A
Morchella
Clitopilus
Candida
Oidiodendron
Acremonium
Mortierella
Trichoderma
Streptococcus
Neisseria
Haemophilus
Veillonella
Alloprevotella
Fusobacterium
Prevotella
Gemella
Aggregatibacter
Capnocytophaga
1
0.8
0.6
0.4
0.2
0
−0.2
−0.4
−0.6
−0.8
−1
HC
B
Morchella
Clitopilus
Candida
Oidiodendron
Acremonium
Mortierella
Trichoderma
Streptococcus
Neisseria
Haemophilus
Veillonella
Alloprevotella
Fusobacterium
Prevotella
Gemella
Aggregatibacter
Capnocytophaga
1
0.8
0.6
0.4
0.2
0
−0.2
−0.4
−0.6
−0.8
−1
OPL
C
Morchella
Clitopilus
Candida
Oidiodendron
Acremonium
Mortierella
Trichoderma
Streptococcus
Neisseria
Haemophilus
Veillonella
Alloprevotella
Fusobacterium
Prevotella
Gemella
Aggregatibacter
Capnocytophaga
1
0.8
0.6
0.4
0.2
0
−0.2
−0.4
−0.6
−0.8
−1
OSCC

Supplementary Figure 16. Inter-kingdom correlation at the genera level in saliva samples. Inter-kingdom correlations were observed between bacterial and fungal genera in the saliva samples from the (A) HC, (B) OPL, or (C) OSCC groups. Red: positive correlation, blue: negative correlation. Circle size and color shading indicate the value of the correlation coefficient, with bigger circles with darker coloring representing higher coefficient values (maximum = 1) and smaller circles with lighter coloring representing lower coefficient values (minimum = 0).

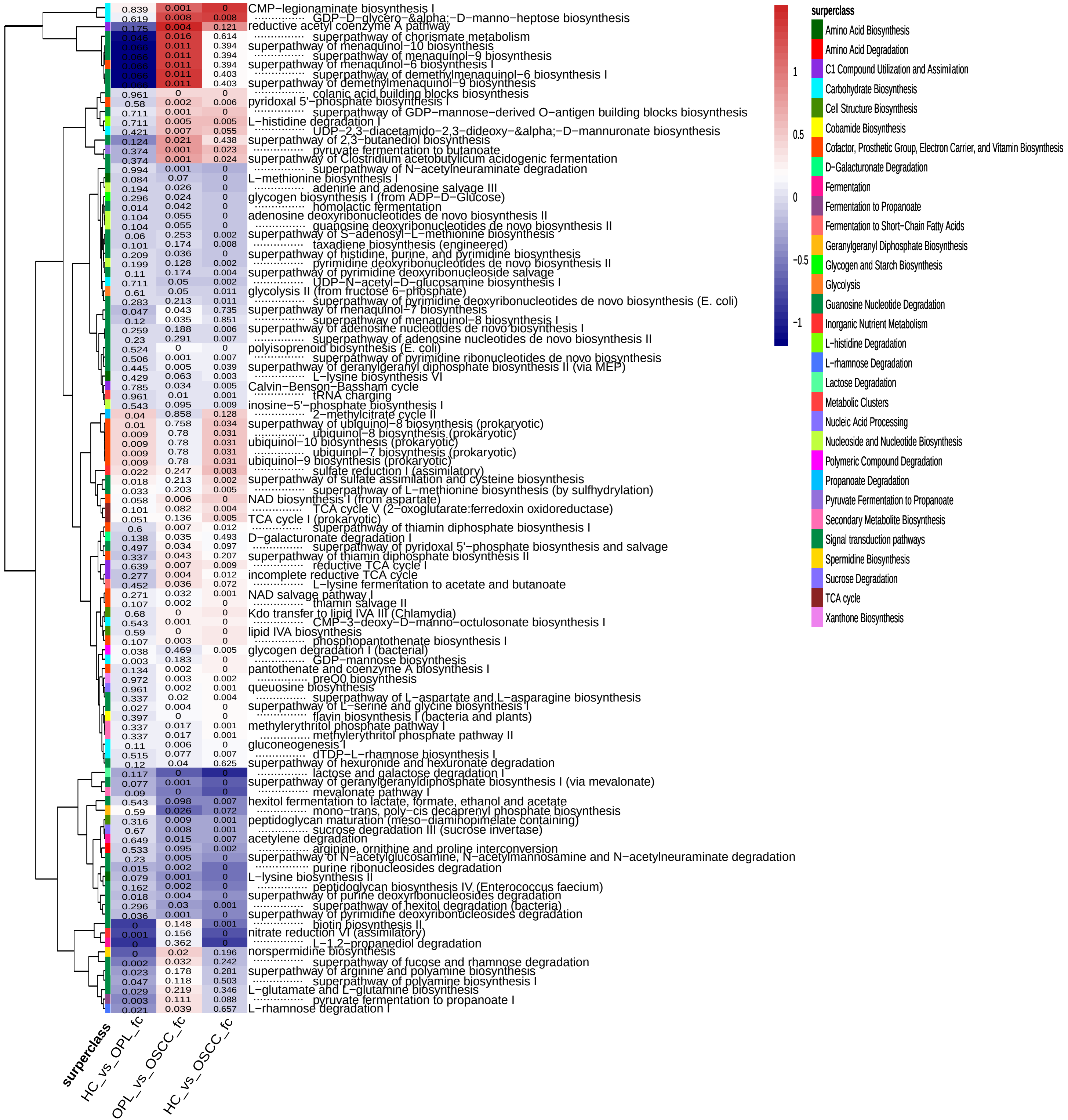

CMP−legionaminate biosynthesis I
GDP−D−glycero−α−D−manno−heptose biosynthesis
reductive acetyl coenzyme A pathway
superpathway of chorismate metabolism
superpathway of menaquinol−10 biosynthesis
superpathway of menaquinol−9 biosynthesis
superpathway of menaquinol−6 biosynthesis I
superpathway of demethylmenaquinol−6 biosynthesis I
superpathway of demethylmenaquinol−9 biosynthesis
colanic acid building blocks biosynthesis
pyridoxal 5'−phosphate biosynthesis I
superpathway of GDP−mannose−derived O−antigen building blocks biosynthesis
L−histidine degradation I
UDP−2,3−diacetamido−2,3−dideoxy−α−D−mannuronate biosynthesis
superpathway of 2,3−butanediol biosynthesis
pyruvate fermentation to butanoate
superpathway of Clostridium acetobutylicum acidogenic fermentation
superpathway of N−acetylneuraminate degradation
L−methionine biosynthesis I
adenine and adenosine salvage III
glycogen biosynthesis I (from ADP−D−Glucose)
homolactic fermentation
adenosine deoxyribonucleotides de novo biosynthesis II
guanosine deoxyribonucleotides de novo biosynthesis II
superpathway of S−adenosyl−L−methionine biosynthesis
taxadiene biosynthesis (engineered)
superpathway of histidine, purine, and pyrimidine biosynthesis
pyrimidine deoxyribonucleotides de novo biosynthesis II
superpathway of pyrimidine deoxyribonucleoside salvage
UDP−N−acetyl−D−glucosamine biosynthesis I
glycolysis II (from fructose 6−phosphate)
superpathway of pyrimidine deoxyribonucleotides de novo biosynthesis (E. coli)
superpathway of menaquinol−7 biosynthesis
superpathway of menaquinol−8 biosynthesis I
superpathway of adenosine nucleotides de novo biosynthesis I
superpathway of adenosine nucleotides de novo biosynthesis II
polyisoprenoid biosynthesis (E. coli)
superpathway of pyrimidine ribonucleotides de novo biosynthesis
superpathway of geranylgeranyl diphosphate biosynthesis II (via MEP)
L−lysine biosynthesis VI
Calvin−Benson−Bassham cycle
tRNA charging
inosine−5'−phosphate biosynthesis I
2−methylcitrate cycle II
superpathway of ubiquinol−8 biosynthesis (prokaryotic)
ubiquinol−8 biosynthesis (prokaryotic)
ubiquinol−10 biosynthesis (prokaryotic)
ubiquinol−7 biosynthesis (prokaryotic)
ubiquinol−9 biosynthesis (prokaryotic)
sulfate reduction I (assimilatory)
superpathway of sulfate assimilation and cysteine biosynthesis
superpathway of L−methionine biosynthesis (by sulfhydrylation)
NAD biosynthesis I (from aspartate)
TCA cycle V (2−oxoglutarate:ferredoxin oxidoreductase)
TCA cycle I (prokaryotic)
superpathway of thiamin diphosphate biosynthesis I
D−galacturonate degradation I
superpathway of pyridoxal 5'−phosphate biosynthesis and salvage
superpathway of thiamin diphosphate biosynthesis II
reductive TCA cycle I
incomplete reductive TCA cycle
L−lysine fermentation to acetate and butanoate
NAD salvage pathway I
thiamin salvage II
Kdo transfer to lipid IVA III (Chlamydia)
CMP−3−deoxy−D−manno−octulosonate biosynthesis I
lipid IVA biosynthesis
phosphopantothenate biosynthesis I
glycogen degradation I (bacterial)
GDP−mannose biosynthesis
pantothenate and coenzyme A biosynthesis I
preQ0 biosynthesis
queuosine biosynthesis
superpathway of L−aspartate and L−asparagine biosynthesis
superpathway of L−serine and glycine biosynthesis I
flavin biosynthesis I (bacteria and plants)
methylerythritol phosphate pathway I
methylerythritol phosphate pathway II
gluconeogenesis I
dTDP−L−rhamnose biosynthesis I
superpathway of hexuronide and hexuronate degradation
lactose and galactose degradation I
superpathway of geranylgeranyldiphosphate biosynthesis I (via mevalonate)
mevalonate pathway I
hexitol fermentation to lactate, formate, ethanol and acetate
mono−trans, poly−cis decaprenyl phosphate biosynthesis
peptidoglycan maturation (meso−diaminopimelate containing)
sucrose degradation III (sucrose invertase)
acetylene degradation
arginine, ornithine and proline interconversion
superpathway of N−acetylglucosamine, N−acetylmannosamine and N−acetylneuraminate degradation
purine ribonucleosides degradation
L−lysine biosynthesis II
peptidoglycan biosynthesis IV (Enterococcus faecium)
superpathway of purine deoxyribonucleosides degradation
superpathway of hexitol degradation (bacteria)
superpathway of pyrimidine deoxyribonucleosides degradation
biotin biosynthesis II
nitrate reduction VI (assimilatory)
L−1,2−propanediol degradation
norspermidine biosynthesis
superpathway of fucose and rhamnose degradation
superpathway of arginine and polyamine biosynthesis
superpathway of polyamine biosynthesis I
L−glutamate and L−glutamine biosynthesis
pyruvate fermentation to propanoate I
L−rhamnose degradation I
superclass
HC_vs_OPL_fc
OPL_vs_OSCC_fc
HC_vs_OSCC_fc
1
0.5
0
−0.5
−1
superclass
Amino Acid Biosynthesis
Amino Acid Degradation
C1 Compound Utilization and Assimilation
Carbohydrate Biosynthesis
Cell Structure Biosynthesis
Cobamide Biosynthesis
Cofactor, Prosthetic Group, Electron Carrier, and Vitamin Biosynthesis
D−Galacturonate Degradation
Fermentation
Fermentation to Propanoate
Fermentation to Short−Chain Fatty Acids
Geranylgeranyl Diphosphate Biosynthesis
Glycogen and Starch Biosynthesis
Glycolysis
Guanosine Nucleotide Degradation
Inorganic Nutrient Metabolism
L−histidine Degradation
L−rhamnose Degradation
Lactose Degradation
Metabolic Clusters
Nucleic Acid Processing
Nucleoside and Nucleotide Biosynthesis
Polymeric Compound Degradation
Propanoate Degradation
Pyruvate Fermentation to Propanoate
Secondary Metabolite Biosynthesis
Signal transduction pathways
Spermidine Biosynthesis
Sucrose Degradation
TCA cycle
Xanthone Biosynthesis

Supplementary Figure 17. Functional alterations in the plaque bacteriome. The relative abundance of functional pathways was compared among HC, OPL, and OSCC individuals. Differentially abundant pathways were plotted, and the exact *P* values are presented in the heatmap. Generalized fold change is indicated by color gradients. The generalized fold change > 0: enriched in the latter; generalized fold change < 0: enriched in the former.

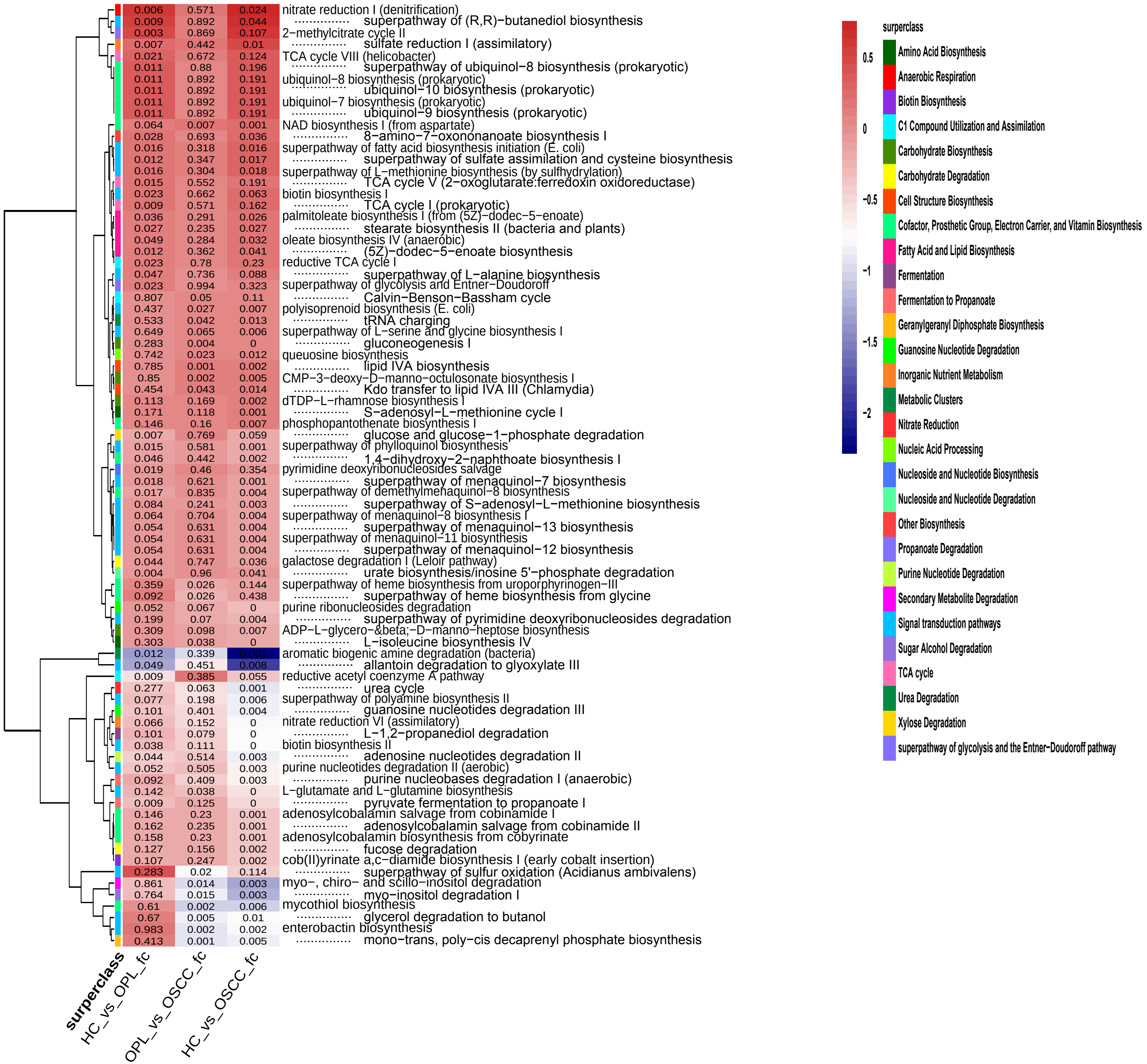
0.006 0.571 0.024 nitrate reduction I (denitrification)
0.009 0.892 0.044 superpathway of (R,R)−butanediol biosynthesis
0.003 0.869 0.107 2−methylcitrate cycle II
0.007 0.442 0.01 sulfate reduction I (assimilatory)
0.021 0.672 0.124 TCA cycle VIII (helicobacter)
0.011 0.88 0.196 superpathway of ubiquinol−8 biosynthesis (prokaryotic)
0.011 0.892 0.191 ubiquinol−8 biosynthesis (prokaryotic)
0.011 0.892 0.191 ubiquinol−10 biosynthesis (prokaryotic)
0.011 0.892 0.191 ubiquinol−7 biosynthesis (prokaryotic)
0.011 0.892 0.191 ubiquinol−9 biosynthesis (prokaryotic)
0.064 0.007 0.001 NAD biosynthesis I (from aspartate)
0.028 0.693 0.036 8−amino−7−oxononanoate biosynthesis I
0.016 0.318 0.016 superpathway of fatty acid biosynthesis initiation (E. coli)
0.012 0.347 0.017 superpathway of sulfate assimilation and cysteine biosynthesis
0.016 0.304 0.018 superpathway of L−methionine biosynthesis (by sulfhydrylation)
0.015 0.552 0.191 TCA cycle V (2−oxoglutarate:ferredoxin oxidoreductase)
0.023 0.662 0.063 biotin biosynthesis I
0.009 0.571 0.162 TCA cycle I (prokaryotic)
0.036 0.291 0.026 palmitoleate biosynthesis I (from (5Z)−dodec−5−enoate)
0.027 0.235 0.027 stearate biosynthesis II (bacteria and plants)
0.049 0.284 0.032 oleate biosynthesis IV (anaerobic)
0.012 0.362 0.041 (5Z)−dodec−5−enoate biosynthesis
0.023 0.78 0.23 reductive TCA cycle I
0.047 0.736 0.088 superpathway of L−alanine biosynthesis
0.023 0.994 0.323 superpathway of glycolysis and Entner−Doudoroff
0.807 0.05 0.11 Calvin−Benson−Bassham cycle
0.437 0.027 0.007 polyisoprenoid biosynthesis (E. coli)
0.533 0.042 0.013 tRNA charging
0.649 0.065 0.006 superpathway of L−serine and glycine biosynthesis I
0.283 0.004 0 gluconeogenesis I
0.742 0.023 0.012 queuosine biosynthesis
0.785 0.001 0.002 lipid IVA biosynthesis
0.85 0.002 0.005 CMP−3−deoxy−D−manno−octulosonate biosynthesis I
0.454 0.043 0.014 Kdo transfer to lipid IVA III (Chlamydia)
0.113 0.169 0.002 dTDP−L−rhamnose biosynthesis I
0.171 0.118 0.001 S−adenosyl−L−methionine cycle I
0.146 0.16 0.007 phosphopantothenate biosynthesis I
0.007 0.769 0.059 glucose and glucose−1−phosphate degradation
0.015 0.581 0.001 superpathway of phylloquinol biosynthesis
0.046 0.442 0.002 1,4−dihydroxy−2−naphthoate biosynthesis I
0.019 0.46 0.354 pyrimidine deoxyribonucleosides salvage
0.018 0.621 0.001 superpathway of menaquinol−7 biosynthesis
0.017 0.835 0.004 superpathway of demethylmenaquinol−8 biosynthesis
0.084 0.241 0.003 superpathway of S−adenosyl−L−methionine biosynthesis
0.064 0.704 0.004 superpathway of menaquinol−8 biosynthesis I
0.054 0.631 0.004 superpathway of menaquinol−13 biosynthesis
0.054 0.631 0.004 superpathway of menaquinol−11 biosynthesis
0.054 0.631 0.004 superpathway of menaquinol−12 biosynthesis
0.044 0.747 0.036 galactose degradation I (Leloir pathway)
0.004 0.96 0.041 urate biosynthesis/inosine 5'−phosphate degradation
0.359 0.026 0.144 superpathway of heme biosynthesis from uroporphyrinogen−III
0.092 0.026 0.438 superpathway of heme biosynthesis from glycine
0.052 0.067 0 purine ribonucleosides degradation
0.199 0.07 0.004 superpathway of pyrimidine deoxyribonucleosides degradation
0.309 0.098 0.007 ADP−L−glycero−β−D−manno−heptose biosynthesis
0.303 0.038 0 L−isoleucine biosynthesis IV
0.012 0.339 0.001 aromatic biogenic amine degradation (bacteria)
0.049 0.451 0.008 allantoin degradation to glyoxylate III
0.009 0.385 0.055 reductive acetyl coenzyme A pathway
0.277 0.063 0.001 urea cycle
0.077 0.198 0.006 superpathway of polyamine biosynthesis II
0.101 0.401 0.004 guanosine nucleotides degradation III
0.066 0.152 0 nitrate reduction VI (assimilatory)
0.101 0.079 0 L−1,2−propanediol degradation
0.038 0.111 0 biotin biosynthesis II
0.044 0.514 0.003 adenosine nucleotides degradation II
0.052 0.505 0.003 purine nucleotides degradation II (aerobic)
0.092 0.409 0.003 purine nucleobases degradation I (anaerobic)
0.142 0.038 0 L−glutamate and L−glutamine biosynthesis
0.009 0.125 0 pyruvate fermentation to propanoate I
0.146 0.23 0.001 adenosylcobalamin salvage from cobinamide I
0.162 0.235 0.001 adenosylcobalamin salvage from cobinamide II
0.158 0.23 0.001 adenosylcobalamin biosynthesis from cobyrinate
0.127 0.156 0.002 fucose degradation
0.107 0.247 0.002 cob(II)yrinate a,c−diamide biosynthesis I (early cobalt insertion)
0.283 0.02 0.114 superpathway of sulfur oxidation (Acidianus ambivalens)
0.861 0.014 0.003 myo−, chiro− and scillo−inositol degradation
0.764 0.015 0.003 myo−inositol degradation I
0.61 0.002 0.006 mycothiol biosynthesis
0.67 0.005 0.01 glycerol degradation to butanol
0.983 0.002 0.002 enterobactin biosynthesis
0.413 0.001 0.005 mono−trans, poly−cis decaprenyl phosphate biosynthesis
superclass
HC_vs_OPL_fc
OPL_vs_OSCC_fc
HC_vs_OSCC_fc
0.5
0
−0.5
−1
−1.5
−2
superclass
Amino Acid Biosynthesis
Anaerobic Respiration
Biotin Biosynthesis
C1 Compound Utilization and Assimilation
Carbohydrate Biosynthesis
Carbohydrate Degradation
Cell Structure Biosynthesis
Cofactor, Prosthetic Group, Electron Carrier, and Vitamin Biosynthesis
Fatty Acid and Lipid Biosynthesis
Fermentation
Fermentation to Propanoate
Geranylgeranyl Diphosphate Biosynthesis
Guanosine Nucleotide Degradation
Inorganic Nutrient Metabolism
Metabolic Clusters
Nitrate Reduction
Nucleic Acid Processing
Nucleoside and Nucleotide Biosynthesis
Nucleoside and Nucleotide Degradation
Other Biosynthesis
Propanoate Degradation
Purine Nucleotide Degradation
Secondary Metabolite Degradation
Signal transduction pathways
Sugar Alcohol Degradation
TCA cycle
Urea Degradation
Xylose Degradation
superpathway of glycolysis and the Entner−Doudoroff pathway

Supplementary Figure 18. Functional alterations in the saliva bacteriome. The relative abundance of functional pathways was compared among HC, OPL, and OSCC individuals. Differentially abundant pathways were plotted, and the exact *P* values are presented in the heatmap. Generalized fold change is indicated by color gradients. The generalized fold change > 0: enriched in the latter; generalized fold change < 0: enriched in the former.

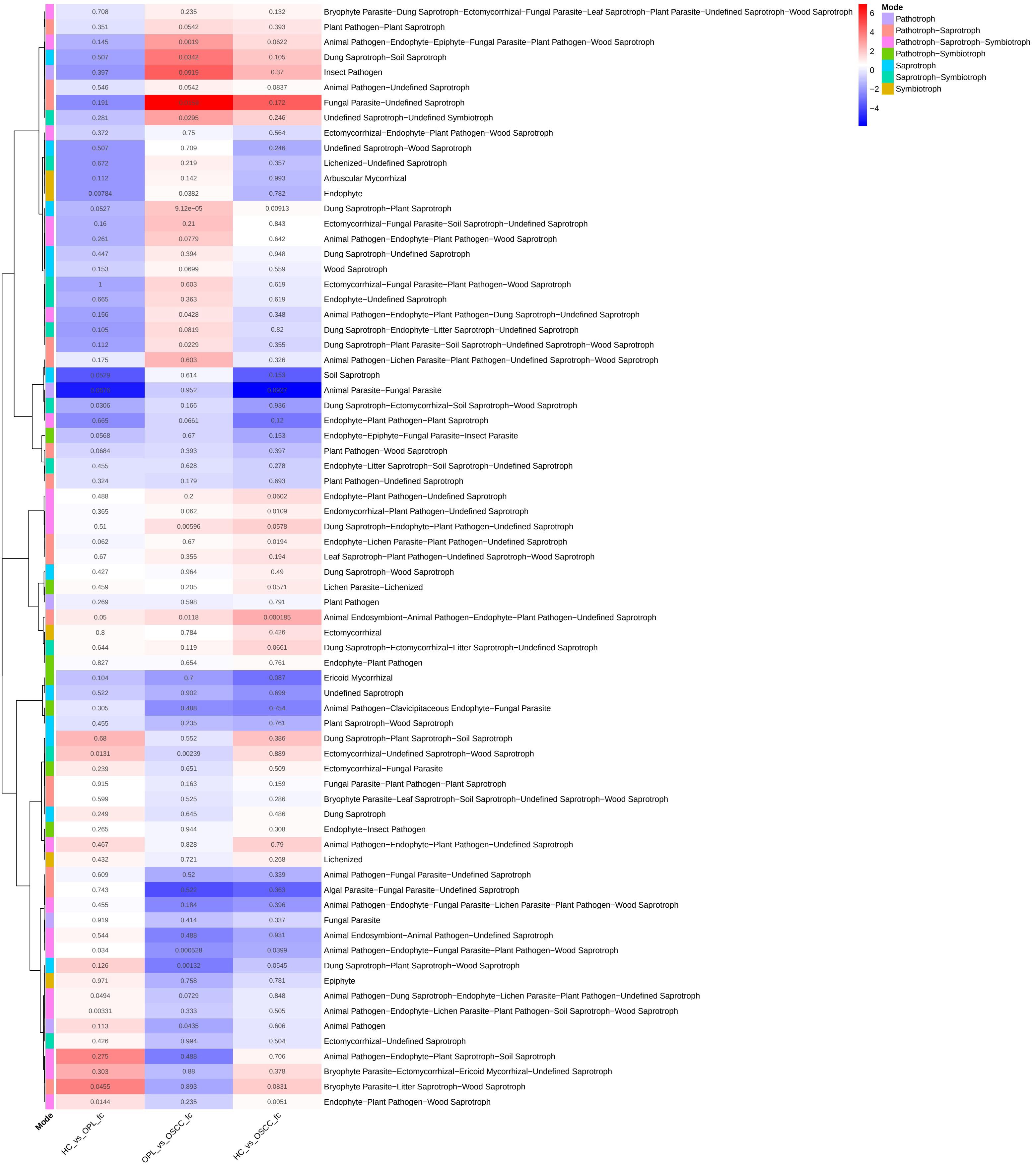
Mode
Pathotroph
Pathotroph−Saprotroph
Pathotroph−Saprotroph−Symbiotroph
Pathotroph−Symbiotroph
Saprotroph
Saprotroph−Symbiotroph
Symbiotroph
6
4
2
0
−2
−4
0.708 0.235 0.132 Bryophyte Parasite−Dung Saprotroph−Ectomycorrhizal−Fungal Parasite−Leaf Saprotroph−Plant Parasite−Undefined Saprotroph−Wood Saprotroph
0.351 0.0542 0.393 Plant Pathogen−Plant Saprotroph
0.145 0.0019 0.0622 Animal Pathogen−Endophyte−Epiphyte−Fungal Parasite−Plant Pathogen−Wood Saprotroph
0.507 0.0342 0.105 Dung Saprotroph−Soil Saprotroph
0.397 0.0919 0.37 Insect Pathogen
0.546 0.0542 0.0837 Animal Pathogen−Undefined Saprotroph
0.191 0.172 Fungal Parasite−Undefined Saprotroph
0.281 0.0295 0.246 Undefined Saprotroph−Undefined Symbiotroph
0.372 0.75 0.564 Ectomycorrhizal−Endophyte−Plant Pathogen−Wood Saprotroph
0.507 0.709 0.246 Undefined Saprotroph−Wood Saprotroph
0.672 0.219 0.357 Lichenized−Undefined Saprotroph
0.112 0.142 0.993 Arbuscular Mycorrhizal
0.00784 0.0382 0.782 Endophyte
0.0527 9.12e−05 0.00913 Dung Saprotroph−Plant Saprotroph
0.16 0.21 0.843 Ectomycorrhizal−Fungal Parasite−Soil Saprotroph−Undefined Saprotroph
0.261 0.0779 0.642 Animal Pathogen−Endophyte−Plant Pathogen−Wood Saprotroph
0.447 0.394 0.948 Dung Saprotroph−Undefined Saprotroph
0.153 0.0699 0.559 Wood Saprotroph
1 0.603 0.619 Ectomycorrhizal−Fungal Parasite−Plant Pathogen−Wood Saprotroph
0.665 0.363 0.619 Endophyte−Undefined Saprotroph
0.156 0.0428 0.348 Animal Pathogen−Endophyte−Plant Pathogen−Dung Saprotroph−Undefined Saprotroph
0.105 0.0819 0.82 Dung Saprotroph−Endophyte−Litter Saprotroph−Undefined Saprotroph
0.112 0.0229 0.355 Dung Saprotroph−Plant Parasite−Soil Saprotroph−Undefined Saprotroph−Wood Saprotroph
0.175 0.603 0.326 Animal Pathogen−Lichen Parasite−Plant Pathogen−Undefined Saprotroph−Wood Saprotroph
0.0529 0.614 0.153 Soil Saprotroph
0.952 Animal Parasite−Fungal Parasite
0.0306 0.166 0.936 Dung Saprotroph−Ectomycorrhizal−Soil Saprotroph−Wood Saprotroph
0.665 0.0661 0.12 Endophyte−Plant Pathogen−Plant Saprotroph
0.0568 0.67 0.153 Endophyte−Epiphyte−Fungal Parasite−Insect Parasite
0.0684 0.393 0.397 Plant Pathogen−Wood Saprotroph
0.455 0.628 0.278 Endophyte−Litter Saprotroph−Soil Saprotroph−Undefined Saprotroph
0.324 0.179 0.693 Plant Pathogen−Undefined Saprotroph
0.488 0.2 0.0602 Endophyte−Plant Pathogen−Undefined Saprotroph
0.365 0.062 0.0109 Endomycorrhizal−Plant Pathogen−Undefined Saprotroph
0.51 0.00596 0.0578 Dung Saprotroph−Endophyte−Plant Pathogen−Undefined Saprotroph
0.062 0.67 0.0194 Endophyte−Lichen Parasite−Plant Pathogen−Undefined Saprotroph
0.67 0.355 0.194 Leaf Saprotroph−Plant Pathogen−Undefined Saprotroph−Wood Saprotroph
0.427 0.964 0.49 Dung Saprotroph−Wood Saprotroph
0.459 0.205 0.0571 Lichen Parasite−Lichenized
0.269 0.598 0.791 Plant Pathogen
0.05 0.0118 0.000185 Animal Endosymbiont−Animal Pathogen−Endophyte−Plant Pathogen−Undefined Saprotroph
0.8 0.784 0.426 Ectomycorrhizal
0.644 0.119 0.0661 Dung Saprotroph−Ectomycorrhizal−Litter Saprotroph−Undefined Saprotroph
0.827 0.654 0.761 Endophyte−Plant Pathogen
0.104 0.7 0.087 Ericoid Mycorrhizal
0.522 0.902 0.699 Undefined Saprotroph
0.305 0.488 0.754 Animal Pathogen−Clavicipitaceous Endophyte−Fungal Parasite
0.455 0.235 0.761 Plant Saprotroph−Wood Saprotroph
0.68 0.552 0.386 Dung Saprotroph−Plant Saprotroph−Soil Saprotroph
0.0131 0.00239 0.889 Ectomycorrhizal−Undefined Saprotroph−Wood Saprotroph
0.239 0.651 0.509 Ectomycorrhizal−Fungal Parasite
0.915 0.163 0.159 Fungal Parasite−Plant Pathogen−Plant Saprotroph
0.599 0.525 0.286 Bryophyte Parasite−Leaf Saprotroph−Soil Saprotroph−Undefined Saprotroph−Wood Saprotroph
0.249 0.645 0.486 Dung Saprotroph
0.265 0.944 0.308 Endophyte−Insect Pathogen
0.467 0.828 0.79 Animal Pathogen−Endophyte−Plant Pathogen−Undefined Saprotroph
0.432 0.721 0.268 Lichenized
0.609 0.52 0.339 Animal Pathogen−Fungal Parasite−Undefined Saprotroph
0.743 Algal Parasite−Fungal Parasite−Undefined Saprotroph
0.455 0.184 0.396 Animal Pathogen−Endophyte−Fungal Parasite−Lichen Parasite−Plant Pathogen−Wood Saprotroph
0.919 0.414 0.337 Fungal Parasite
0.544 0.488 0.931 Animal Endosymbiont−Animal Pathogen−Undefined Saprotroph
0.034 0.000528 0.0399 Animal Pathogen−Endophyte−Fungal Parasite−Plant Pathogen−Wood Saprotroph
0.126 0.00132 0.0545 Dung Saprotroph−Plant Saprotroph−Wood Saprotroph
0.971 0.758 0.781 Epiphyte
0.0494 0.0729 0.848 Animal Pathogen−Dung Saprotroph−Endophyte−Lichen Parasite−Plant Pathogen−Undefined Saprotroph
0.00331 0.333 0.505 Animal Pathogen−Endophyte−Lichen Parasite−Plant Pathogen−Soil Saprotroph−Wood Saprotroph
0.113 0.0435 0.606 Animal Pathogen
0.426 0.994 0.504 Ectomycorrhizal−Undefined Saprotroph
0.275 0.488 0.706 Animal Pathogen−Endophyte−Plant Saprotroph−Soil Saprotroph
0.303 0.88 0.378 Bryophyte Parasite−Ectomycorrhizal−Ericoid Mycorrhizal−Undefined Saprotroph
0.0455 0.893 0.0831 Bryophyte Parasite−Litter Saprotroph−Wood Saprotroph
0.0144 0.235 0.0051 Endophyte−Plant Pathogen−Wood Saprotroph
Mode
HC_vs_OPL_fc
OPL_vs_OSCC_fc
HC_vs_OSCC_fc

Supplementary Figure 19. Functional alterations in the plaque mycobiome. The relative abundance of functional pathways was compared among HC, OPL, and OSCC individuals. Differentially abundant pathways were plotted, and the exact *P* values are presented in the heatmap. Generalized fold change is indicated by color gradients. The generalized fold change > 0: enriched in the latter; generalized fold change < 0: enriched in the former.

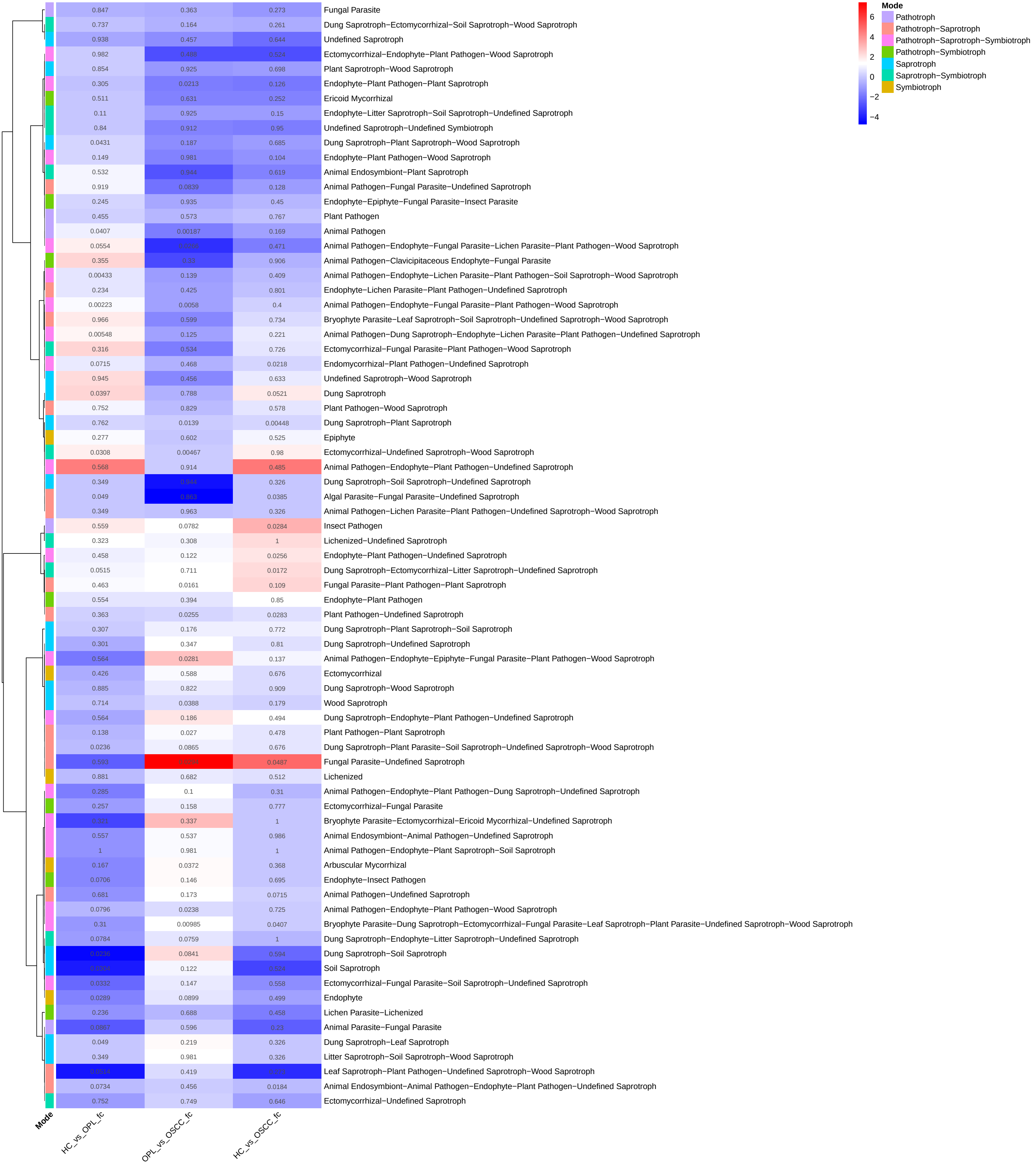
Mode
Pathotroph
Pathotroph−Saprotroph
Pathotroph−Saprotroph−Symbiotroph
Pathotroph−Symbiotroph
Saprotroph
Saprotroph−Symbiotroph
Symbiotroph
6
4
2
0
−2
−4
Fungal Parasite
Dung Saprotroph−Ectomycorrhizal−Soil Saprotroph−Wood Saprotroph
Undefined Saprotroph
Ectomycorrhizal−Endophyte−Plant Pathogen−Wood Saprotroph
Plant Saprotroph−Wood Saprotroph
Endophyte−Plant Pathogen−Plant Saprotroph
Ericoid Mycorrhizal
Endophyte−Litter Saprotroph−Soil Saprotroph−Undefined Saprotroph
Undefined Saprotroph−Undefined Symbiotroph
Dung Saprotroph−Plant Saprotroph−Wood Saprotroph
Endophyte−Plant Pathogen−Wood Saprotroph
Animal Endosymbiont−Plant Saprotroph
Animal Pathogen−Fungal Parasite−Undefined Saprotroph
Endophyte−Epiphyte−Fungal Parasite−Insect Parasite
Plant Pathogen
Animal Pathogen
Animal Pathogen−Endophyte−Fungal Parasite−Lichen Parasite−Plant Pathogen−Wood Saprotroph
Animal Pathogen−Clavicipitaceous Endophyte−Fungal Parasite
Animal Pathogen−Endophyte−Lichen Parasite−Plant Pathogen−Soil Saprotroph−Wood Saprotroph
Endophyte−Lichen Parasite−Plant Pathogen−Undefined Saprotroph
Animal Pathogen−Endophyte−Fungal Parasite−Plant Pathogen−Wood Saprotroph
Bryophyte Parasite−Leaf Saprotroph−Soil Saprotroph−Undefined Saprotroph−Wood Saprotroph
Animal Pathogen−Dung Saprotroph−Endophyte−Lichen Parasite−Plant Pathogen−Undefined Saprotroph
Ectomycorrhizal−Fungal Parasite−Plant Pathogen−Wood Saprotroph
Endomycorrhizal−Plant Pathogen−Undefined Saprotroph
Undefined Saprotroph−Wood Saprotroph
Dung Saprotroph
Plant Pathogen−Wood Saprotroph
Dung Saprotroph−Plant Saprotroph
Epiphyte
Ectomycorrhizal−Undefined Saprotroph−Wood Saprotroph
Animal Pathogen−Endophyte−Plant Pathogen−Undefined Saprotroph
Dung Saprotroph−Soil Saprotroph−Undefined Saprotroph
Algal Parasite−Fungal Parasite−Undefined Saprotroph
Animal Pathogen−Lichen Parasite−Plant Pathogen−Undefined Saprotroph−Wood Saprotroph
Insect Pathogen
Lichenized−Undefined Saprotroph
Endophyte−Plant Pathogen−Undefined Saprotroph
Dung Saprotroph−Ectomycorrhizal−Litter Saprotroph−Undefined Saprotroph
Fungal Parasite−Plant Pathogen−Plant Saprotroph
Endophyte−Plant Pathogen
Plant Pathogen−Undefined Saprotroph
Dung Saprotroph−Plant Saprotroph−Soil Saprotroph
Dung Saprotroph−Undefined Saprotroph
Animal Pathogen−Endophyte−Epiphyte−Fungal Parasite−Plant Pathogen−Wood Saprotroph
Ectomycorrhizal
Dung Saprotroph−Wood Saprotroph
Wood Saprotroph
Dung Saprotroph−Endophyte−Plant Pathogen−Undefined Saprotroph
Plant Pathogen−Plant Saprotroph
Dung Saprotroph−Plant Parasite−Soil Saprotroph−Undefined Saprotroph−Wood Saprotroph
Fungal Parasite−Undefined Saprotroph
Lichenized
Animal Pathogen−Endophyte−Plant Pathogen−Dung Saprotroph−Undefined Saprotroph
Ectomycorrhizal−Fungal Parasite
Bryophyte Parasite−Ectomycorrhizal−Ericoid Mycorrhizal−Undefined Saprotroph
Animal Endosymbiont−Animal Pathogen−Undefined Saprotroph
Animal Pathogen−Endophyte−Plant Saprotroph−Soil Saprotroph
Arbuscular Mycorrhizal
Endophyte−Insect Pathogen
Animal Pathogen−Undefined Saprotroph
Animal Pathogen−Endophyte−Plant Pathogen−Wood Saprotroph
Bryophyte Parasite−Dung Saprotroph−Ectomycorrhizal−Fungal Parasite−Leaf Saprotroph−Plant Parasite−Undefined Saprotroph−Wood Saprotroph
Dung Saprotroph−Endophyte−Litter Saprotroph−Undefined Saprotroph
Dung Saprotroph−Soil Saprotroph
Soil Saprotroph
Ectomycorrhizal−Fungal Parasite−Soil Saprotroph−Undefined Saprotroph
Endophyte
Lichen Parasite−Lichenized
Animal Parasite−Fungal Parasite
Dung Saprotroph−Leaf Saprotroph
Litter Saprotroph−Soil Saprotroph−Wood Saprotroph
Leaf Saprotroph−Plant Pathogen−Undefined Saprotroph−Wood Saprotroph
Animal Endosymbiont−Animal Pathogen−Endophyte−Plant Pathogen−Undefined Saprotroph
Ectomycorrhizal−Undefined Saprotroph
Mode
HC_vs_OPL_fc
OPL_vs_OSCC_fc
HC_vs_OSCC_fc

Supplementary Figure 20. Functional alterations in the saliva mycobiome. The relative abundance of functional pathways was compared among HC, OPL, and OSCC individuals. Differentially abundant pathways were plotted, and the exact *P* values are presented in the heatmap. Generalized fold change is indicated by color gradients. The generalized fold change > 0: enriched in the latter; generalized fold change < 0: enriched in the former.

Supplementary Table 1 Baseline information of the HC, OPL and OSCC groups

**Characteristic**	**HC** (n=30)	**OPL** (n=32)	**OSCC** (n=29)	***F*** **/** χ^2	***P*** **value**
**Age**, y	56.63±11.12	56.00±13.67	61.97±10.11	2.295	0.107
**Gender**, No. (%)				1.338	0.512
Female	15(50.00)	19(59.37)	13(44.83)		
Male	15(50.00)	13(40.63)	16(55.17)		
**Smoking**, No. (%)				0.018	0.991
Yes	14(46.67)	15(46.88)	14(48.28)		
No	16(53.33)	17(53.12)	15(51.72)		
**Drinking**, No. (%)				0.169	0.919
Yes	16(53.33)	16(50.00)	16(55.17)		
No	14(46.67)	16(50.00)	13(44.83)		
**Dysplasia**, No. (%)					
No		13(40.63%)			
Low grade		13(40.63%)			
High grade		6(18.75%)			
**Differentiation**, No. (%)					
Middle grade			14(48.28%)		
High grade			15(51.72%)		
**pT stage**, No. (%)					
T1			7(24.14%)		
T2			10(34.48%)		
T3			6(20.69%)		
T4			6(20.69%)		
**pN stage**, No. (%)					
N0			19(65.52%)		
N1			6(20.69%)		
N2			4(13.79%)		

HC, healthy control. OPL, oral premalignant lesion. OSCC, oral squamous cell carcinoma. pT stage, pathological T stage. pN stage, pathological N stage. Quantitative data are presented as mean ± standard deviation; categorical data are presented as frequency and percentage.
